# Effectiveness of Anti-Inflammatory Agents to Prevent Atrial Fibrillation After Cardiac Surgery: A Systematic Review and Network Meta-Analysis

**DOI:** 10.1016/j.cjco.2024.10.008

**Published:** 2024-10-29

**Authors:** Alireza Malektojari, Zahra Javidfar, Sara Ghazizadeh, Shaghayegh Lahuti, Rahele Shokraei, Mohadeseh Zeinaee, Amirhosein Badele, Raziyeh Mirzadeh, Mitra Ashrafi, Fateme Afra, Mohammad Hamed Ersi, Marziyeh Heydari, Ava Ziaei, Zohreh Rezvani, Jasmine Mah, Dena Zeraatkar, Shahin Abbaszadeh, Tyler Pitre

**Affiliations:** a Cardiovascular Research Center, Hormozgan University of Medical Sciences, Bandar Abbas, Iran; b Evidence Based Medicine Center, Hormozgan University of Medical Sciences, Bandar Abbas, Iran; c Faculty of Medicine, Hormozgan University of Medical Sciences, Bandar Abbas, Iran; d Infectious and Tropical Diseases Research Center, Hormozgan University of Medical Sciences, Bandar Abbas, Iran; e Xi’an Jiaotong University Health Science Center, Xi’an, Shaanxi, China; f Department of Medicine, Dalhousie University, Halifax, Nova Scotia, Canada; g Department of Health Research Methods Evidence and Impact, McMaster University, Hamilton, Ontario, Canada; h Department of Anesthesiology, McMaster University, Hamilton, Ontario, Canada; i Division of Respirology, Department of Medicine, University of Toronto, Toronto, Ontario, Canada

## Abstract

**Background:**

Preventing postoperative atrial fibrillation (POAF) as one of the most significant complications of cardiovascular surgeries remains a major clinical challenge. We conducted a systematic review with network meta-analysis of randomized controlled trials, to identify the most effective and safe anti-inflammatory drugs to prevent new-onset POAF.

**Methods:**

MEDLINE, Embase, Web of Science, and Cochrane Library were searched without language or publication-date restriction on August 8, 2022 (updated on August 8, 2023). We assessed the risk of bias of included trials using the Cochrane risk-of-bias 2.0 tool. We conducted a frequentist random-effects network meta-analysis in R, and we assessed the certainty of evidence using the Grading of Recommendations, Assessment, Development, and Evaluations (GRADE) approach.

**Results:**

A total of 85 trials reported the incidence of new-onset POAF, including 18,981 patients. Use of nonsteroidal anti-inflammatory drugs (relative risk [RR] 0.37 [95% confidence interval [CI] 0.23-0.59]) and statins (RR 0.56 [95% CI 0.45-0.7]) potentially reduced the risk of POAF compared with placebo (both with a moderate certainty level). Use of fish oil in combination with vitamins C and E (RR 0.30 [95% CI 0.13-0.68]) may reduce the risk of POAF, compared with placebo (low level of certainty). Use of colchicine (RR 0.62 [95% CI 0.45- 0.85]), corticosteroids (RR 0.70 [95% CI 0.59-0.82]), and N-acetylcysteine (RR 0.69 [95% CI 0.49- 0.98]) may reduce the risk of POAF (all with a low level of certainty). None of the interventions had a significant effect on mortality rate or risk of serious adverse effects.

**Conclusions:**

Use of nonsteroidal anti-inflammatory drugs and statins probably are effective in preventing new-onset POAF, with a moderate level of certainty, compared to placebo.

## Résumé

**Contexte:**

La prévention de la fibrillation auriculaire postopératoire (FAPO), l'une des complications les plus importantes des chirurgies cardiovasculaires, reste un défi clinique majeur. Nous avons effectué une revue systématique avec une méta-analyse en réseau des essais contrôlés randomisés, afin d'identifier les médicaments anti-inflammatoires les plus efficaces et les plus sûrs pour prévenir l'apparition de nouvelle FAPO.

**Méthodes:**

Nous avons effectué des recherches dans MEDLINE, Embase, Web of Science et Cochrane Library, sans restriction de langue ou de date de publication, le 8 août 2022 (mise à jour le 8 août 2023). Nous avons évalué le risque de biais des essais cliniques sélectionnés à l'aide de l'outil Cochrane Risk-of-Bias 2.0. Nous avons effectué une méta-analyse avec un modèle fréquentiste à effet aléatoire dans R, et nous avons évalué le niveau de certitude des évidences en utilisant l'approche GRADE (Grading of Recommendations, Assessment, Development, and Evaluations).

**Résultats:**

Au total, 85 essais ont rapporté l'incidence de la FAPO d'apparition récente, incluant 18 981 patients. L'utilisation d'anti-inflammatoires non stéroïdiens (risque relatif [RR] 0,37 [intervalle de confiance [IC] à 95 % 0,23-0,59]) et de statines (RR 0,56 [IC à 95 % 0,45-0,7]) a potentiellement réduit le risque de FAPO par rapport au placebo (dans les deux cas avec un niveau de certitude modéré). L'utilisation d'huile de poisson en association avec les vitamines C et E (RR 0,30 [IC à 95 % 0,13-0,68]) pourrait réduire le risque de FAPO par rapport au placebo (niveau de certitude faible). L'utilisation de la colchicine (RR 0.62 [IC à 95 % 0,45- 0,85]), des corticostéroïdes (RR 0,70 [IC à 95 % 0,59-0,82]) et de la N-acétylcystéine (RR 0,69 [IC à 95 % 0,49- 0,98]) pourrait réduire le risque de FAPO (tous avec un faible niveau de certitude). Aucune des interventions n'a eu d'effet significatif sur le taux de mortalité ou le risque d'effets indésirables graves.

**Conclusions:**

L'utilisation d'anti-inflammatoires non stéroïdiens et de statines est probablement efficace pour prévenir l'apparition nouvelle d'une FAPO, avec un niveau de certitude modéré, par rapport au placebo.

Atrial fibrillation (AF) is the most common type of arrhythmia encountered after cardiac surgery.^1^ The incidence of new-onset postoperative atrial fibrillation (POAF) after cardiac surgeries has ranged from 18%-57% and is higher after combined coronary artery bypass grafting (CABG) and valve surgery than after CABG alone.^2^

New-onset POAF is considered to be one of the most significant complications of postcardiovascular surgeries.^3^ Generally, POAF occurs at a point between 24 and 96 hours postoperatively, with a peak incidence on the second postoperative day.^4^ Furthermore, complications of POAF include increased risk of stroke, morbidity and mortality, respiratory failure, and pneumonia.^5^

Cardiopulmonary bypass and ischemia-reperfusion injury in cardiac surgery can lead to a systemic inflammatory response.^6^ Patients with POAF have higher levels of inflammatory markers, such as C-reactive protein and inflammatory cytokines, compared to patients with normal sinus rhythm.^6^ Given that inflammation plays a significant role in the pathophysiology of POAF, several anti-inflammatory agents have been suggested to prevent AF in periprocedural settings.^7^

Clinical efforts to prevent and manage new-onset POAF following cardiac surgery have thus far presented a major challenge. Despite numerous trials examining prophylactic and treatment modalities, the incidence of POAF after cardiac surgery has not changed over the past several decades.^8^

The comparative effectiveness of anti-inflammatory agents to reduce new-onset POAF is uncertain. Head-to-head trials are not feasible for many of these interventions. A network meta-analysis (NMA) allows for head-to-head comparisons of treatments that have not been compared in randomized controlled trials (RCTs). No such NMA has been conducted on anti-inflammatory therapies for POAF. We aimed to conduct a systematic review and NMA of RCTs to identify which are the most effective and safest anti-inflammatory drugs to use to prevent AF in patients without a history of AF following cardiac surgery.

## Methods

### Standard reporting

We registered our protocol with Open Science Framework on June 27, 2022 (osf.io/56hx7), and we followed the Preferred Reporting Items for Systematic Reviews and Meta-Analyses (PRISMA) extension statement for NMA to report our findings.^9^^,^^10^ In our protocol, we planned to analyze all drug therapies available, but after assessing for incoherence and intransitivity, we plan now to report the interventions separately, based on class (ie, anti-inflammatory, anti-arrhythmic, etc.). In this first analysis, we report on anti-inflammatory agents.

### Searches

We conducted a systematic review of RCTs in MEDLINE, Embase, Web of Science Core Collection, and Cochrane Library on August 8, 2022, and we updated the search on August 8, 2023. The electronic search was developed and refined in MEDLINE. We performed a systematic search, without any language or publication-date restriction. Also, we reviewed reference lists from related guidelines, reviews, and eligible studies for additional eligible trials.^1^^,^^6^^,^^7^^,^^11^, ^12^, ^13^, ^14^, ^15^, ^16^, ^17^, ^18^, ^19^, ^20^, ^21^, ^22^, ^23^, ^24^, ^25^, ^26^, ^27^, ^28^, ^29^, ^30^, ^31^, ^32^, ^33^, ^34^, ^35^, ^36^, ^37^, ^38^, ^39^, ^40^, ^41^, ^42^, ^43^, ^44^, ^45^, ^46^, ^47^, ^48^, ^49^, ^50^, ^51^, ^52^, ^53^, ^54^, ^55^, ^56^

Supplemental Table S1 presents additional details, including the search terms for each database, the dates of searches, and an export strategy. Citations were imported into a reference manager, EndNote 20, and duplicate records were removed.^57^

### Eligibility criteria and study selection

We included peer-reviewed RCTs that enroll adults (aged ≥ 18 years) undergoing cardiac surgery (including CABG, valve surgery, or other major cardiac surgeries) without a history of AF or any other supraventricular arrhythmia, randomized to either anti-inflammatory agents including colchicine, corticosteroids, N-acetylcysteine (NAC), vitamins C and E, statins, nonsteroidal anti-inflammatory drugs (NSAIDs), and, fish oil vs standard care or one another. Supplemental Table S2 presents more details on the eligibility criteria.

Pairs of reviewers independently screened the titles, abstracts, and full-text articles of potentially eligible studies. We resolved disagreements by consensus, and when necessary, by adjudication with a third reviewer. We used the Rayyan online systematic review software (Rayyan—Intelligent Systematic Review; https://www.rayyan.ai/)^58^ to facilitate literature screening.

### Data extraction

Pairs of independent reviewers extracted relevant information from included studies. We performed calibration exercises to gauge consistency and precision between teams of reviewers. We developed standardized data-extraction forms to abstract the following information: (i) study characteristics (eg, authors, publication year, country of recruitment); (ii) characteristics of participants (eg, sample size, age, sex, underlying comorbidities); (iii) type of surgical procedure; and (iv) characteristics of interventions and comparators (eg, fFormulation, description, dosage, and time of intervention).

Outcomes of interest included all-cause mortality, the incidence of AF, the duration of hospitalization, and the occurrence of serious adverse events.

### Risk-of-bias assessment

Pairs of independent reviewers assessed the quality of included trials using the Cochrane risk-of-bias tool for randomized trials (2.0).^59^ We rated randomized trials across the following 5 domains: (i) randomization and allocation concealment process; (ii) blinding and deviation from intended intervention; (iii) loss to follow-up and missing outcome data; (iv) outcome measurement; (v) selection of the reported results (deviations from the registered protocol).^59^^,^^60^ Pair reviewers discussed conflicts regarding risk-of-bias assessment to come to a consensus, or consulted with an arbitrator if necessary.

### Data synthesis and statistical methods

For each outcome, we conducted a frequentist random-effects network meta-analysis using the restricted estimator of maximum likelihood (REML) with the netmeta package in R (version 4.03; R Foundation for Statistical Computing, Vienna, Austria).^61^ A network meta-analysis can be used to compare interventions that may not have been compared directly in clinical trials. A network meta-analysis produces network estimates from the pooled results of both direct (pairwise, conventional meta-analysis) and indirect evidence (drug treatments with common comparators). The network estimate therefore is informed by the entire network, allowing for indirect comparisons of drug treatments that were never compared in actual RCTs. We categorized drug treatments based on molecule and mechanism of action, unless evidence of significant clinical efficacy is present. To assess for incoherence, we used the node-splitting method (difference between direct and indirect evidence in closed loops).

We performed pairwise meta-analyses to estimate the direct estimates and assess for inconsistency. We assessed heterogeneity in the data via inspection of forest plots and the I^2^ statistic. We considered the level of heterogeneity to be as follows: 0%-40%, potentially unimportant; 30%-60%, moderate; 50%-90%, substantial; and 75%-100%, critical.^62^ For comparisons with ≥ 10 trials, we plan to assess publication bias by visual inspection of funnel plots and using Egger’s statistical test.

For visual presentation, we generated network and forest plots using the network map command in Stata, version 18 (StataCorp, College Station, TX).

We summarized the effects of interventions using relative risks (RRs) and corresponding 95% confidence intervals (CIs), and the absolute risk difference per 1000 patients, with a baseline risk sourced from the median risk in the placebo and standard-care arms across trials. For continuous outcomes, we reported mean differences with their associated 95% CIs.

We performed subgroup analyses to further investigate the sources of heterogeneity. We investigated whether the timing of receipt of interventions (treatment given preoperatively or throughout the surgery) would affect the treatment effects.

### Certainty of the evidence

We assessed the certainty of the evidence using the Grading of Recommendations, Assessment, Development, and Evaluations (GRADE)^63^ approach for network meta-analysis.^64^ We rated the certainty for each comparison and outcome as high, moderate, low, or very low, based on considerations of risk of bias, inconsistency, indirectness, publication bias, intransitivity, incoherence, and imprecision.

A minimally contextualized approach was used for judgements of imprecision, which considers whether CIs include a minimally important effect and does not consider the magnitude of plausible effects, captured by CIs.

We searched literature sources for validated minimal clinically important differences (MCIDs), when available, for specified outcomes and surveyed the authors when data were unavailable for appropriate MCIDs.

The results were reported using guidance from the Grading of Recommendations, Assessment, Development, and Evaluations Working Group, which involves using different adjectives based on the certainty of evidence (eg, this drug reduces mortality incidence [high certainty]; this drug probably reduces mortality incidence [moderate certainty]; this drug may reduce mortality incidence [low certainty]; and the effect of this drug on mortality incidence is very uncertain [very low certainty]).^65^

## Results

### Search results

Our search identified 3628 citations. After removing duplicate references, reviewers screened 2054 citations in the title- and abstract-screening stage and identified 333 citations for full-text review. Finally, we included 93 eligible RCTs examining 19,149 patients. Of the 93 studies that met our inclusion criteria, 2 had overlapping populations, 1 did not report data on POAF, and 5 trials examined only 2 similar arms from the same drug class (both arms included statin at different doses) that could not be included in our network meta-analysis. We excluded these 8 studies from the final analysis.

**Figure 1:**
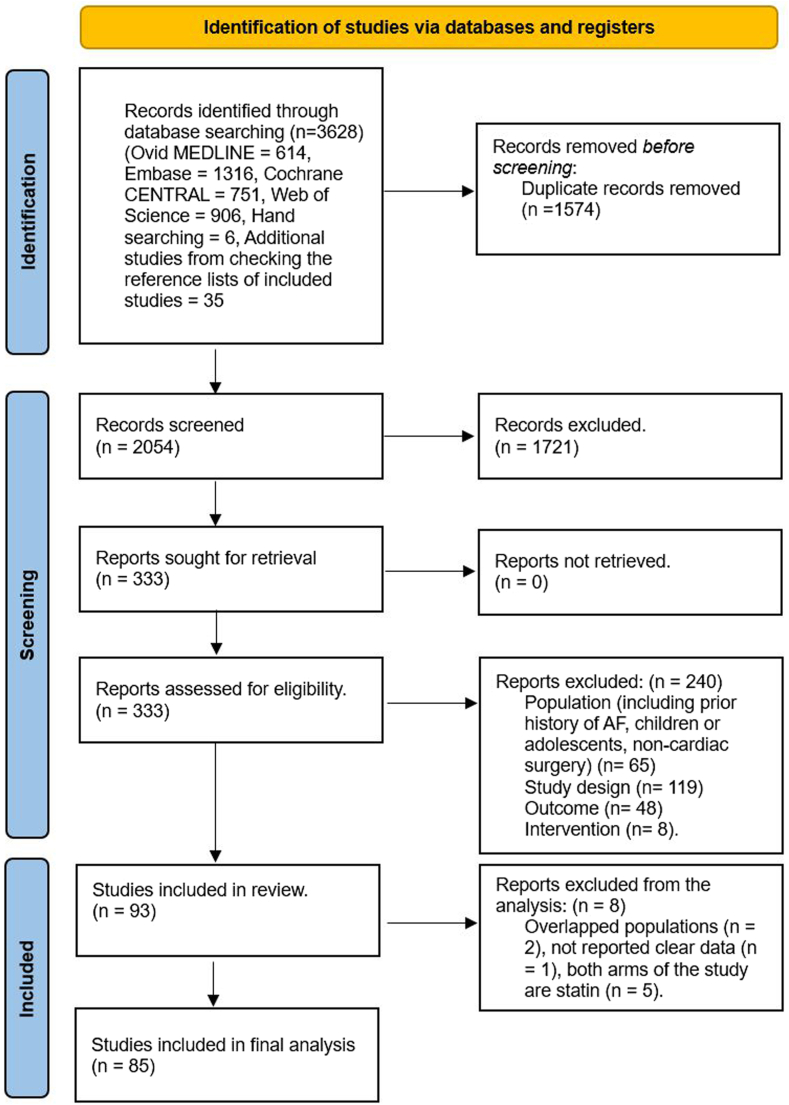
Preferred Reporting Items for Systematic Reviews and Meta-Analyses (PRISMA) flow diagram of included and excluded studies. AF, atrial fibrillation.

Figure 1 and Supplemental Table S3 present the list of included and excluded studies.

### Study characteristics

A total of 93 studies (including 19,149 patients) were included, with a mean age of 61.7 years. Approximately 72.9% of the patients who underwent cardiac surgery were male. The mean preoperative ejection fraction percentage was 53%, and the mean cardiopulmonary bypass time was 87.2 minutes for all patients. The mean percentages of hypertension, diabetes mellitus, myocardial infarction, and chronic obstructive pulmonary disease were 58.1%, 30.1%, 39.2%, and 11.6%, respectively.

Supplemental Tables S3 and S4 present more details on patient characteristics and interventions.

A total of 28 trials were reported that evaluated use of corticosteroids vs control (the following number of trials administered the indicated agent: 13, methylprednisolone; 10, dexamethasone; 3, hydrocortisone; 1, methylprednisolone with dexamethasone; and 1, methylprednisolone with hydrocortisone). A total of 14 trials were reported on statins vs control (12 trials administered atorvastatin, and 2 trials administered rosuvastatin). Three trials examined use of NSAIDs vs control (1 trial used ketorolac with ibuprofen, 1 used naproxen, and the other used aspirin with its NSAID dosage). Also, for RCTS, 17 examined fish oil, 6 colchicine, 9 NAC, 9 vitamin C, and 1 vitamin D, all compared with control groups.

### Risk of bias

For the incidence of POAF, 54 studies (58.1%) adequately generated their randomization process and allocation sequence concealment. A total of 28 studies (30.1%) had a credible registered protocol without any deviations from it. Overall, 22 studies were rated as having a low risk of bias, 37 as having some causes for concern, and 34 as having a high risk of bias. Supplemental Figures S1 and S2 provide more detail on our risk-of-bias 2.0 assessments for each outcome.

### Network meta-analysis

**Figure 2:**
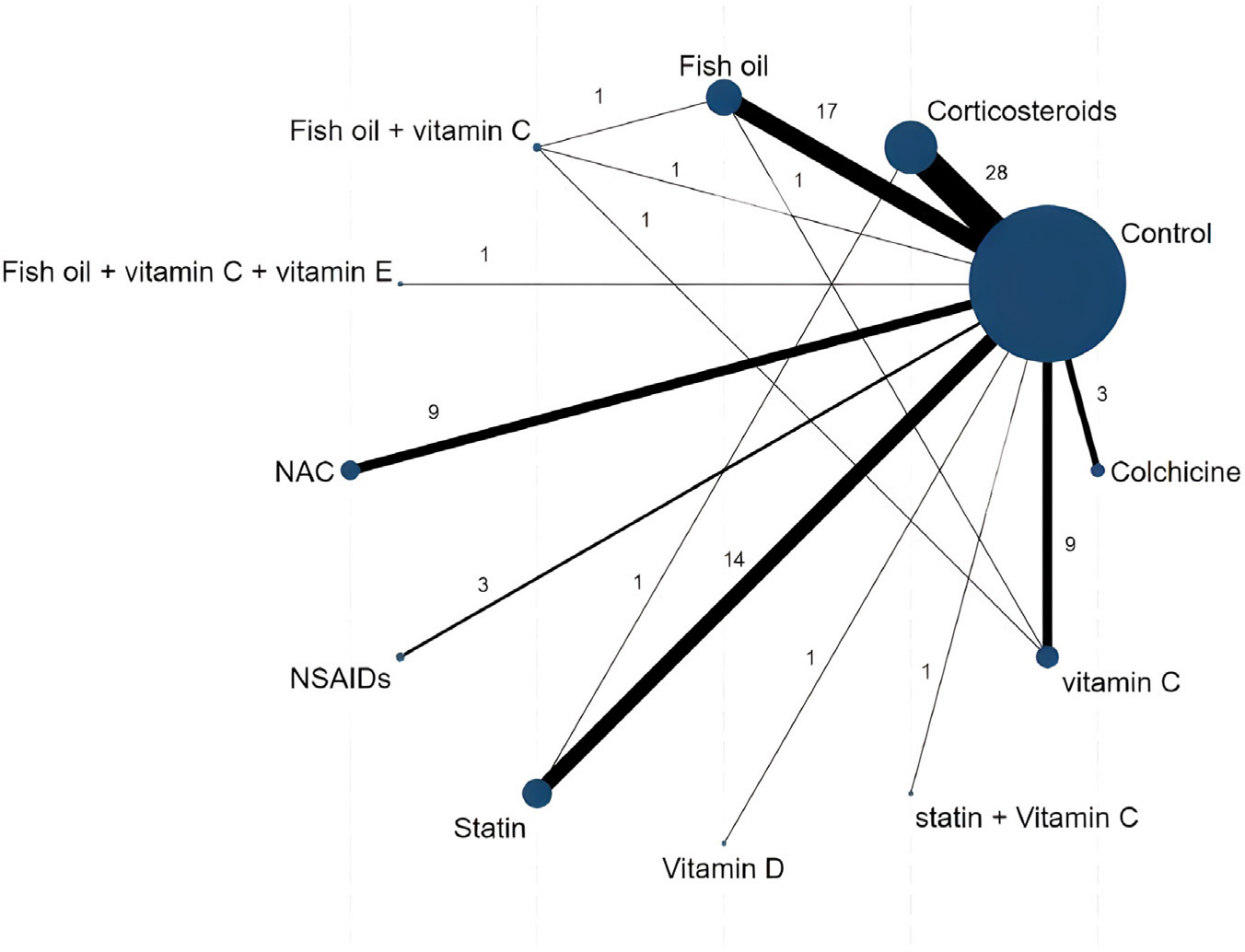
Network map of the treatments for postoperative atrial fibrillation (POAF) outcome. The size of the node (circle) corresponds to the number of patients randomized to the intervention. The thickness of the lines corresponds to the number of studies for each comparison. NAC, N-acetyl cysteine; NSAIDs, nonsteroidal anti-inflammatory drugs.

Our network was connected predominately via placebo, but several trials were head-to-head, resulting in multiple closed loops available to provide indirect evidence. Figure 2 and Supplemental Figures S3-S5 present the network geometry. As we found no difference in clinical efficacy with different delivery routes, we did not treat them as separate network nodes.

When assessing for incoherence, we did not find any evidence of incoherence using our node split models (Supplemental Figs. S6-S8). The level of network heterogeneity was moderate for the incidence of AF (I^2^, 45.3% [95% CI 28.7%-58.0%]), unimportant for mortality incidence (I^2^, 0% [95% CI 0.0%-33.5%]), unimportant for incidence of serious adverse events (I^2^, 0% [95% CI 0.0%-60.2%]), and substantial for the duration of hospitalization (I^2^, 88.4% [95% CI 85.8%-90.5%]). Supplemental Figures S9-S12 present the pairwise and network forest plots for each outcome. Supplemental Tables S5-S8 present the league tables for each network.

### Incidence of postoperative AF

A total of 85 trials reported on the incidence of POAF, including 18,981 patients and 5860 events.

We found that NSAIDs (RR 0.37 [95% CI 0.23-0.59]) and statins probably reduced (RR 0.56 [95% CI 0.45-0.70]) the risk of POAF, compared with placebo (both having a moderate certainty level).

Fish oil combination with vitamins C and E (RR 0.30 [95% CI 0.13-0.68]) may reduce the risk of POAF, compared with placebo (low certainty level). Colchicine (RR 0.62 [95% CI 0.45-0.85]), corticosteroids (RR 0.70 [95% CI 0.59-0.82]), and NAC (RR 0.69 [95% CI 0.49- 0.98]) may reduce the risk of POAF (all with a low certainty level).

**Figure 3:**
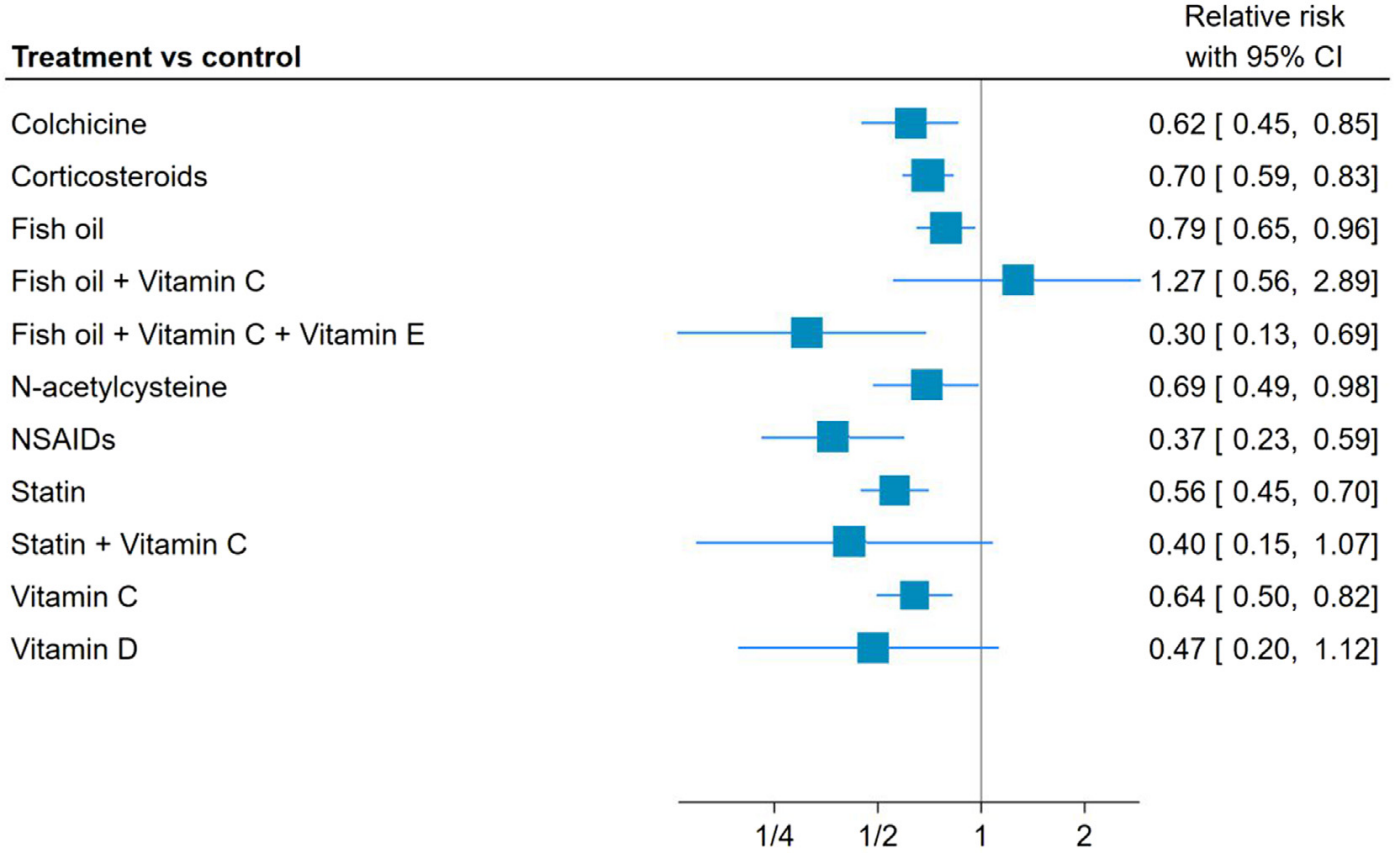
Network forest plot for incidence of postoperative atrial fibrillation. Presented with relative risk and 95% confidence intervals (95% CIs). NSAIDs, nonsteroidal anti-inflammatory drugs.

Figure 3 presents the network plot, and Table 1 presents the summary-of-the-findings table.

**Table 1:** Summary of findings table for head-to-head comparisons (network estimates)

Interventions	Incidence of POAF		Mortality		Serious adverse events		Duration of hospitalization	
	RD per 1000 (95% CI)	Certainty of evidence	RD per 1000 (95% CI)	Certainty of evidence	RD per 1000 (95% CI)	Certainty of evidence	RD per 1000 (95% CI)	Certainty of evidence
Colchicine	**–114 (–165 to –45)**	**Low;** May be more beneficial than comparison	–9.9 (–19.8 to 10.2)	**Very low;** Very uncertain	–3 (–47 to 698)	**Very low;** Very uncertain	–0.29 (–1.91 to 1.34)	**Very low;** Very uncertain
Corticosteroids	**–90 (–123 to –54)**	**Low;** May be more beneficial than comparison	–6.9 (–13.8 to 2.7)	**Low** (May be not convincingly different than comparison)	50 (–30.5 to 450)	**Very low;** Very uncertain	–0.75 (–1.5 to 0)	**Very low;** Very uncertain
Fish oil	**–63 (–105 to –15)**	**Very low;** Very uncertain	–6.9 (–18.9 to 18)	**Very low;** Very uncertain	36 (–25.5 to 262.5)	**Very low;** Very uncertain	**–1.09 (–2.1 to –0.09)**	**Low;** May be more beneficial than comparison
Fish oil + Vitamin C	81 (–132 to 570)	**Very low;** Very uncertain	NA		NA		NA	
NAC	**–93 (–153 to –6)**	**Low;** May be more beneficial than comparison	–15.9 (–25.5 to 13.5)	**Very low;** Very uncertain	–1 (–47 to 783.5)	**Low;** May be more harmful than comparison	–0.06 (–1.25 to 1.13)	**Low;** May be not convincingly different than comparison
NSAIDs	**–189 (–231 to –123)**	**Moderate;** Probably more beneficial than comparison	22.5 (–21.3 to 270)	**Very low;** Very uncertain	188 (–9.5 to 1200)	**Very low;** Very uncertain	1.86 (0.01 to 3.71)	**Very low;** Very uncertain
Statin	**–132 (–165 to –90)**	**Moderate;** Probably more beneficial than comparison	3.6 (–15.9 to 51)	**Very low;** Very uncertain	–1.5 (–43 to 283.5)	**Low;** May be more harmful than comparison	–3 (–47 to 698)	**Very low;** Very uncertain
Statin + Vitamin C	–180 (–225 to 24)	**Very low;** Very uncertain	NA		NA		NA	
Vitamin C	**–108 (–150 to –54)**	**Very low;** Very uncertain	3.6 (–29.4 to 1616.1)	**Very low;** Very uncertain	–2 (–49 to 2450)	**Low;** May be more harmful than comparison	–1.12 (–2.29 to 0.04)	**Low;** May be not convincingly different than comparison
Fish oil + Vitamin C + Vitamin E	**–210 (–261 to –96)**	**Low;** May be more beneficial than comparison	18.6 (–18 to 167.7)	**Very low;** Very uncertain	NA		–0.8 (–3.68 to 2.08)	**Low;** May be not convincingly different than comparison

All included estimates are network estimates, and most of the evidence is from indirect estimates. High certainty (very confident that true effect lies close to that of effect estimate), moderate certainty (moderately confident in effect estimate; the true effect is likely to be close to effect estimate, but there is a possibility that it is substantially different), low certainty (confidence in the effect estimate is limited; true effect may be substantially different from the effect estimate) or very low certainty (very little confidence in the effect estimate; true effect is likely to be substantially different from the effect estimate). Numbers presented in bold indicate statistical significance.

CI, confidence interval; NA, not applicable; NAC, N-acetyl cysteine; NSAIDs, nonsteroidal anti-inflammatory drugs; POAF, postoperative atrial fibrillation; RD, risk difference.

### All-cause mortality

A total of 54 studies reported on mortality, with 13,765 patients, including 250 deaths (1.8%). Supplemental Figure S2 shows the network geometry.

None of these interventions had a significant effect on the mortality rate (Supplemental Fig. S5). Corticosteroids had no effect on the risk of death, with a low level of certainty. Other treatments all had a very low level of certainty. Table 1 presents more details on the results.

### Duration of hospitalization

A total of 68 RCTs reported the hospitalization duration for 15,965 patients, with an average hospital stay of 9.9 days.

We found that use of fish oil may reduce the duration of hospital stay, compared with placebo (mean difference –1.09 [95% CI –2.10 to –0.09]; low level of certainty). The effects of use of colchicine, corticosteroids, NSAIDs, and statins on the duration of hospital stay had a very low level of certainty.

Fish oil combination with vitamins C and E, vitamin C alone, and NAC, may have no significant effect on length of hospital stay (all with a low level of certainty). Table 1 presents more details, and Supplemental Figure S4 shows the network geometry, and Supplemental Figure S7 shows the network forest plot.

### Serious adverse events

A total of 17 studies (2636 patients) reported the occurrence of serious adverse effects, including 22 events. None of the interventions had significantly increased the risk of occurrence of serious AEs (Table 1). Supplemental Figure S3 shows the network geometry, and Supplemental Figure S6 shows the network forest plot. All other adverse events reported in studies are summarized in Supplemental Table S9. Two studies reported patient readmission to the intensive care unit after discharge from the postsurgical intensive care unit. In both studies, 3 readmissions were reported in the corticosteroids group, vs no readmission in the control group (Supplemental Table S10).

### Heterogeneity and subgroups

Some of the heterogeneity may be explained by the timing of the intervention. We found a statistically significant subgroup in the pairwise analysis for fish oils, with a benefit in reducing POAF when given preoperatively and postoperatively, throughout, but not when given preoperatively (*P* < 0.001 for interaction). However, this benefit was not seen in any other comparison. We did not find any statistically significant subgroup differences based on the type of surgery performed.

## Discussion

### Main findings

Our NMA critically evaluates the comparative effectiveness and safety of various anti-inflammatory agents in preventing new-onset POAF, a common complication following cardiac surgery. The analysis incorporated data from 93 RCTs, ultimately including over 19,000 patients. The NMA results suggest that NSAIDs and statins probably are effective in reducing the incidence of POAF, with a moderate certainty level, compared to placebo. This finding is significant given the substantial burden POAF places on postoperative outcomes, including increased risks of stroke, morbidity, and mortality.^3^^,^^5^

Additionally, combinations of fish oil with vitamins C and E also may offer benefits in POAF prevention, albeit with a lower certainty level. Conversely, the analysis did not demonstrate a significant impact of corticosteroid use on mortality rates, suggesting that they have a limited role in POAF management strategies.

### In relation to previous findings

Our meta-analysis reveals that both statins and NSAIDs significantly lower the risk of new-onset POAF, with a moderate level of certainty of evidence. This finding aligns with previous findings suggesting that statin therapy is linked to a reduced risk of POAF.^43^ In addition, 2 other studies echo our results on statin use preventing POAF. ^20^^,^^44^ Some prior large RCTs and meta-analyses that included these RCTs have shown no effect of statins in preventing POAF. ^66^^,^^67^ These studies enrolled participants with or without a prior history of AF, a process that did not meet our inclusion criteria, as our objective was to evaluate the potential of drug use in preventing new-onset POAF, while avoiding any possible bias owing to participants' baseline conditions.

A peak in the incidence of AF occurred at 48-72 hours after heart surgery. This peak happens when the heart is typically under less stress and when an inflammatory process related to the surgery is occurring.^68^ Prior studies have indicated that inflammation and elevated levels of inflammatory markers, such as C-reactive protein, interleukin-6, and complement 3 and 4, may be associated with AF and atrial arrhythmias. Therefore, NSAID use can be associated with a lower incidence of POAF though this mechanism.^68^, ^69^, ^70^ Statins, also known as 3-hydroxy-3-methylglutaryl coenzyme A reductase inhibitors, are recognized for their antioxidant and anti-inflammatory properties. They help improve lipid metabolism and protect against atherosclerosis and endothelial dysfunction.^71^^,^^72^ Based on these properties, statins have been suggested to have the potential to prevent AF in patients undergoing cardiac surgery.^53^

Contrarily, colchicine use was found to not affect POAF, a conclusion based on limited studies, one of which included treatment 16 days postsurgery.^50^ Other analyses focusing solely on colchicine use for POAF prevention corroborate our findings.^7^^,^^33^^,^^34^ Corticosteroids, on the other hand, show a notable impact on reducing the incidence of POAF, albeit with a low level of certainty. This finding is consistent with results of other studies, although the optimal type, dose, and administration frequency of steroids remain unclear.^6^^,^^11^^,^^37^

Regarding NAC, our analysis is in line with existing meta-analyses that highlight the potential benefits for patients’ postcardiac surgery.^24^^,^^31^^,^^35^ The effectiveness of a therapy combination involving polyunsaturated fatty acid, vitamin C, and vitamin E is supported by our findings and other research with a moderate level of certainty.^25^ Although fish oil's role in reducing new-onset AF postsurgery has been significant in some studies,^25^^,^^27^ others have not found that it reduces recurrent AF effectively.^52^ This discrepancy could be attributed to differences in study focuses— specifically, our concentration on new-onset AF vs the broader approach used in other research, which might explain the variability in results.^73^

### Strengths and limitations

A key strength of this NMA is the comprehensive inclusion of available RCTs and the rigorous methodologic approach, following the Preferred Reporting Items for Systematic Reviews and Meta-Analyses (PRISMA) guidelines and employing advanced statistical analyses to synthesize direct and indirect comparisons. To prevent bias due to baseline comorbidity and reduce the heterogeneity, we only included those RCTs that enrolled participants without a history of AF before surgery. This breadth and depth of analysis offer a nuanced understanding of the relative efficacies of different anti-inflammatory interventions.

However, significant heterogeneity is present across the included trials, along with issues relating to risk of bias, which pose notable limitations, potentially affecting the generalizability and reliability of our findings. Furthermore, our analysis could not definitively pinpoint which patient subgroups might benefit most from specific anti-inflammatory interventions, indicating a need for more-targeted research. The observed heterogeneity also underscores the complexity of POAF as a multifactorial condition, influenced by surgical techniques, patient comorbidities, and perioperative management strategies. Notably, a significant proportion of patients enrolled had a diagnosis of chronic obstructive pulmonary disease, which may help explain the beneficial effects of anti-inflammatory agents, although the data are too sparse in our analysis to properly investigate this possibility.

We did attempt to reduce concerns with intransitivity by first limiting our analysis to anti-inflammatory agents only for this first report. However, other considerations include the age of participants, the type of surgery performed, the baseline risk of patients developing POAF in each study, and the way in which the interventions were given (ie, the dose, duration, and route). We did perform pairwise meta-regressions and subgroups, as appropriate, to look for evidence of intransitivity, and reassuringly, we did not find evidence of a credible subgroup effect.

### Clinical implementation

As the NMA results demonstrated a significant impact of statin and NSAID use in preventing new-onset POAF (moderate certainty level), as well as having a simplicity in administration and an absence of major complications, they can be considered suitable options for use in clinical settings.^53^^,^^74^ Although the safety of NSAIDs has been proven in postsurgical settings, they can also help reduce inflammation and postsurgical pain in patients who are undergoing cardiac surgeries.^74^ Use of statins is recommended in perioperative settings, owing to their easy administration and additional benefits. They have anti-inflammatory and antioxidant effects, improve lipid metabolism, and protect against endothelial dysfunction and atherosclerosis.^53^

On the other hand, although our NMA results showed that colchicine and corticosteroid use may reduce the incidence of new-onset POAF (all with a low level of certainty), use of colchicine and corticosteroids in the clinical setting is more challenging.^75^ The concerns about the potential side effects of colchicine and corticosteroid use make the decision about whether to use them as preventive agents for POAF challenging.^6^^,^^7^^,^^34^^,^^75^ This decision should be made based on the weighing, by expert specialists, of their potential positive effects and possible side effects in clinical settings.

### Future directions

Future research should prioritize RCTs that are designed to minimize bias and maximize comparability across different anti-inflammatory interventions. Studies focusing on specific patient populations, surgical procedures, and the timing of intervention administration could elucidate more-personalized approaches to POAF prevention. Additionally, exploring the mechanistic underpinnings of how anti-inflammatory agents influence atrial electrophysiology may uncover new therapeutic targets.

Moreover, given the evolving landscape of cardiac surgery and perioperative care, continuous re-evaluation of intervention effectiveness through updated NMAs will be crucial in adapting clinical guidelines to reflect the most-current evidence. Lastly, the integration of patient-reported outcomes and quality-of-life measures in future studies could provide a more holistic assessment of the impact of POAF prevention strategies, ensuring that patient-centred care remains at the forefront of cardiac surgery research and practice.

### Conclusion

This NMA presents the largest comparison of all types of anti-inflammatory agents being investigated for this purpose. Our analyses demonstrate that NSAID and statin use probably effectively reduce the incidence of new-onset POAF (with a moderate level of certainty), and fish oil may be beneficial in reducing the incidence of hospitalization (low level of certainty). Additional investments in new trials that address limitations of the current evidence may improve the certainty of evidence and allow us to make stronger claims about drugs that are effective for preventing AF after cardiac surgery.

## Supplementary Data

To access the supplementary material accompanying this article, visit *CJC Open* at https://www.cjcopen.ca/ and at https://doi.org/10.1016/j.cjco.2024.10.008.

## References

[1] Koniari I., Apostolakis E., Rogkakou C., Baikoussis N.G., Dougenis D. (2010). Pharmacologic prophylaxis for atrial fibrillation following cardiac surgery: a systematic review. J Cardiothorac Surg.

[2] Halonen J., Loponen P., Järvinen O. (2010). Metoprolol versus amiodarone in the prevention of atrial fibrillation after cardiac surgery: a randomized trial. Ann Intern Med.

[3] Echahidi N., Pibarot P., O’Hara G., Mathieu P. (2008). Mechanisms, prevention, and treatment of atrial fibrillation after cardiac surgery. J Am Coll Cardiol.

[4] Gu W.-J., Wu Z.-J., Wang P.-F., Aung L.H.H., Yin R.-X. (2012). Intravenous magnesium prevents atrial fibrillation after coronary artery bypass grafting: a meta-analysis of 7 double-blind, placebo-controlled, randomized clinical trials. Trials.

[5] Maaroos M., Tuomainen R., Price J. (2013). Preventive strategies for atrial fibrillation after cardiac surgery in Nordic countries. Scand J Surg.

[6] Liu L., Jing F.-Y., Wang X.-W. (2021). Effects of corticosteroids on new-onset atrial fibrillation after cardiac surgery: a meta-analysis of randomized controlled trials. Medicine (Baltimore).

[7] Salih M., Smer A., Charnigo R. (2017). Colchicine for prevention of post-cardiac procedure atrial fibrillation: meta-analysis of randomized controlled trials. Int J Cardiol.

[8] Greenberg J.W., Lancaster T.S., Schuessler R.B., Melby S.J. (2017). Postoperative atrial fibrillation following cardiac surgery: a persistent complication. Eur J Cardio-Thorac Surg.

[9] Hutton B., Salanti G., Caldwell D.M. (2015). The PRISMA extension statement for reporting of systematic reviews incorporating network meta-analyses of health care interventions: checklist and explanations. Ann Intern Med.

[10] Moher D., Liberati A., Tetzlaff J., Altman D.G., PRISMA Group (2009). Preferred reporting items for systematic reviews and meta-analyses: the PRISMA statement. Ann Intern Med.

[11] Ali-Hassan-Sayegh S., Mirhosseini S.J., Haddad F. (2015). Protective effects of corticosteroids in coronary artery bypass graft surgery alone or combined with valvular surgery: an updated and comprehensive meta-analysis and systematic review. Interact Cardiovasc Thorac Surg.

[12] Ali-Hassan-Sayegh S., Mirhosseini S.J., Rezaeisadrabadi M. (2014). Antioxidant supplementations for prevention of atrial fibrillation after cardiac surgery: an updated comprehensive systematic review and meta-analysis of 23 randomized controlled trials. Interact cardiovasc Thorac Surg.

[13] Arsenault K.A., Yusuf A.M., Crystal E. (2013). Interventions for preventing post-operative atrial fibrillation in patients undergoing heart surgery. Cochrane Database Syst Rev.

[14] Baker W.L., Coleman C.I. (2016). Meta-analysis of ascorbic acid for prevention of postoperative atrial fibrillation after cardiac surgery. Am J Health-Syst Pharm.

[15] Burgess D.C., Kilborn M.J., Keech A.C. (2006). Interventions for prevention of post-operative atrial fibrillation and its complications after cardiac surgery: a meta-analysis. Eur Heart J.

[16] Crystal E., Connolly S.J., Sleik K., Ginger T.J., Yusuf S. (2002). Interventions on prevention of postoperative atrial fibrillation in patients undergoing heart surgery: a meta-analysis. Circulation.

[17] DiDomenico R.J., Massad M.G. (2005). Pharmacologic strategies for prevention of atrial fibrillation after open heart surgery. Ann Thorac Surg.

[18] Dunning J., Khasati N., Prendergast B. (2004). What is the optimal medical treatment for stable cardiac surgical patients who go into atrial fibrillation after their operation?. Interact Cardiovasc Thorac Surg.

[19] Dvirnik N., Belley-Cote E.P., Hanif H. (2018). Steroids in cardiac surgery: a systematic review and meta-analysis. Br J Anaesth.

[20] Elgendy I.Y., Mahmoud A., Huo T., Beaver T.M., Bavry A.A. (2015). Meta-analysis of 12 trials evaluating the effects of statins on decreasing atrial fibrillation after coronary artery bypass grafting. Am J Cardiol.

[21] Epstein A.E., Alexander J.C., Gutterman D.D., Maisel W., Wharton J.M. (2005). Anticoagulation: American College of Chest Physicians guidelines for the prevention and management of postoperative atrial fibrillation after cardiac surgery. Chest.

[22] Fang W.T., Li H.J., Zhang H., Jiang S. (2012). The role of statin therapy in the prevention of atrial fibrillation: a meta-analysis of randomized controlled trials. Br J Clin Pharmacol.

[23] Ge P., Fu Y., Su Q. (2022). Colchicine for prevention of post-operative atrial fibrillation: meta-analysis of randomized controlled trials. Front Cardiovasc Med.

[24] Gu W.-J., Wu Z.-J., Wang P.-F., Htet Aung L.H., Yin R.-X. (2012). N-acetylcysteine supplementation for the prevention of atrial fibrillation after cardiac surgery: a meta-analysis of eight randomized controlled trials. BMC Cardiovasc Disord.

[25] Guo X.-Y., Yan X.-L., Chen Y.-W. (2014). Omega-3 fatty acids for postoperative atrial fibrillation: alone or in combination with antioxidant vitamins? Heart. Lung Circ.

[26] Harling L., Rasoli S., Vecht J.A. (2011). Do antioxidant vitamins have an anti-arrhythmic effect following cardiac surgery? A meta-analysis of randomised controlled trials. Heart.

[27] He Z., Yang L., Tian J. (2013). Efficacy and safety of omega-3 fatty acids for the prevention of atrial fibrillation: a meta-analysis. Can J Cardiol.

[28] Hindricks G., Potpara T., Dagres N. (2021). 2020 ESC guidelines for the diagnosis and management of atrial fibrillation developed in collaboration with the European Association for Cardio-Thoracic Surgery (EACTS): The Task Force for the diagnosis and management of atrial fibrillation of the European Society of Cardiology (ESC) Developed with the special contribution of the European Heart Rhythm Association (EHRA) of the ESC. Eur Heart J.

[29] Hu X., Yuan L., Wang H. (2017). Efficacy and safety of vitamin C for atrial fibrillation after cardiac surgery: a meta-analysis with trial sequential analysis of randomized controlled trials. Int J Surg.

[30] January C.T., Wann L.S., Calkins H. (2019). 2019 AHA/ACC/HRS focused update of the 2014 AHA/ACC/HRS guideline for the management of patients with atrial fibrillation: a report of the American College of Cardiology/American Heart Association Task Force on Clinical Practice Guidelines and the Heart Rhythm Society in collaboration with the Society of Thoracic Surgeons. Circulation.

[31] Khan S.A., Campbell A.M., Lu Y. (2021). N-Acetylcysteine for cardiac protection during coronary artery reperfusion: a systematic review and meta-analysis of randomized controlled trials. Front Cardiovasc Med.

[32] Langlois P.L., Hardy G., Manzanares W. (2017). Omega-3 polyunsaturated fatty acids in cardiac surgery patients: an updated systematic review and meta-analysis. Clin Nutr.

[33] Lee J.Z., Singh N., Howe C.L. (2016). Colchicine for prevention of post-operative atrial fibrillation: a meta-analysis. JACC Clin Electrophysiol.

[34] Lennerz C., Barman M., Tantawy M., Sopher M., Whittaker P. (2017). Colchicine for primary prevention of atrial fibrillation after open-heart surgery: systematic review and meta-analysis. Int J Cardiol.

[35] Liu X.-H., Xu C.-Y., Fan G.-H. (2014). Efficacy of N-acetylcysteine in preventing atrial fibrillation after cardiac surgery: a meta-analysis of published randomized controlled trials. BMC Cardiovasc Disord.

[36] Macle L., Cairns J., Leblanc K. (2016). 2016 focused update of the Canadian Cardiovascular Society guidelines for the management of atrial fibrillation. Can J Cardiol.

[37] Marik P.E., Fromm R. (2009). The efficacy and dosage effect of corticosteroids for the prevention of atrial fibrillation after cardiac surgery: a systematic review. J Crit Care.

[38] Mei M., Zhao H.-W., Pan Q.-G. (2018). Efficacy of N-acetylcysteine in preventing acute kidney injury after cardiac surgery: a meta-analysis study. J Invest Surg.

[39] Members W.C., Lawton J.S., Tamis-Holland J.E. (2022). 2021 ACC/AHA/SCAI guideline for coronary artery revascularization: a report of the American College of Cardiology/American Heart Association Joint Committee on Clinical Practice Guidelines. J Am Coll Cardiol.

[40] Ng K.T., Van Paassen J., Langan C. (2020). The efficacy and safety of prophylactic corticosteroids for the prevention of adverse outcomes in patients undergoing heart surgery using cardiopulmonary bypass: a systematic review and meta-analysis of randomized controlled trials. Eur J Cardio-Thorac Surg.

[41] Polymeropoulos E., Bagos P., Papadimitriou M. (2016). Vitamin C for the prevention of postoperative atrial fibrillation after cardiac surgery: a meta-analysis. Adv Pharm Bull.

[42] Putra B., Putra F. (2021). Expanding the potential benefits of colchicine for preventing postpericardiotomy syndrome and atrial fibrillation complications after cardiac surgery: meta-analysis of randomized controlled trials. Eur J Prev Cardiol.

[43] Rezaei Y., Gholami-Fesharaki M., Dehghani M.R. (2016). Statin antiarrhythmic effect on atrial fibrillation in statin-naive patients undergoing cardiac surgery: a meta-analysis of randomized controlled trials. J Cardiovasc Pharmacol Therapeut.

[44] Sai C., Li J., Ruiyan M., Yingbin X. (2019). Atorvastatin prevents postoperative atrial fibrillation in patients undergoing cardiac surgery. Hellen J Cardiol.

[45] Shi R., Li Z.H., Chen D. (2018). Sole and combined vitamin C supplementation can prevent postoperative atrial fibrillation after cardiac surgery: a systematic review and meta-analysis of randomized controlled trials. Clin Cardiol.

[46] Trivedi C., Sadadia M. (2014). Colchicine in prevention of atrial fibrillation following cardiac surgery: systematic review and meta-analysis. Indian J Pharmacol.

[47] Wang G., Bainbridge D., Martin J., Cheng D. (2011). N-acetylcysteine in cardiac surgery: Do the benefits outweigh the risks? A meta-analytic reappraisal. J Cardiothorac Vasc Anesth.

[48] Wang G., Niu J., Li Z., Lv H., Cai H. (2018). The efficacy and safety of dexmedetomidine in cardiac surgery patients: a systematic review and meta-analysis. PLoS One.

[49] Wang H., Chen J., Zhao L. (2018). N-3 polyunsaturated fatty acids for prevention of postoperative atrial fibrillation: updated meta-analysis and systematic review. J Interv Card Electrophysiol.

[50] Wang M.-X., Deng X-l, Mu B.-Y. (2016). Effect of colchicine in prevention of pericardial effusion and atrial fibrillation: a meta-analysis. Intern Emerg Med.

[51] Wang Z., Zhang Y., Gao M. (2011). Statin therapy for the prevention of atrial fibrillation: a meta-analysis of randomized controlled trials. Pharmacoth J Hum Pharmacol Drug Ther.

[52] Xin W., Wei W., Lin Z. (2013). Fish oil and atrial fibrillation after cardiac surgery: a meta-analysis of randomized controlled trials. PLoS One.

[53] Yuan X., Du J., Liu Q., Zhang L. (2017). Defining the role of perioperative statin treatment in patients after cardiac surgery: a meta-analysis and systematic review of 20 randomized controlled trials. Int J Cardiol.

[54] Zhao J., Li M., Tan C. (2022). Efficacy of N-acetylcysteine in preventing acute kidney injury and major adverse cardiac events after cardiac surgery: a meta-analysis and trial sequential analysis. Front Med.

[55] Zhou A.-G., Wang X.-X., Pan D.-B. (2017). Preoperative antihypertensive medication in relation to postoperative atrial fibrillation in patients undergoing cardiac surgery: a meta-analysis. BioMed Res Int.

[56] Zimmer J., Pezzullo J., Choucair W. (2003). Meta-analysis of antiarrhythmic therapy in the prevention of postoperative atrial fibrillation and the effect on hospital length of stay, costs, cerebrovascular accidents, and mortality in patients undergoing cardiac surgery. Am J Cardiol.

[57] Bramer W.M., Milic J., Mast F. (2017). Reviewing retrieved references for inclusion in systematic reviews using EndNote. J Med Libr Assoc.

[58] Ouzzani M., Hammady H., Fedorowicz Z., Elmagarmid A. (2016). Rayyan—a web and mobile app for systematic reviews. Syst Rev.

[59] Sterne J.A.C., Savović J., Page M.J. (2019). RoB 2: a revised tool for assessing risk of bias in randomised trials. BMJ.

[60] Guyatt G., Rennie D., Meade M., Cook D. (2002).

[61] Balduzzi S., Rücker G., Nikolakopoulou A. (2023). netmeta: an R package for network meta-analysis using frequentist methods. J Stat Software.

[62] Higgins J.P.T., Thomas J., Chandler J. (2024). Cochrane Handbook for Systematic Reviews of Interventions version 6.5 (updated August 2024).

[63] Brignardello-Petersen R., Mustafa R.A., Siemieniuk R.A. (2019). GRADE approach to rate the certainty from a network meta-analysis: addressing incoherence. J Clin Epidemiol.

[64] Guyatt G., Oxman A.D., Akl E.A. (2011). GRADE guidelines: 1. Introduction—GRADE evidence profiles and summary of findings tables. J Clin Epidemiol.

[65] Santesso N., Glenton C., Dahm P. (2020). GRADE guidelines 26: informative statements to communicate the findings of systematic reviews of interventions. J Clin Epidemiol.

[66] Putzu A., Capelli B., Belletti A. (2016). Perioperative statin therapy in cardiac surgery: a meta-analysis of randomized controlled trials. Crit Care.

[67] Billings F.T., Hendricks P.A., Schildcrout J.S. (2016). High-dose perioperative atorvastatin and acute kidney injury following cardiac surgery: a randomized clinical trial. JAMA.

[68] Cheruku K.K., Ghani A., Ahmad F. (2004). Efficacy of nonsteroidal anti-inflammatory medications for prevention of atrial fibrillation following coronary artery bypass graft surgery. Prev Cardiol.

[69] Bruins P., Velthuis Ht, Yazdanbakhsh A.P. (1997). Activation of the complement system during and after cardiopulmonary bypass surgery: postsurgery activation involves C-reactive protein and is associated with postoperative arrhythmia. Circulation.

[70] Chung M.K., Martin D.O., Sprecher D. (2001). C-reactive protein elevation in patients with atrial arrhythmias: inflammatory mechanisms and persistence of atrial fibrillation. Circulation.

[71] Pinho-Gomes A.C., Reilly S., Brandes R.P., Casadei B. (2014). Targeting inflammation and oxidative stress in atrial fibrillation: role of 3-hydroxy-3-methylglutaryl-coenzyme a reductase inhibition with statins. Antioxidants Redox Signal.

[72] Camm A.J., European Heart Rhythm Association; European Association for Cardiothoracic Surgery (2010). Guidelines for the management of atrial fibrillation: the Task Force for the Management of Atrial Fibrillation of the European Society of Cardiology (ESC). Eur Heart J.

[73] Liu T., Korantzopoulos P., Shehata M. (2011). Prevention of atrial fibrillation with omega-3 fatty acids: a meta-analysis of randomised clinical trials. Heart.

[74] Bongiovanni T., Lancaster E., Ledesma Y. (2021). Systematic review and meta–analysis of the association between non-steroidal anti–inflammatory drugs and operative bleeding in the perioperative period. J Am Coll Surg.

[75] Whitlock R.P., Chan S., Devereaux P. (2008). Clinical benefit of steroid use in patients undergoing cardiopulmonary bypass: a meta-analysis of randomized trials. Eur Heart J.

