# Supplementary material for: Effectiveness of Anti-Inflammatory Agents to Prevent Atrial Fibrillation After Cardiac Surgery: A Systematic Review and Network Meta-Analysis
Source: CJC Open. 2024 Oct 29;7(1):35–45. doi: 10.1016/j.cjco.2024.10.008 (PMC11763850; doi:10.1016/j.cjco.2024.10.008)
Supplement: Supplementary Materials [file mmc1.pdf]

Contents

__Supplemental Table S1; Search strategy for all databases 9
Supplemental Table S2; Summary of the PICOS and selection criteria 2
Supplemental Table S3; Table of characteristics 9
Supplemental Table S4; Table of interventions 14
Supplemental Figure S1; Traffic light plots for ROB 2.0 risk of Bias assessment 22
Supplemental Figure S2; Overall risk of Bias assessment for ROB 2.0 domains 23
Supplemental Figure S3; Network map of the treatments for all-cause mortality 24
Supplemental Figure S4; Network map of the treatments for serious adverse events 25
Supplemental Figure S5; Network map of the treatments for duration of hospitalization 26
Supplemental Figure S6; Indirect estimates for incidence of postoperative atrial fibrillation 27
Supplemental Figure S7; Indirect estimates for incidence of postoperative all-cause mortality 28
Supplemental Figure S8; Indirect estimates for duration of hospitalization 29
Supplemental Figure S9; Pairwise incidence of postoperative atrial fibrillation 36
Supplemental Figure S10; Pairwise incidence of postoperative all-cause mortality 38
Supplemental Figure S11; Pairwise incidence of postoperative serious adverse events 40
Supplemental Figure S12; Pairwise estimates for duration of hospitalization 41
Supplemental Figure S13; Pairwise estimates for duration of hospitalization 42
Supplemental Table S5; League table: Network meta-analysis results for incidence of postoperative atrial fibrillation 44
Supplemental Table S6; League table: Network meta-analysis results for postoperative all-cause mortality 45
Supplemental Table S7; League table: Network meta-analysis results for postoperative serious adverse events 46
Supplemental Table S8; League table: Network meta-analysis results for duration of hospitalization 47
Supplemental Table S9; Adverse events due to interventions 48
Supplemental Table S10; ICU readmission 53
References 54
Supplemental Appendix S1; PRISMA NMA Checklist of Items to Include When Reporting A Systematic Review Involving a Network Meta-analysis 62

Supplemental Table S1; Search strategy for all databases

**OVID Medline Epub Ahead of Print, In-Process & Other Non-Indexed Citations, Ovid MEDLINE(R) Daily and Ovid MEDLINE(R) 1946 to Present**
Database: Ovid MEDLINE(R) ALL <1946 to August 08, 2022><br>Search Strategy:<br>--<br>1 Atrial Fibrillation/ (66805)<br>2 ((atrial or atrium) adj (fibrillation* or arrhythmia* or tachyarrhythmia*)).tw,kf. (88419)<br>3 1 or 2 (100616)<br>4 Cardiac Surgical Procedures/ (58947)<br>5 ((heart or cardiac or aortic or thoracic or cardiovascular or cardiothoracic) adj1 (surg* or operat* or procedure* or repair*)).tw,kf. (119718)<br>6 (((coronary artery or cardio-pulmonary or cardiopulmonary) adj1 (surg* or operat* or graft*)) or CABG*).tw,kf. (23713)<br>7 (((valve* or valvular) adj1 (surg* or operat* or procedure* or repair* or replac*)) or valvuloplasty).tw,kf. (58167)<br>8 4 or 5 or 6 or 7 (203864)<br>9 Colchicine/ or colchicine.tw,kf. (21823)<br>10 Dexmedetomidine/ or dexmedetomidine.tw,kf. (7985)<br>11 Magnesium Compounds/ or magnesium.tw,kf. (67872)<br>12 Digoxin/ or digoxin.tw,kf. (15574)<br>13 Adrenergic beta-Antagonists/ or (beta-blocker* or (beta adj1 antagonist*) or adrenergic block*).tw,kf. (67951)<br>14 (sotalol or metoprolol or atentolol or emolol or bisoprolol).ti,ab. (11387)<br>15 Amiodarone/ or Amiodarone.tw,kf. (11614)<br>16 Adrenal Cortex Hormones/ or corticosteroid*.tw,kf. (155226)<br>17 (diltiazem or verapamil).ti,ab. (28711)<br>18 Anti-Inflammatory Agents, Non-Steroidal/ or (nsaid* or ((non-steroid* or nonsteroid*) adj anti-inflammatory)).tw,kf. (96296)<br>19 Fatty Acids, Unsaturated/ or fatty acid*.tw,kf. (257166)<br>20 Thiazolidinediones/ or Thiazolidinedione*.tw,kf. (14273)<br>21 Angiotensin-Converting Enzyme Inhibitors/ or (ace adj inhibitor).tw,kf. (37767)<br>22 Ascorbic Acid/ (44230)<br>23 ((glucose adj insulin adj potassium) or Vernakalant or ibutilide or disopyramide or propafenone or atorvastatin or Ranolazine or Disopyramide or Quinidine or Flecainide or Dofetilide).ti,ab. (23805)<br>24 Procainamide/ or Procainamide.tw,kf. (4479)<br>25 ((N-acetyl adj1 cysteine) or n-acetylcysteine).tw,kf. (16863)<br>26 Calcium Channel Blockers/ or calcium antagonist*.tw,kf. (43125)<br>27 9 or 10 or 11 or 12 or 13 or 14 or 15 or 16 or 17 or 18 or 19 or 20 or 21 or 22 or 23 or 24 or 25 or 26 (866575)<br>28 Randomized Controlled Trials as Topic/ or randomized controlled trial/ or Random Allocation/ or Double Blind Method/ or Single Blind Method/ or exp Clinical Trial/ (1198059)<br>29 ((singl* or doubl* or tripl* or trebl*) adj3 (blind* or mask* or method* or procedure*)).ti,ab,kf. (258030)<br>30 (((clinical or control*) adj2 trial*) or random*).tw,kf. (1665291)<br>31 28 or 29 or 30 (2284616)<br>32 3 and 8 and 27 and 31 (591)<br>***************************

EMBASE

Database: Embase <1974 to 2022 August 10>
Search Strategy:
----
1 atrial fibrillation/ (91529)
2 ((atrial or atrium) adj (fibrillation* or arrhythmia* or tachyarrhythmia*)).tw,kf. (157141)
3 1 or 2 (182019)
4 heart surgery/ (91082)
5 ((heart or cardiac or aortic or thoracic or cardiovascular or cardiothoracic) adj1 (surg* or operat* or procedure* or repair*)).tw,kf. (183066)
6 (((coronary artery or cardio-pulmonary or cardiopulmonary) adj1 (surg* or operat* or graft*)) or CABG*).tw,kf. (41301)
7 (((valve* or valvular) adj1 (surg* or operat* or procedure* or repair* or replac*)) or valvuloplasty).tw,kf. (85765)
8 4 or 5 or 6 or 7 (306505)
9 colchicine/ or colchicine.tw,kf. (38273)
10 dexmedetomidine/ or dexmedetomidine.tw,kf. (16332)
11 magnesium/ or magnesium.tw,kf. (123115)
12 digoxin/ or digoxin.tw,kf. (48957)
13 beta adrenergic receptor blocking agent/ or (beta-blocker* or (beta adj1 antagonist*) or adrenergic block*).tw,kf. (168050)
14 (sotalol or metoprolol or atentolol or emolol or bisoprolol).ti,ab. (17846)
15 amiodarone/ or amiodarone.tw,kf. (42827)
16 corticosteroid/ or corticosteroid*.tw,kf. (332540)
17 calcium channel blocking agent/ or calcium antagonist*.tw,kf. (79516)
18 (diltiazem or verapamil).ti,ab. (35574)
19 nonsteroid antiinflammatory agent/ or (nsaid* or ((non-steroid* or nonsteroid*) adj anti-inflammatory)).tw,kf. (166861)
20 unsaturated fatty acid/ or fatty acid*.tw,kf. (292119)
21 2,4 thiazolidinedione derivative/ or Thiazolidinedione*.tw,kf. (17827)
22 dipeptidyl carboxypeptidase inhibitor/ or (ace adj inhibitor).tw,kf. (130704)
23 ascorbic acid/ (99836)
24 ((glucose adj insulin adj potassium) or Vernakalant or ibutilide or disopyramide or propafenone or atorvastatin or Ranolazine or Disopyramide or Quinidine or Flecainide or Dofetilide).ti,ab. (34453)
25 procainamide/ or procainamide.tw,kf. (12380)
26 ((N-acetyl adj1 cysteine) or n-acetylcysteine).tw,kf. (21954)
27 9 or 10 or 11 or 12 or 13 or 14 or 15 or 16 or 17 or 18 or 19 or 20 or 21 or 22 or 23 or 24 or 25 or 26 (1430652)
28 Randomized Controlled Trials as Topic/ or randomized controlled trial/ or randomization/ or Double Blind Procedure/ or Single Blind Procedure/ or exp Clinical Trial/ or exp controlled clinical trial/ (1965671)
29 ((singl* or doubl* or tripl* or trebl*) adj3 (blind* or mask* or method* or procedure*)).ti,ab,kw. (368560)
30 (((clinical or control*) adj2 trial*) or random*).tw,kw. (2282118)
31 28 or 29 or 30 (3348348)
32 3 and 8 and 27 and 31 (1220)

****

CENTRAL

ID Search Hits
#1 MeSH descriptor: [1] this term only 5214
#2 ((atrial or atrium) NEAR (fibrillation* or arrhythmia* or tachyarrhythmia*)):ti,ab,kw 14960
#3 #1 or #2 14960
#4 MeSH descriptor: [Thoracic Surgery] this term only 177
#5 ((heart or cardiac or aortic or thoracic or cardiovascular or cardiothoracic) NEAR/1 (surg* or operat* or procedure* or repair*)):ti,ab,kw 18922
#6 (((coronary artery or cardio-pulmonary or cardiopulmonary) NEAR/1 (surg* or operat* or graft*)) or CABG*):ti,ab,kw 9194
#7 (((valve* or valvular) NEAR/1 (surg* or operat* or procedure* or repair* or replac*)) or valvuloplasty):ti,ab,kw 4499
#8 #4 or #5 or #6 or #7 27932
#9 MeSH descriptor: [Colchicine] this term only 416
#10 MeSH descriptor: [Dexmedetomidine] this term only 2174
#11 MeSH descriptor: [Magnesium] this term only 1239
#12 MeSH descriptor: [Digoxin] this term only 788
#13 MeSH descriptor: [Adrenergic beta-Antagonists] this term only 4442
#14 (colchicine or dexmedetomidine or magnesium or digoxin or amiodarone or corticosteroid* or (fatty NEAR acid*) or Thiazolidinedione* or procainamide):ti,ab,kw 64275
#15 (beta-blocker* or (beta NEAR/1 antagonist*) or adrenergic block*):ti,ab,kw 13967
#16 (sotalol or metoprolol or atentolol or emolol or bisoprolol or diltiazem or verapamil):ti,ab 7693
#17 MeSH descriptor: [Amiodarone] this term only 667
#18 MeSH descriptor: [Adrenal Cortex Hormones] this term only 2569
#19 MeSH descriptor: [Anti-Inflammatory Agents, Non-Steroidal] this term only 6788
#20 (nsaid* or ((non-steroid* or nonsteroid*) NEAR anti-inflammatory)):ti,ab,kw 15199
#21 MeSH descriptor: [Fatty Acids, Unsaturated] this term only 761
#22 MeSH descriptor: [Thiazolidinediones] this term only 1271
#23 MeSH descriptor: [Angiotensin-Converting Enzyme Inhibitors] this term only 4116
#24 (ace NEAR inhibitor):ti,ab,kw 2539
#25 MeSH descriptor: [Ascorbic Acid] this term only 2366
#26 ((glucose NEAR insulin NEAR potassium) or Vernakalant or ibutilide or disopyramide or propafenone or atorvastatin or Ranolazine or Disopyramide or Quinidine or Flecainide or Dofetilide):ti,ab 7368
#27 MeSH descriptor: [Procainamide] this term only 131
#28 ((N-acetyl adj1 cysteine) or n-acetylcysteine):ti,ab,kw 1840
#29 #9 or #10 or #11 or #12 or #13 or #14 or #15 or #16 or #17 or #18 or #19 or #20 or #21 or #22 or #23 or #24 or #25 or #26 or #27 or #28 111135
#30 #3 AND #8 and #29 717
****

Web of Science

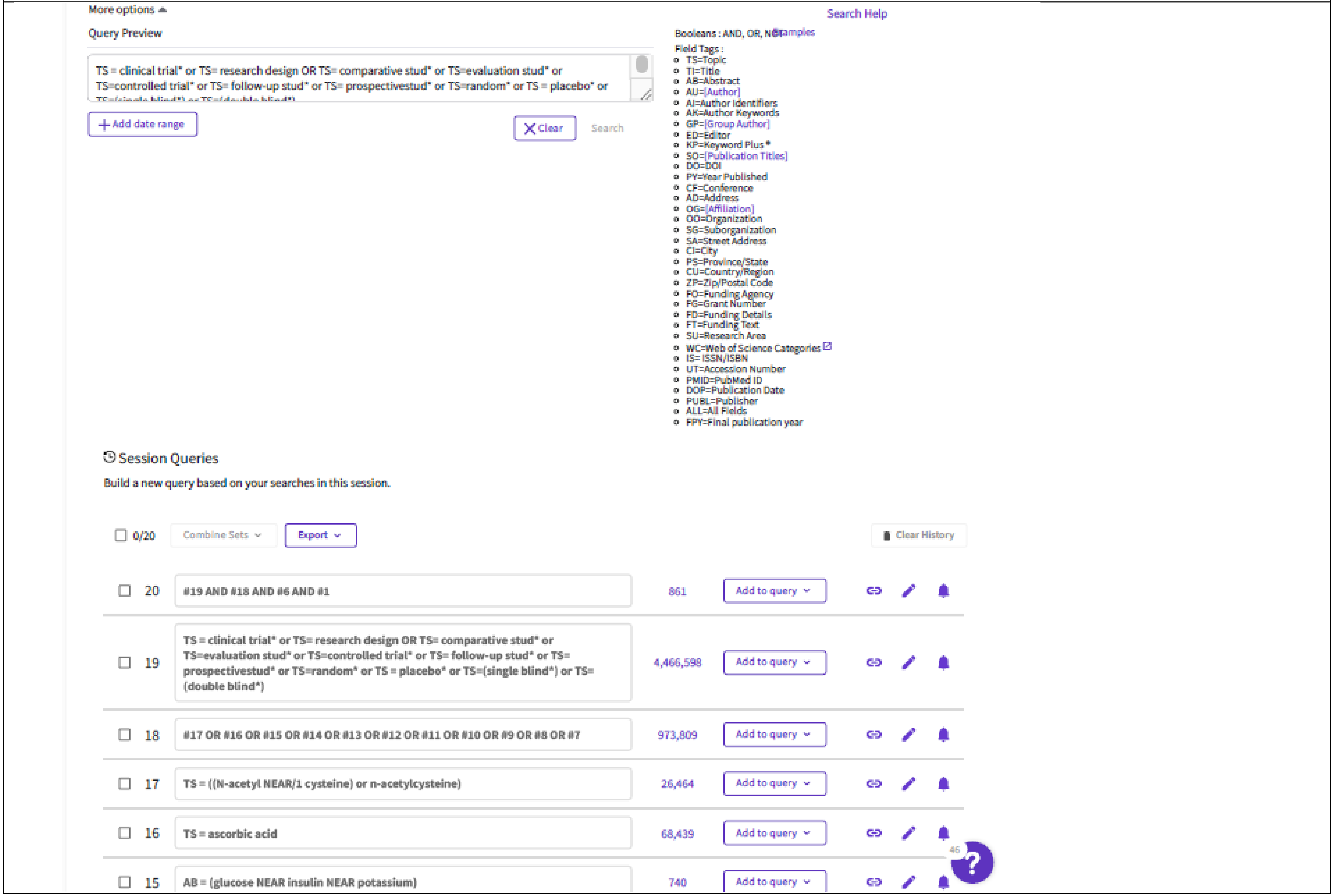

14	TI = (glucose NEAR insulin NEAR potassium)	502	Add to query
13	TS = "ace inhibitor"	8,666	Add to query
12	TS = "angiotensin converting enzyme inhibitor"	8,283	Add to query
11	TS = (nsaid* or ((non-steroid* or nonsteroid*) NEAR anti-inflammatory))	46,857	Add to query
10	AB = (sotalol or metoprolol or atentolol or emolol or bisoprolol or diltiazem or verapamil or Vernakalant or ibutilide or disopyramide or propafenone or atorvastatin or Ranolazine or Disopyramide or Quinidine or Flecainide or Dofetilide)	39,081	Add to query
9	TI = (sotalol or metoprolol or atentolol or emolol or bisoprolol or diltiazem or verapamil or Vernakalant or ibutilide or disopyramide or propafenone or atorvastatin or Ranolazine or Disopyramide or Quinidine or Flecainide or Dofetilide)	33,277	Add to query
8	TS = (beta-blocker* or (beta NEAR/1 antagonist*) or adrenergic block*)	55,496	Add to query
7	TS = (colchicine or dexmedetomidine or magnesium or digoxin or amiodarone or corticosteroid* or "calcium antagonist*" or "fatty acid*" or "calcium channel block*" or Thiazolidinedione or procainamide)	731,969	Add to query
6	#2 OR #3 OR #4 OR #5	213,888	Add to query
5	TS = (((valve* or valvular) NEAR/1 (surg* or operat* or procedure* or repair* or replac*)) or valvuloplasty)	58,319	Add to query
4	TS = cabg*	17,332	Add to query
3	TS = (("coronary artery" or "cardio-pulmonary" or cardiopulmonary) NEAR/1 (surg* or operat* or graft*))	43,267	Add to query
2	TS = ((heart or cardiac or aortic or thoracic or cardiovascular or cardiothoracic) NEAR/1 (surg* or operat* or procedure* or repair*))	144,822	Add to query
1	TS = ((atrial or atrium) NEAR (fibrillation* or arrhythmia* or tachyarrhythmia*))	110,339	Add to query

Hand searching

1. Martinez EA, Bass EB, Zimetbaum P; American College of Chest Physicians. Pharmacologic control of rhythm: American College of Chest Physicians guidelines for the prevention and management of postoperative atrial fibrillation after cardiac surgery. Chest. 2005 Aug;128(2 Suppl):48S-55S. doi: 10.1378/chest.128.2_suppl.48s. PMID: 16167665.
2. National Guideline Centre (UK). Treatment strategies for atrial fibrillation after cardiothoracic surgery: Atrial fibrillation: diagnosis and management: Evidence review L. London: National Institute for Health and Care Excellence; 2021 Apr. (NICE Guideline, No. 196.)
3. P.R. Kowey, J.E. Taylor, S.J. Rials, R.A. Marinchak. Meta-analysis of the effectiveness of prophylactic drug therapy in preventing supraventricular arrhythmia early after coronary artery bypass grafting Am J Cardiol, 69 (1992), pp. 963-965
4. Conte SM, Florisson DS, De Bono JA, Davies RA, Newcomb AE. Management of atrial fibrillation after cardiac surgery. Intern Med J. 2019 May;49(5):656-658. doi: 10.1111/imj.14281. PMID: 31083803.
5. Echahidi N, Pibarot P, O'Hara G, Mathieu P. Mechanisms, prevention, and treatment of atrial fibrillation after cardiac surgery. J Am Coll Cardiol. 2008 Feb 26;51(8):793-801. doi: 10.1016/j.jacc.2007.10.043. PMID: 18294562.
6. Arsenault KA, Yusuf AM, Crystal E, Healey JS, Morillo CA, Nair GM, Whitlock RP. Interventions for preventing post-operative atrial fibrillation in patients undergoing heart surgery. Cochrane Database of Systematic Reviews 2013, Issue 1. Art. No.: CD003611. DOI: 10.1002/14651858.CD003611.pub3.

Articles that were included after hand searching the above 6 guidelines/reviews:

1) McAlister HF, Luke RA, Smith WM. Amiodarone versus quinidine reversion of atrial fibrillation post-cardiac surgery. Australian and New Zealand Journal of Medicine. 1986; 16:588
2) Taenaka N, Kikawa S. The effectiveness and safety of landiolol hydrochloride, an ultra-short-acting beta1-blocker, in postoperative patients with supraventricular tachyarrhythmias: a multicenter, randomized, double-blind, placebo-controlled study. American Journal of Cardiovascular Drugs. 2013; 13(5):353–364 [PMC free article] [PubMed]
3) Wafa SS, Ward DE, Parker DJ, Camm AJ. Efficacy of flecainide acetate for atrial arrhythmias following coronary artery bypass grafting. American Journal of Cardiology. 1989; 63(15):1058–1064 [PubMed]
4) Yilmaz AT, Demirkilic U, Arslan M, Kurulay E, Ozal E, Tatar H et al. Long-term prevention of atrial fibrillation after coronary artery bypass surgery: comparison of quinidine, verapamil, and amiodarone in maintaining sinus rhythm. Journal of Cardiac Surgery. 1996; 11(1):61–64 [PubMed]

Search update; August 8, 2023

August 8, 2022		August 8, 2023		New
Ovid MEDLINE(R) ALL <1946 to August 08, 2022>	591	Ovid MEDLINE(R) ALL <1946 to August 08, 2022>	614	23
Embase <1974 to 2022 August 10	1220	Embase <1974 to 2022 August 10	1316	96 (99 – according to search)
Cochrane CENTRAL	713	Cochrane CENTRAL	751	38
Web of Science	861	Web of Science	906	45 (39 according to search)
Hand searching	4	Hand searching	6	2

Supplemental Table S2; Summary of the PICOS and selection criteria

<u>Summary of the PICOS and selection criteria</u>

**Population**	Any population of adult subjects (18 years or older) who have had any type of major cardiac surgery, without history of AF or supraventricular arrhythmia, included in a randomized controlled trial
**Interventions**	**Pre-identified prophylaxis or treatments;**<br>Colchicine, Corticosteroids, NSAIDs, N-acetyl-Cysteine, Statin, polyunsaturated fatty acids (fish oil), and, Ascorbic acid. Any newly identified anti-inflammatory intervention also considered.
**Comparison**	Any control or placebo group, routine care, no intervention, or comparison of pre-identified prophylaxis or treatments mentioned above.
**Outcome**	**Main outcomes;**<br>Incidence of atrial fibrillation following cardiac surgery (POAF),<br>All-cause mortality<br>**Additional outcomes;**<br>Overall hospital length of stay,<br>Serious adverse events and side effects due to interventions
**Settings**	Hospitalized patients undergoing elective or emergent cardiac surgery

Supplemental Table S3; Table of characteristics

ID	Name	Country	Male %	Age	Type of surgery	Colchicine use (%) at the Baseline	Beta-Blocker use (%) at Baseline	HTN %	DM %	MI %	COPD %	CPB time (min)	Preop EF%
**219**	Abbaszadeh-2012[2]	Iran	70	60	CABG	NR	NR	NR	NR	NR	NR	NR	NR
**250**	Abd El-Hakeem-2003[3]	Egypt	55	35	Valve	NR	NR	NR	NR	NR	NR	91	54
**218**	Abdallah-2019[4]	Egypt	54	29	Valve	NR	NR	NR	NR	NR	NR	123	60
**1**	Al-Shawabkeh-2017[5]	Jordan	51	65	CABG, CABG + Valve	NR	84.7	54.5	31.8	20	7.1	103	NR
**251**	Amr-2009[6]	Egypt	71	68	CABG	NR	25	NR	NR	NR	NR	109	More than 40
**210**	Aydin-2015[7]	Turkey	78.3	63	CABG	NR	60	56.6	40	NR	NR	96	NR
**207**	Baran-2011[8]	Turkey	62	61	CABG	NR	50	61.6	30	NR	35	79	49
**269**	Berger-2013[9]	Switzerland	89.3	66	CABG, CABG + Valve	NR	0	NR	NR	NR	NR	97	NR
**252**	Bingol-2005[10]	Turkey	77.5	64	CABG	NR	NR	40	NR	67.5	100	68	56
**198**	Calo-2005[11]	Italy	85	66	CABG	NR	57.5	80	32.5	52.5	16.2	99	56
**197**	Carrascal-2016[12]	Spain	65.6	67	Valve +CABG, Valve	NR	0	57.8	19.7	0	12.1	102	64
**270**	Castillo-2010[13]	Chile	71.6	60	CABG + Valve	NR	59.75	34.7	51.5	NR	NR	90	55
**253**	Celik-2004[14]	Turkey	NR	61	CABG	NR	NR	58.3	NR	NR	0	95	55
**254**	Chaney-1998[15]	USA	75	67	CABG	NR	58.3	NR	NR	NR	NR	100	Less than 40
**255**	Chaney-2001[16]	USA	75.6	65	CABG	NR	55.6	NR	NR	NR	NR	106	Less than 40
**268**	Chello-2006[17]	Italy	77.5	65	CABG	NR	32.5	45	0	NR	NR	96	NR
**5**	Cheruku-2004[18]	USA	65	65	CABG	NR	93	59	27	NR	3	NR	NR
**184**	Dehghani-2014[19]	Iran	74	61	CABG	NR	NR	30	24	21	0	116	43
**185**	Dehghani-2015[20]	Iran	33	49*	Valve	NR	NR	26	26	NR	0	120	45
**227**	Dieleman-2012[21]	Netherlands	72.5	66	CABG, Valve, CABG + Valve	NR	66	53	19	8	NR	124	NR
**272**	El-Hamamsy-2007[22]	Canada	89	61	CABG	NR	82	NR	NR	39.5	NR	71	NR
**181**	Elmarsafawi-2016[23]	Egypt	77.7	58	CABG	NR	NR	76.6	55.3	8.5	NR	130	62
**257**	Enc-2006[24]	Turkey	100	58	CABG	NR	NR	NR	0	NR	NR	79	NR

**273**	Erdil-2016[25]	Turkey	84.2	59	CABG	NR	NR	32.7	21.25	73	8.75	NR	49
**274**	Eren-2003[26]	Turkey	75	61	CABG	NR	NR	NR	NR	NR	0	102	51
**179**	Erkut-2019[27]	Turkey	50.3	63	CABG	NR	100	64.3	31.9	NR	NR	53	47
**178**	Eslami-2007[28]	Iran	67	60	CABG	NR	100	44	32	41	0	92	50
**175**	Farahani-2017[29]	Iran	64.6	61	CABG	NR	47.6	62.6	50	51.2	NR	88	43
**174**	Farquharson-2011[30]	Australia	73.2	64	CABG, Valve, CABG + Valve	NR	41.2	77.8	31.4	35.1	10.8	85	65
**8**	Feguri-2017[31]	Brazil	67	62	CABG	NR	63.2	NR	31.6	50.9	NR	71	57
**173**	Feguri-2019[32]	Brazil	67.7	62	CABG	NR	63.2	NR	31.6	50.9	NR	71	57
**258**	Gomez Polo-2017[33]	Spain	73.07	64	CABG, Valve, CABG + Valve	NR	100	NR	NR	NR	NR	NR	NR
**163**	Halonen-2007[34]	Finland	76.7	65	CABG, Valve, CABG + Valve	NR	84.55	63.1	25.3	NR	2.9	NR	61
**161**	Halvorsen-2003[35]	Norway	79.6	64	CABG	NR	86.1	NR	NR	NR	NR	NR	69
**159**	Heidarsdottir-2010[36]	Iceland	79	67*	CABG, Valve	NR	76.2	63.1	15.5	NR	NR	NR	60
**158**	Heidt-2009[37]	Germany	69	67	CABG	NR	NR	NR	NR	NR	NR	NR	52
**156**	Horbach-2011[38]	Brazil	63	59	CABG	NR	89.4	82.6	30.4	43.5	5.6	88	49
**154**	Jacob-2015[39]	Netherlands	76	70	CABG, Valve	NR	67.7	61.3	16.1	17.7	NR	104	NR
**152**	Ji-2009[40]	China	69.2	65	CABG	NR	62.1	29.2	37.8	17.8	32.1	NR	56
**151**	Joss-2017[41]	USA	73.5	66	CABG, Valve, CABG + Valve	NR	58.3	60.5	29.1	NR	NR	NR	NR
**146**	Kazemi-2013[42]	Iran	74.55	60	CABG, Valve, CABG + Valve, ASD, VSD	NR	97.45	60.4	39.4	46.2	NR	87	47
**259**	Kilger-2011[43]	Germany	23.6	68	CABG	NR	NR	NR	NR	NR	NR	0	58
**275**	Kim-2011[44]	South Korea	89.6	63	CABG	NR	47.9	68.8	58.3	45.8	0	NR	34
**271**	Kolesnikov-2015[45]	Russia	82.2	56	CABG	NR	71.4	71.1	13.6	NR	NR	NR	56
**140**	Kourliouros-2011[46]	UK	87.2	66	CABG, Valve, CABG + Valve	NR	64.7	67.6	19.6	52.9	7.8	65	NR
**260**	Lomivorotov-2012[47]	Russia	84	58	CABG	NR	NR	NR	NR	NR	0	62	NR
**132**	Lomivorotov-2014[48]	Russia	94.9	60	CABG	NR	71.8	NR	NR	53.8	0	64	62
**267**	Mannacio-2008[49]	Italy	72.5	60	CABG	NR	70.5	23	0	23	NR	82	NR
**22**	Mansour-2016[50]	Egypt	70	58	CABG	NR	NR	84	44	60	26	NR	NR

**261**	Mardani-2013[51]	Iran	86	62	CABG, CABG + Valve, IABP	NR	NR	21	14	0.1	0.03	NR	52
**25**	Mirhosseini-2010[52]	Iran	70.80 %	62	CABG	NR	NR	43.35	0	NR	17.5	NR	NR
**128**	Mirmohammadsadeghi-2018[53]	Iran	77.7	62	CABG	NR	NR	NR	NR	NR	NR	NR	NR
**126**	Moludi-2016[54]	Iran	65.9	56	CABG	NR	NR	43.1	22.1	NR	NR	91	41
**276**	Orhan-2006[55]	Turkey	65	61	CABG	NR	NR	45	40	30	0	66	69
**111**	Ozaydin-2008[56]	Turkey	79	58	CABG, Valve, CABG + Valve	NR	90.4	57.4	32.2	23	NR	99	51
**110**	Papoulidis-2010[57]	Greece	70.6	72	CABG	NR	100	58.2	19.4	NR	0	52	45
**108**	Patti-2006[58]	Italy	73.5	66	CABG, Valve, CABG + Valve, Aortic aneurysm repair	NR	66	86.5	37	42.5	32	109	52
**106**	Pierri-2016[59]	Italy	83.5	67	CABG	NR	79.2	69.8	39.6	NR	8.5	78	57
**105**	Prasongsukarn-2005[60]	Canada	76	64*	CABG	NR	NR	58	NR	65.11	18.6	NR	44
**102**	Rodrigo-2013[61]	Greece-Chile	85	60*	CABG, Valve, CABG + Valve	NR	61.5	30	59	NR	12	Median	Median
**32**	Rubanenko-2015[62]	Russia	84.7	62	CABG	NR	81.9	98	17.1	100	NR	NR	58
**99**	Rubens-2005[63]	Canada	86.8	55	CABG	NR	NR	54.4	21	NR	0	NR	NR
**294**	Sadeghpour-2014[64]	Iran	65.8	44	CABG, Valve, CABG + Valve, Congenital	NR	NR	43.1	22	NR	NR	91	NR
**33**	Samadikhan-2014[65]	Iran	68.3	61	CABG	NR	70.8	NR	NR	NR	NR	NR	NR
**96**	Saravanan-2010[66]	UK	79.6	66*	CABG	NR	85.4	32	14.5	25.2	8.7	80	NR
**95**	Saravanan-2016[67]	UK	78.6	66*	CABG	NR	90.1	22.9	9.8	21.3	NR	NR	NR
**94**	Sarzaeem-2014[68]	Iran	69.4	59	CABG	NR	NR	53.5	37.6	NR	0	NR	54
**295**	Sarzaeem-2014[69]	Iran	72.2	60	CABG	NR	NR	53.7	37.5	NR	1.9	NR	47
**262**	Schurr-2001[70]	Switzerland	86	62	CABG	NR	NR	NR	NR	NR	NR	75	61
**263**	Sobieski-2008[71]	USA	82	63	CABG	NR	NR	82	18	NR	NR	69	NR
**84**	Soleimani-2018[72]	Iran	51.7	61	CABG	NR	NR	62.4	40	15.6	NR	40	NR
**82**	Song-2008[73]	South Korea	65.32	63	CABG	NR	71	58	49.2	10.5	NR	0	58
**39**	Sorice-2011[74]	Italy	81.5	63	CABG	NR	60	64.7	42.2	NR	33.2	NR	53
**40**	Stanger-2014[75]	Austria	90.7	66	CABG	NR	83.8	80	23.3	36.2	13.4	89	NR

**264**	Suezawa-2013[76]	Japan	76.6	69	CABG	NR	23	50	43	NR	NR	NR	NR
**80**	Sun-2011[77]	China	67	65	CABG	0	62	31	38	NR	NR	89	55
**42**	Tabbalat-2015[78]	Jordan	79.9	61	CABG, Non CABG	NR	NR	64.4	49.4	23.6	NR	NR	55
**41**	Tabbalat-2020[79]	Jordan	76.6	59	CABG Non CABG	NR	NR	58.5	44	22.3	NR	NR	57
**265**	Vukovic-2010[80]	Serbia	84.8	61	CABG	NR	81.3	86	33.7	NR	NR	83	26
**65**	Wang-2016[81]	China	60	52*	CABG, Valve	NR	27.33	27.33	34	26	NR	NR	NR
**67**	Wang-2020[82]	China	38.57	57	CABG, Valve, Congenital	NR	NR	52.85	42.8	NR	NR	79	NR
**64**	Weis-2009[83]	Germany	58.33	68*	NR	NR	NR	NR	NR	NR	33.33	102	30
**277**	Wijeysundera-2007[84]	Canada	59.4	74	CABG, Valve, CABG + Valve	NR	67.4	77.7	32.6	NR	12	105	NR
**47**	Wilbring-2014[85]	Germany	85.3	68	CABG	NR	87.3	98	41.9	100	4.04	NR	NR
**296**	Yamamoto-2014[86]	Japan	59	71	CABG, Valve, VSD	NR	0	86	NR	NR	NR	NR	65
**59**	Yared-2000[87]	USA	82.4	63*	CABG, Valve, CABG + Valve	NR	NR	NR	0	75.4	NR	113	NR
**60**	Yared-2007[88]	USA	78.9	72	CABG + Valve	NR	52.5	NR	NR	NR	NR	114	NR
**53**	Zarpelon-2016[89]	Brazil	67.85	61	CABG	NR	51.42	NR	51.1	22.8	NR	NR	NR
**266**	Zheng-2016[90]	China	79.25	59	CABG, Valve, CABG + Valve	NR	84.1	64.3	31.3	29	1	NR	61
**323**	Talasaz-2021	Iran	73	61	CABG	NR	83	53.57	52.5	35.7	3.5	NR	43
**325**	Samadifar-2023	Iran	75	60	CABG	NR	82	52.5	27	NR	NR	NR	44
**326**	Shvartz-2022	Russia	81	62	CABG	NR	77	94	22.7	44.5	4	120	59.8
**327**	Shvartz-2022	Russia	75	62	CABG	NR	74	91	21	40	6	107	60

Table: NR: not reported, IV: intravenous, *: reported in median, Valve: valve surgeries, CABG: coronary artery bypass grafting, ASD: atrial septal defect surgery, VSD: ventricular septal defect surgery, USA

Supplemental Table S4; Table of interventions

ID	Study	Intervention name	Intervention description
**219**	Abbaszadeh-2012	Corticosteroids	Dexamethasone, 6g/mL, IV
		Placebo	Saline, 1mL
**250**	Abd El-Hakeem-2003	Corticosteroids	Dexamethasone, 100mg, IV, on the morning of the surgery day
		Placebo	Normal saline, 100mg, on the morning of the surgery day
**218**	Abdallah-2019	Statin	Atorvastatin, 80mg, PO, 12 and 2 hours preoperatively, and on the 2nd, 3rd, 4th, and 5th postoperative days
		Placebo	Placebo at the same time periods
**1**	Al-Shawabkeh-2017	Corticosteroids	Methylprednisolone, 1g, IV, before CPB and Hydrocortisone, 100mg, every 8 hours, 3 days postoperatively
		Placebo	Maintenance fluids
**251**	Amr-2009	Corticosteroids	Dexamethasone, 1mg/kg, at induction of anaesthesia and 0.5mg/kg 8 hours later
		Placebo	Placebo same amount of isotonic sodium chloride at the same time points
**210**	Aydin-2015	Statin	Atorvastatin, 40mg, PO, 6 hours after the operation
		No treatment	Usual care
**207**	Baran-2011	Statin	Atorvastatin, 40mg, PO, daily, 14days
		Placebo	Matching placebo same dose, at the same times
**269**	Berger-2013	Omega3/Omega6/PUFA	Fish oil, 0.2g/kg, lipid infusion, 12 hour before and 2 hour after surgery
		Placebo	Saline, 0.2g/kg, IV, 12 and 2 hours before and immediately after surgery
**252**	Bingol-2005	Corticosteroids	Prednisolone, 20mg/kg, PO,10 days before surgery until discharge
		Placebo	Matching placebo same dose, at the same times
**198**	Calo-2005	Omega3/Omega6/PUFA	PUFAs 2 g/day, PO, 5days before surgery until discharge
		No treatment	Usual care
**197**	Carrascal-2016	Statin	Atorvastatin 40mg/day, administered 7 days prior and after the surgery, until the 7th postoperative day.
		No treatment	Usual care
**270**	Castillo-2010	Omega3/Omega6/PUFA + Vitamins	PUFAs 2g/day, PO ,7 days before surgery until discharge. And vitamins C 1g/day and E 400IU/day, PO, added 2 days before surgery and until hospital discharge.
		Placebo	Matching placebo same dose, at the same times
**253**	Celik-2004	Corticosteroids	Methylprednisolone sodium succinate, 30mg/kg IV, six times preoperatively: 10 min before CPB, immediately after CBP and then every 6 hours for the next 24 hours.
		Placebo	Normal saline, IV
**254**	Chaney-1998	Corticosteroids	Methylprednisolone 30mg/kg, IV during sternotomy and 30mg/kg during the initiation of CPB.

		Placebo	Similar volumes of IV isotonic sodium chloride solution at the same two times.
**255**	Chaney-2001	Corticosteroids	Methylprednisolone 30mg/kg, IV, intra operatively
		Corticosteroids	Methylprednisolone, 15mg/kg, IV, intraoperatively
		Placebo	Matching placebo same dose, at the same times
**268**	Chello-2006	Statin	Atorvastatin ,20mg/day, PO, during the 3 weeks before surgery
		Placebo	Matching placebo same dose, at the same times
**5**	Cheruku-2004	NSAIDs	Ketorolac, 30mg, IV every 6h until able to take oral medications, at which point switched to ibuprofen, 600mg, PO, three times a day.
		No treatment	Usual care
**184**	Dehghani-2014	Ascorbic acid	Vitamin C, 2g, PO, before the surgery and 500mg twice daily for 5 days after the surgery.
		No treatment	Usual care
**185**	Dehghani-2015	Statin	Atorvastatin, 40mg, PO, 3days preoperatively and 5 days postoperatively.
		Placebo	Matching placebo same dose, at the same times
**227**	Dieleman-2012	Corticosteroids	Dexamethasone, 1mg/kg of body weight, with a 100 mg maximum, IV, single dose intraoperatively.
		Placebo	Matching placebo same dose, at the same times
**272**	El-Hamamsy-2007	N-acetyl-Cysteine	NAC, 600 mg, PO, the day before and the morning of the operation. Additionally, NAC a bolus of 150mg/kg of IV over a 15minute period immediately before skin incision, followed by perfusion at 12.5mg/kg/h over 24 hours.
		Placebo	Matching placebo same dose, at the same times
**181**	Elmarsafawi-2016	Statin	Atorvastatin 80mg/day for 2 days preoperatively. The same preoperative doses were restarted postoperatively and continued for one month.
		Statin	Atorvastatin 80mg/day for 5-9 days preoperatively. The same preoperative doses were restarted postoperatively and continued for one month.
		Statin	Atorvastatin 40mg/day for 5-9 days preoperatively. The same preoperative doses were restarted postoperatively and continued for one month.
**257**	Enc-2006	Corticosteroids	Methylprednisolone 25mg/kg IV 1h before CPB
		Placebo	Saline same dose, at the same times
**273**	Erdil-2016	N-acetyl-Cysteine	NAC, 600mg/day, PO, preoperatively for 3 days. NAC, 300mg, in the prime solution.
		Placebo	Matching placebo same dose, at the same times
**274**	Eren-2003	N-acetyl-Cysteine	NAC, 100mg/kg, IV, for one hour before CPB and 40mg/kg per day at 24 hours after CPB.
		Placebo	Saline in same dose, at the same times
**179**	Erkut-2019	NSAIDs	Aspirin 300mg/day
		No treatment	Usual care
**178**	Eslami-2007	Ascorbic acid	Effervescent ascorbic acid, 1g, PO, on the night before surgery, followed by 1g twice daily for 5 days postoperatively.

		No treatment	Usual care
**175**	Farahani-2017	Omega3/Omega6/PUFA	Fish oil, 2g/d, PO, 5days before surgery
		Placebo	Olive oil
**174**	Farquharson-2011	Omega3/Omega6/PUFA	Fish oil. 15ml/day, PO, 3weeks before surgery and 6days after or until discharge.
		Placebo	High monounsaturated sunflower oil, 15 mL/day
**8**	Feguri-2017	carbohydrates	8 h fast for solids; 2 h fast plus 200mL oral intake of 12.5% maltodextrin [25g] in water; no intraoperative ω-3 PUFA.
		Placebo	8 h fast for solids; 2 h fast plus 200mL oral intake of water only; no intraoperative ω-3 PUFA.
		Omega3/Omega6/PUFA + Other (carbohydrates)	8 h fast for solids; 2 h fast plus 200 mL oral intake of 12.5% maltodextrin [25 g] in water; intraoperative infusion of ω-3 PUFA [0.2mcg/kg over 4 h].
		Omega3/Omega6/PUFA	8 h fast for solids; 2 h fast plus 200 mL oral intake of water only; intraoperative infusion of ω-3 PUFA [0.2mcg/kg over 4 h].
**173**	Feguri-2019	carbohydrates	Brief fasting with oral intake of 200 mL of water with 25g (12.5%) of maltodextrin two hours before surgery.
		Omega3/	Brief fasting with 200 mL of water and intraoperative infusion of 0.2g/kg ω-3 PUFA for four hours.
		Omega3/Omega6/PUFA + carbohydrates	Brief fasting with 200mL of water with 25g (12.5%) of maltodextrin two hours before surgery and intraoperative infusion of 0.2g/kg of ω-3 PUFA for four hours.
		Placebo	Brief fasting with 200mL of water two hours before surgery.
**258**	Gomez Polo-2017	Corticosteroids	Methylprednisolone, 500 mg, IV, after induction of anaesthesia and dexamethasone, 4mg, every 8 hours during 24 hours after surgery.
		Placebo	Matching placebo same dose, at the same times
**163**	Halonen-2007	Corticosteroids	Hydrocortisone (100mg per 2mL of hydrocortisone sodium succinate in 100mL of 0.9% sodium chloride solution). the first dose in the evening of the operative day, then 1 dose every 8 hours during the next 3 days.
		Placebo	Matching placebo same dose, at the same times
**161**	Halvorsen-2003	Corticosteroids	Dexamethasone 8mg IV
		Placebo	Matching placebo same dose, at the same times
**159**	Heidarsdottir-2010	Omega3/Omega6/PUFA	N-3 PUFA, in two soft capsules, twice daily. 5–7 days prior to surgery and postoperatively until hospital discharge.
		Placebo	Olive oil. 2g twice daily
**158**	Heidt-2009	Omega3/Omega6/PUFA	Fish oil 100mg/kg body weight/day from the time of admission to hospital until transfer to a normal ward.
		Placebo	Soya oil, 100mg/kg body weight/day
**156**	Horbach-2011	NSAIDs	Naproxen, 275mg every 12 hours for 5 days postoperatively
		Placebo	Placebo, 275mg every 12 hours for 5 days postoperatively
**154**	Jacob-2015	Corticosteroids	Dexamethasone,1mg/kg with a maximum of 100 mg as single dose before CPB

		Placebo	Matching placebo same dose, at the same times
**152**	Ji-2009	Statin	Atorvastatin 20mg/day, started 1 week before the scheduled surgery
		Placebo	Usual care
**151**	Joss-2017	Omega3/Omega6/PUFA	PUFA, 1g twice daily, five days before surgery or within 24 hours after surgery until four weeks postoperatively.
		Placebo	Matching placebo same dose, at the same times
**146**	Kazemi-2013	N-acetyl-Cysteine	NAC, 1,200mg PO from 48 hours before and up to 72 hours after surgery
		Placebo	Matching placebo same dose, at the same times
**259**	Kilger-2011	Corticosteroids	Hydrocortisone 100mg IV for 10 minutes before induction of anaesthesia, followed by infusion of 10mg/h for 24 hours, which was reduced to 5mg/h on POD 2, then tapered to 3 × 20mg IV on POD 3 and 3 × 10mg IV on POD 4.
		Placebo	Matching placebo same dose, at the same times
**275**	Kim-2011	N-acetyl-Cysteine	NAC, 100mg/kg, IV, over a15 minute period immediately after aesthetic induction, followed by an IV at 40 mg/kg/day for 24 h.
		Placebo	100–200mg of fish oil/kg
**271**	Kolesnikov-2015	Omega3/Omega6/PUFA	Fish oil ,100–200mg/kg, IV
		No treatment	Usual care
**140**	Kourliouros-2011	Statin	Atorvastatin, 80mg, 7 days before surgery, and continued for 14 days after surgery or until discharge (whichever first).
		Statin	atorvastatin 10mg 7 days before surgery. Study medication was continued for 14 days after surgery or until discharge (whichever first).
**132**	Lomivorotov-2014	Omega3/Omega6/PUFA	Omega3 PUFA,200mg/kg/day before anaesthesia induction for 24 hours followed by 100mg/kg/day from POD 2 to POD 7 Doses of 2mL/kg/day of lipids at the first day and 1mL/kg/day of lipids from POD 2 to 7.
		Placebo	Matching placebo same dose, at the same times
**260**	Lomivorotov-2012	Corticosteroids	Methylprednisolone 20mg/kg intraoperatively immediately after anaesthesia induction
		Placebo	Matching placebo same dose, at the same times
**267**	mannacio-2008	Statin	Rosuvastatin 20mg/d starting 7 days before the planned operation, regardless of cholesterol level.
		Placebo	Matching placebo same dose, at the same times
**22**	Mansour-2016	Statin	Atorvastatin 40mg/day one week before surgery and continued postoperatively
		No treatment	Usual care
**261**	Mardani-2013	Corticosteroids	Dexamethasone ,8mg IV, immediately before surgery and followed by 8mg (2 mL) every 8 h for the first 3 postoperative days.
		Placebo	Matching placebo same dose, at the same times
**25**	Mirhosseini-2010	Corticosteroids	Methylprednisolone, 5mg/kg, IV after induction of anaesthesia
		Placebo	Matching placebo same dose, at the same times

**128**	Mirmohammadsadeghi-2018	Ascorbic acid	Vitamin C, 2g, IV, 24hours preoperatively, 500mg, IV, every 12 hours in ICU, and 500 mg every 12 hours postoperatively for 48 hours in ward.
		No treatment	Usual care
**126**	Moludi-2016	Ascorbic acid	Vitamin C, 2g/d, IV, before surgery followed by 1 g/d 4 days postoperatively
		Placebo	Matching placebo same dose, at the same times
**276**	Orhan-2006	N-acetyl-Cysteine	NAC IV intraoperatively
		No treatment	Usual care
**111**	Ozaydin-2008	N-acetyl-Cysteine	NAC 50mg/kg, IV preoperatively, followed by IV infusion for 48 h after the operation at a dose of 50 mg/kg/day.
		Placebo	Matching placebo same dose, at the same times
**110**	Papoulidis-2010	Ascorbic acid	Vitamin C IV, 500mg twice a day from the first postoperative day and for the next five days.
		Placebo	Matching placebo same dose, at the same times
**108**	Patti-2006	Statin	Atorvastatin (40 mg/d) 7 days before surgery until discharge
		Placebo	Matching placebo same dose, at the same times
**106**	Pierri-2016	Statin	Atorvastatin 40mg daily for 7 days before surgery, stopped the evening before surgery and resumed 24 h later.
		Statin	Atorvastatin 80mg daily for 7 days before surgery, stopped the evening before surgery and resumed 24 h later.
**105**	Prasongsukarn-2005	Corticosteroids	Methylprednisolone, 1g, IV, before CPB and dexamethasone 4mg, IV, every 6 hours for a total of 4 doses in the first 24 hours postoperatively.
		Placebo	Matching placebo same dose, at the same times
**102**	Rodrigo-2013	Ascorbic acid + Omega3/Omega6/PUFA + Other (vit E)	N-3 PUFAs, 2g/day preoperatively and vitamin C (1g/day) plus vitamin E (400IU/day) before surgery. Continued until hospital discharge.
		Placebo	Matching placebo same dose, at the same times
**32**	Rubanenko-2015	Statin	Atorvastatin, 25.3±11.7mg/day
		No treatment	Usual care
**99**	Rubens-2005	Corticosteroids	Methylprednisolone (1 g IV) at the time of insertion of the central line and before the incision.
		Placebo	Matching placebo same dose, at the same times
**294**	Sadeghpour-2014	Ascorbic acid	Vitamin C, 2g, IV, before surgery and 1g daily 4days postoperatively
		Placebo	Placebo in the same shape and size as intervention
**33**	Samadikhan-2014	Statin + Ascorbic acid	Atorvastatin 40mg (daily) + oral Vitamin C (2g in operation day and 1g from second day until 5 day)
		Statin + Placebo	Atorvastatin 40mg + placebo
**95**	Saravanan-2016	Omega3/Omega6/PUFA	N-3 PUFA, 1.8g/day, PO, 14 days

		Placebo	Olive oil, for at least 5 days prior to the surgery
**96**	Saravanan-2010	Omega3/Omega6/PUFA	N-3 PUFA, 2 g/d, 5 days before surgery
		Placebo	Olive oil 2g/d, identical as intervention
**94**	Sarzaeem-2014	Ascorbic acid	Vitamin C, 7g, parenteral, 12 hours before surgery and 5 days after surgery
		Placebo	Matching placebo same dose, at the same times). same as intervention.
**295**	Sarzaeem-2014	Colchicine	Colchicine 1mg, PO, preoperatively, plus 0.5mg maintenance dose postoperative day 1 to 5.
		Placebo	Matching placebo same dose, at the same times
**262**	Schurr-2001	Corticosteroids	Methylprednisolone ,10mg/kg, IV, 4 hours preoperatively
		No treatment	Usual care
**263**	Sobieski-2008	Corticosteroids	Dexamethasone, 100mg, IV, intra operatively
		Placebo	Sterile saline, 10ml
**84**	Soleimani-2018	N-acetyl-Cysteine	NAC, 50mg/kg, IV diluted to a total volume of 50mL of normal saline, over a period of 30 min. once before and two doses after surgery.
		Placebo	Matching placebo same dose, at the same times
**82**	Song-2008	Statin	Atorvastatin, 20mg, daily, 3 days preoperatively PO, 1 to 2 days postoperatively NG
		Statin	Atorvastatin, 20mg, PO, after discharge for 30days after 30 days
**39**	Sorice-2011	Omega3/Omega6/PUFA	EPA/DHA, 850 to 882mg, PO, daily, 5days preoperatively and until discharge
		No treatment	Usual care
**40**	Stanger-2014	Omega3/Omega6/PUFA	Omegaven, 0.5ml/kg, IV, preoperatively and postoperatively
		Ascorbic acid	Ascorbic acid, 500mg, intra operatively and postoperatively
		Ascorbic acid + Omega3/Omega6/PUFA	Omegaven, 0.5ml/kg, IV, pre operatively and postoperatively and Ascorbic acid, 500mg, intra operatively and postoperatively.
		No treatment	Usual care
**264**	Suezawa-2013	Corticosteroids	Methylprednisolone, 1000mg, IV, during anaesthesia induction
		Placebo	Matching placebo same dose, at the same times
**80**	Sun-2011	Statin	Atorvastatin, 20mg, PO, from7days pre operatively
		Placebo	Matching placebo same dose, at the same times
**41**	Tabbalat-2020	Colchicine	Colchicine, 1mg/kg, 12 to 24 hours prior to surgery, followed by colchicine (0.5mg) immediately after surgery (via a nasogastric tube) and daily treatment at this dose until hospital discharge.
		Placebo	Matching placebo same dose, at the same times
**42**	Tabbalat-2015	Colchicine	Colchicine 2mg, NG, 12-24 hours prior to surgery and 1mg 4 hours before or immediately after surgery, continued at a dose of 0.5 mg twice daily until hospital discharge.
		No treatment	Usual care

**265**	Vukovic-2010	Statin	Atorvastatin, 20mg/kg, during the 3 weeks before surgery
		Corticosteroids	Methylprednisolone, 10mg/kg, IV, after induction of anaesthesia
		No treatment	Usual care
**65**	Wang-2016	Amiodarone	Amiodarone, 1mg/kg, poly-based hydrogel spray
		Corticosteroids	Triamcinolone acetonide 0.5mg/kg, poly-based hydrogel spray
		Placebo	Hydrogels spray
**67**	Wang-2020	Ascorbic acid	Vitamin C, 1 g (diluted to 10mL), IV with a total of 3g
		Placebo	Normal saline, 10ml, IV
**64**	Weis-2009	Corticosteroids	Hydrocortisone (100 mg IV during 10 minutes) before anaesthesia and followed by a continuous infusion of 10 mg/hr for 24 hours, which was tapered in postoperative day 1 to day 4.
		Placebo	Matching placebo same dose, at the same times
**277**	Wijeysundera-2007	N-acetyl-Cysteine	NAC 100mg/kg, IV, over 30 min after induction of anaesthesia. followed by a 20mg/kg/hr infusion until four hours after CPB.
		placebo	Matching placebo same dose, at the same times
**47**	Wilbring-2014	Omega3/Omega6/PUFA	Omega-3 PUFA, 2g daily, PO, 5 days before surgery and postoperative course
		No treatment	Usual care
**296**	Yamamoto-2014	Omega3/Omega6/PUFA	EPA, 1800mg/day, PO
		No treatment	Usual care
**59**	Yared-2000	Corticosteroids	Dexamethasone 0.6mg/kg, IV
		Placebo	Matching placebo same dose, at the same times
**60**	Yared-2007	Corticosteroids	Dexamethasone 0.6mg/kg IV
		Placebo	Matching placebo same dose, at the same times
**53**	Zarpelon-2016	Colchicine	Colchicine, 1mg, PO, twice daily, initiated 24 hours before surgery. Colchicine 0.5mg, PO, twice daily until hospital discharge.
		No treatment	Usual care
**266**	Zheng-2016	Statin	Rosuvastatin 20mg, PO, daily, 8 days before surgery and for 5 days thereafter
		Placebo	Matching placebo same dose, at the same times
**323**	Talasaz-2021	Vitamin D3	Vitamin D3, 600,000IU in three divided doses throughout the day, PO, 5days before surgery.
		Control	Usual care
**325**	Samadifar-2023	Atorvastatin	Atorvastatin, 40mg, PO, daily, 1 week before surgery
		Rosuvastatin	Rosuvastatin, 20 mg, PO, daily, 1 week before surgery
**326**	Shvartz-2022	Colchicine	Colchicine, 1mg, PO, 24 hours before surgery and day 2,3,4 and 5 in the postoperative period.

		Placebo	Matching placebo same dose, at the same times
**327**	Shvartz-2022	Colchicine	Colchicine, 1mg, PO, 24 hours before surgery and day 2,3,4 and 5 in the postoperative period.
		Placebo	Matching placebo same dose, at the same times

Footnote; NA; not applicable, IV; intravenous, g; gram, mg; milligram, mcg; microgram, kg; kilogram, L; Liter, mL; millilitre, mEq; milliequivalent, h; hour, min; minute, IU; international unit, CPB; cardiopulmonary bypass, POD; postoperative day, NSAID; Non-steroidal anti-inflammatory drug, PUFA; Polyunsaturated fatty acid, NAC; N-acetyl-Cysteine, EPA; Eicosapentaenoic acid, DHA; docosahexaenoic acid, ICU; intensive care unit

Supplemental Figure S1; Traffic light plots for ROB 2.0 risk of Bias assessment

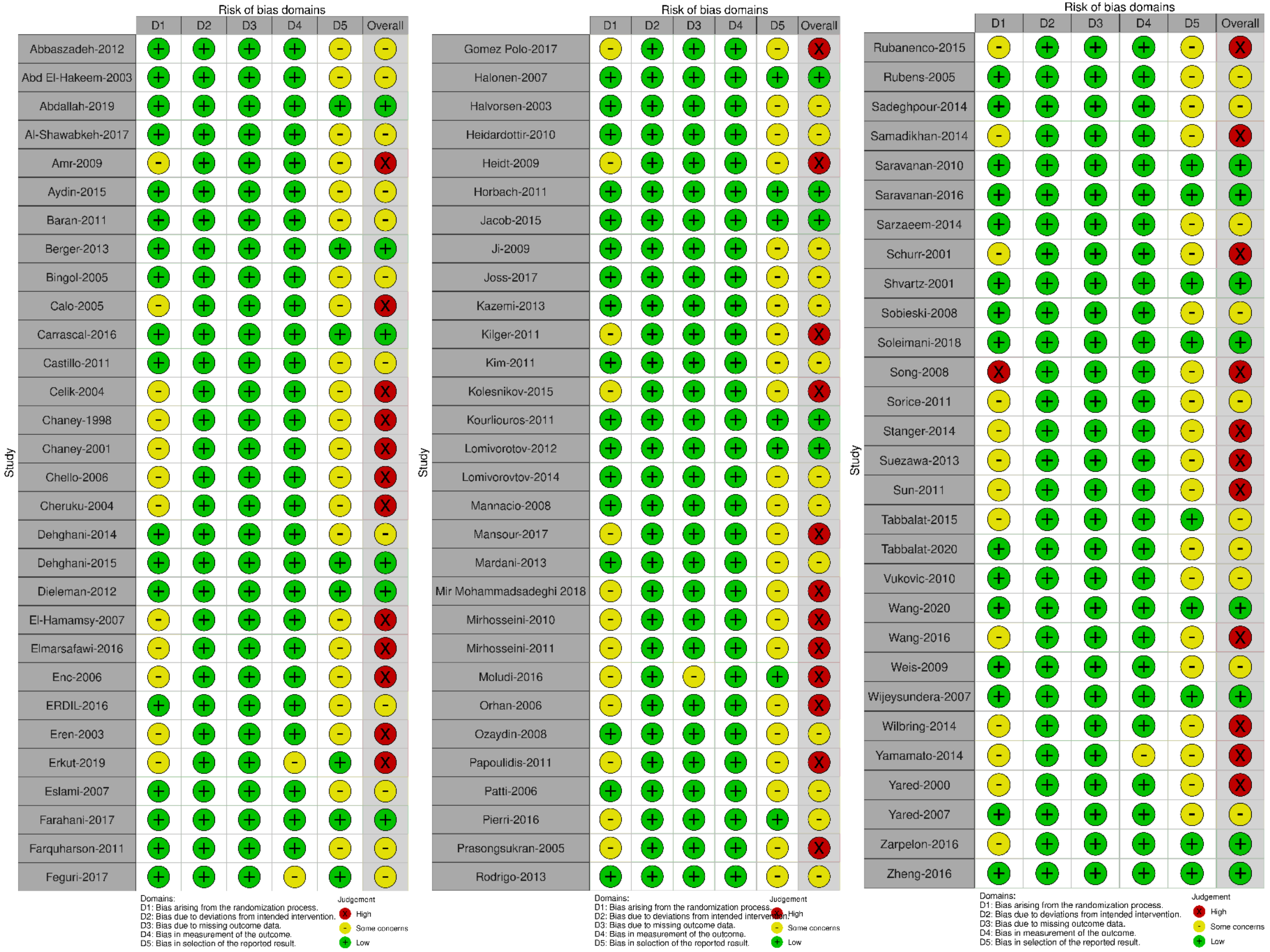

Supplemental Figure S2; Overall risk of Bias assessment for ROB 2.0 domains

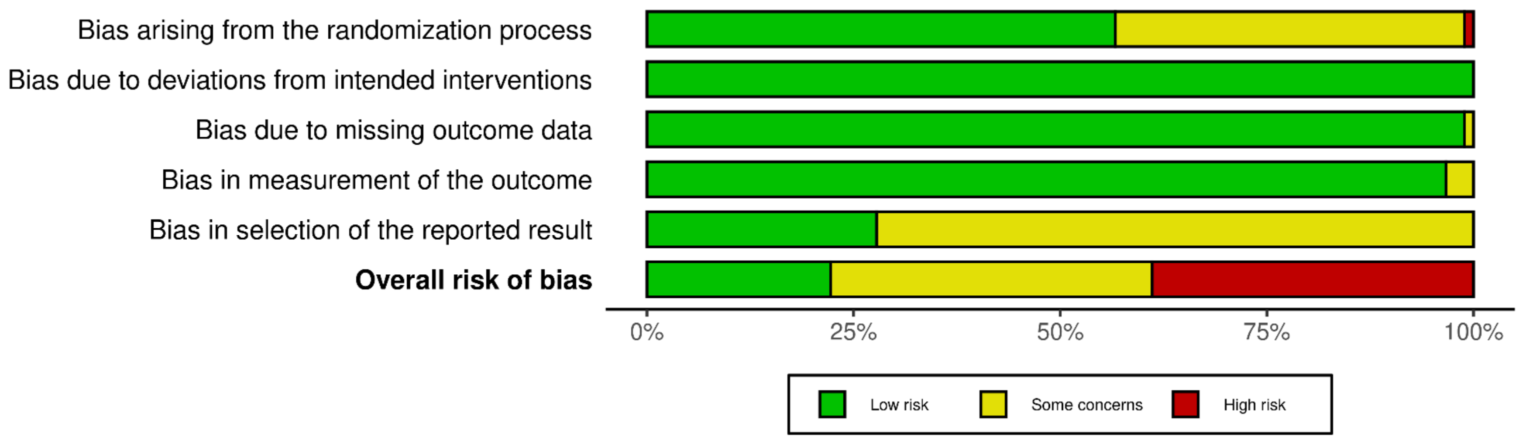

Supplemental Figure S3; Network map of the treatments for all-cause mortality

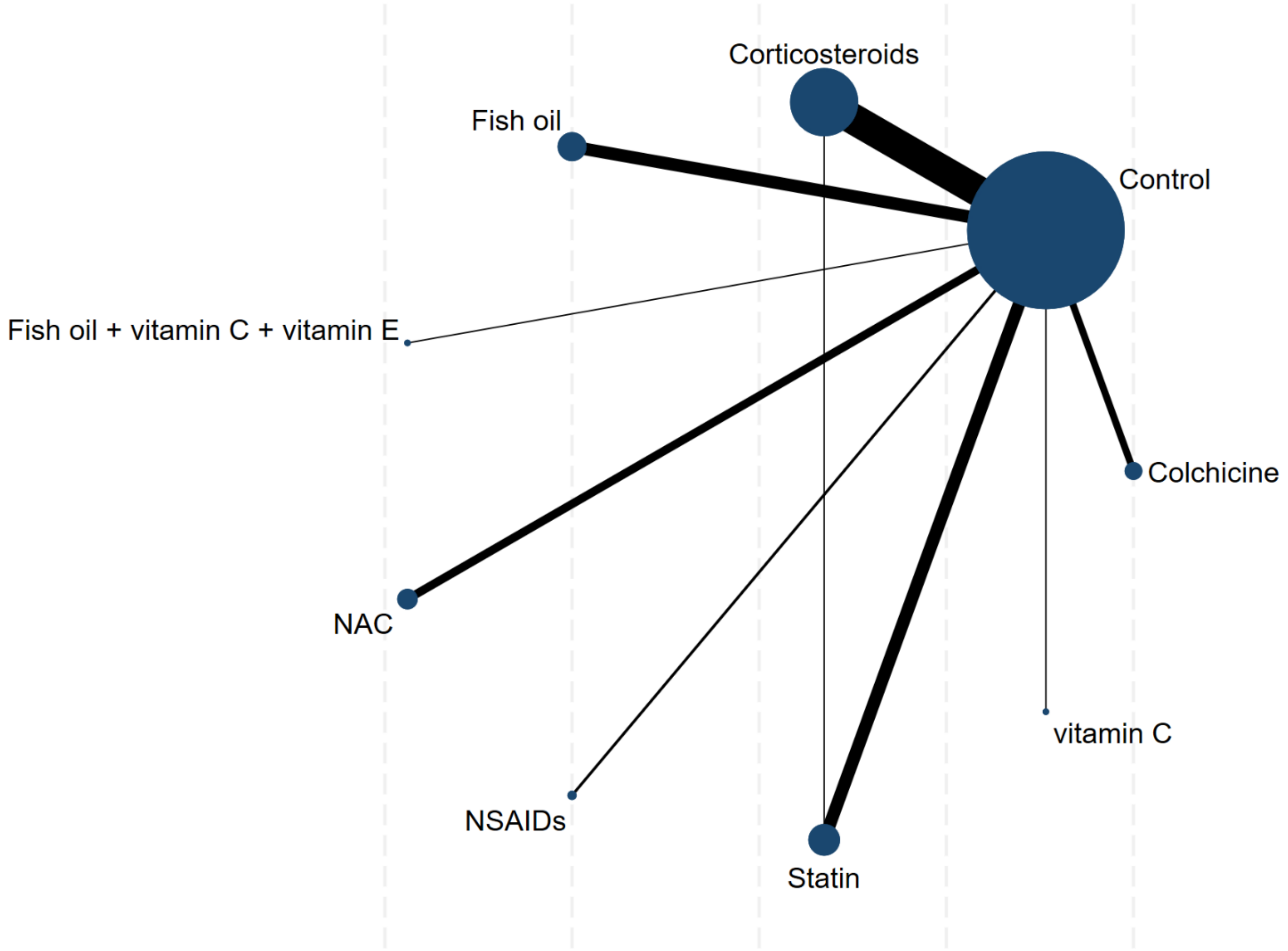

The size of the node (circle) corresponds to the number of patients randomized to that intervention. The thickness of the lines corresponds to the number of studies for each comparison.

Supplemental Figure S4; Network map of the treatments for serious adverse events

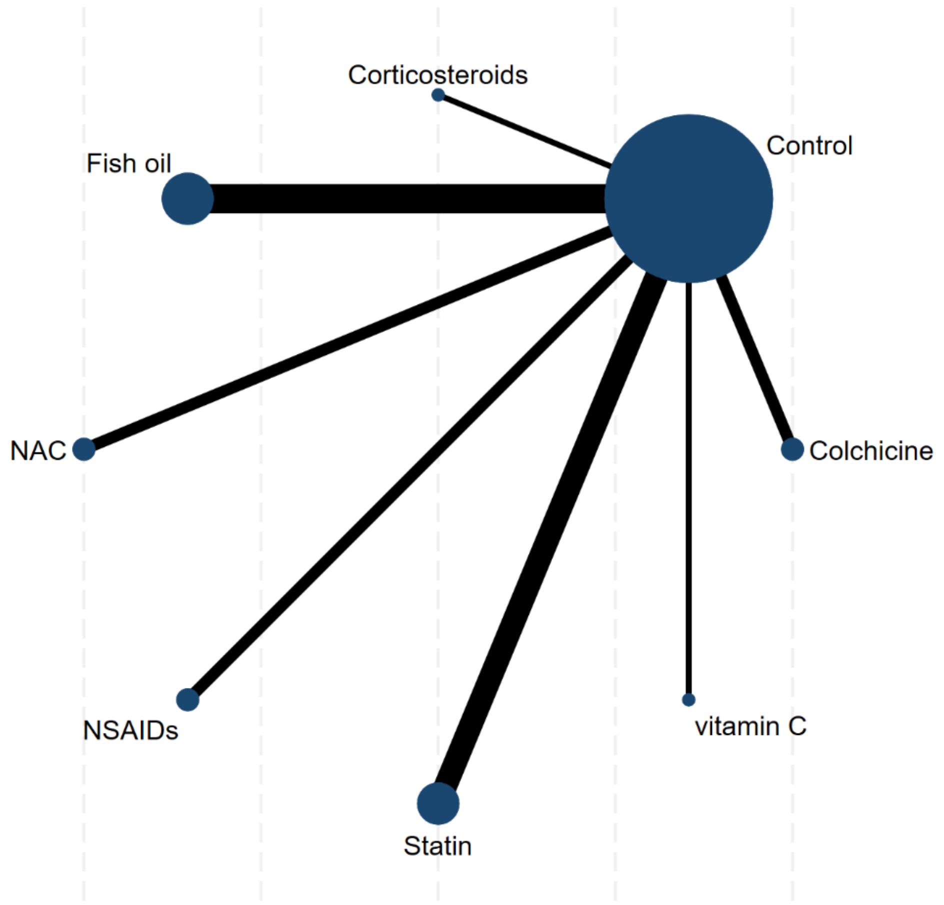

The size of the node (circle) corresponds to the number of patients randomized to that intervention. The thickness of the lines corresponds to the number of studies for each comparison.

Supplemental Figure S5; Network map of the treatments for duration of hospitalization

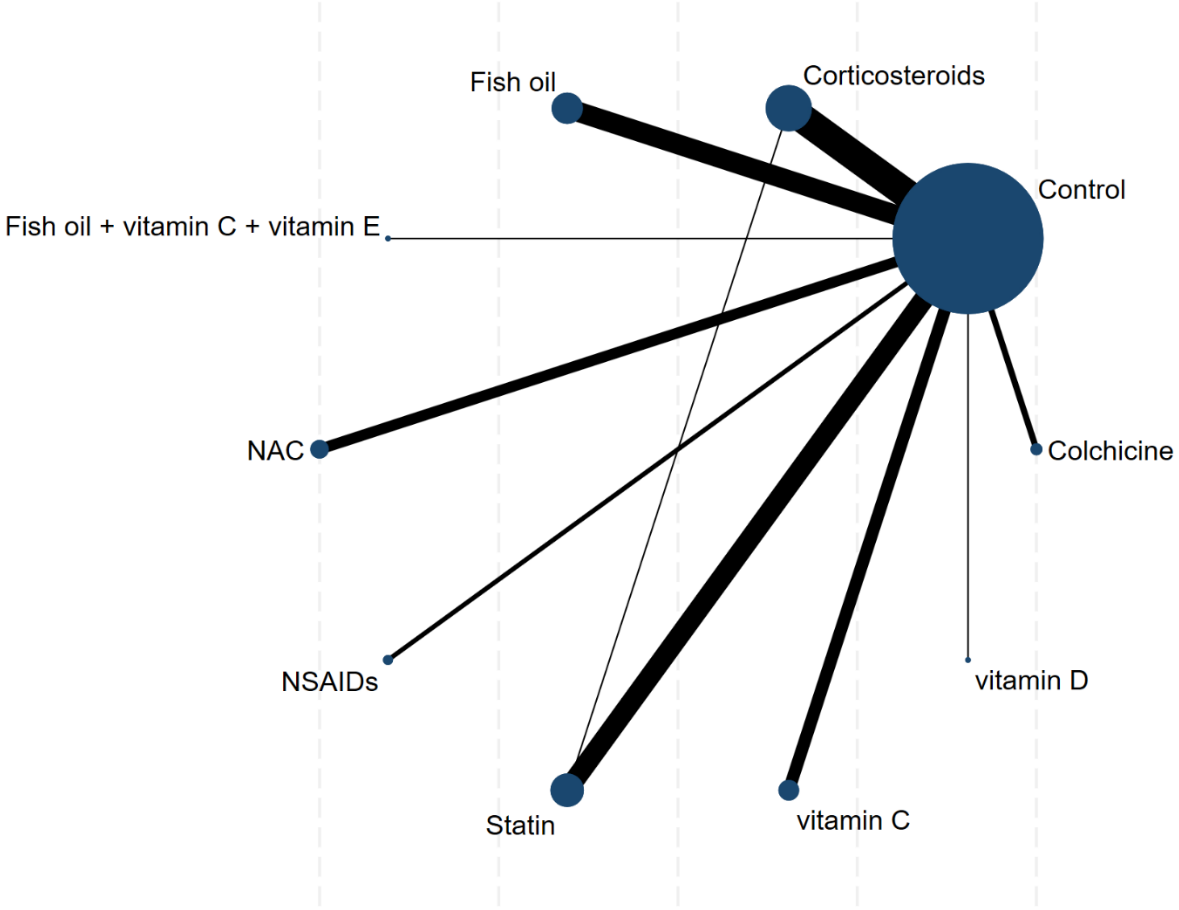

The size of the node (circle) corresponds to the number of patients randomized to that intervention. The thickness of the lines corresponds to the number of studies for each comparison.

Supplemental Figure S6; Indirect estimates for incidence of postoperative atrial fibrillation

Comparison	Number of Studies	Direct Evidence	I2	RR	95%-CI
Fish oil vs Control					
Direct estimate	17	1.00	50%	0.79	[0.66; 0.95]
Indirect estimate				0.30	[0.02; 4.27]
Network estimate				0.79	[0.65; 0.95]
Fish oil + vitamin C vs Control					
Direct estimate	1	0.67		1.37	[0.51; 3.73]
Indirect estimate				1.09	[0.26; 4.60]
Network estimate				1.27	[0.56; 2.90]
vitamin C vs Control					
Direct estimate	9	0.99	37%	0.64	[0.50; 0.82]
Indirect estimate				1.15	[0.10; 13.17]
Network estimate				0.64	[0.50; 0.82]
Corticosteroids vs Statin					
Direct estimate	1	0.03		0.75	[0.17; 3.31]
Indirect estimate				1.26	[0.96; 1.66]
Network estimate				1.24	[0.95; 1.62]
Fish oil vs Fish oil + vitamin C					
Direct estimate	1	0.53		0.51	[0.16; 1.60]
Indirect estimate				0.76	[0.23; 2.55]
Network estimate				0.62	[0.27; 1.42]
Fish oil vs vitamin C					
Direct estimate	1	0.06		0.80	[0.23; 2.78]
Indirect estimate				1.26	[0.92; 1.74]
Network estimate				1.23	[0.90; 1.67]
Fish oil + vitamin C vs vitamin C					
Direct estimate	1	0.62		1.56	[0.54; 4.50]
Indirect estimate				2.93	[0.76; 11.36]
Network estimate				1.99	[0.86; 4.57]

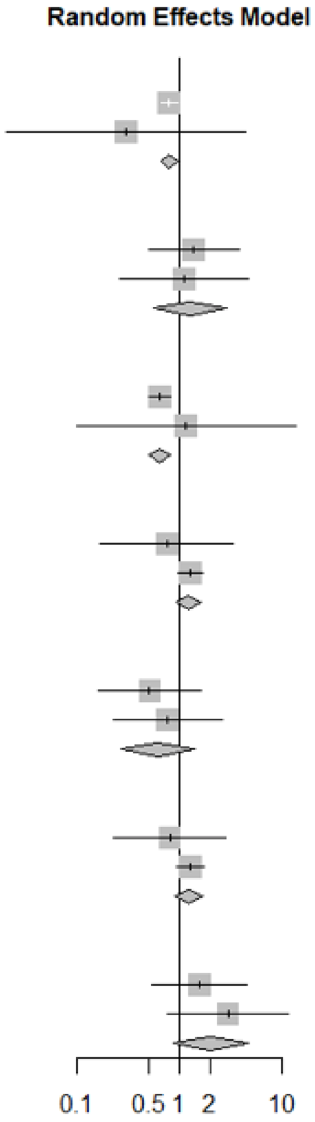

Supplemental Figure S7; Indirect estimates for incidence of postoperative all-cause mortality

Comparison	Number of Studies	Direct Evidence	I2	RR	95%-CI
**Corticosteroids vs Control**					
Direct estimate	22	1.00	0	0.77	[0.54; 1.09]
Indirect estimate				1.35	[0.00; 1439.54]
Network estimate				0.77	[0.54; 1.09]
**Statin vs Control**					
Direct estimate	9	0.98	0	1.13	[0.47; 2.72]
Indirect estimate				0.61	[0.00; 527.58]
Network estimate				1.12	[0.47; 2.67]
**Corticosteroids vs Statin**					
Direct estimate	1	0.06		1.00	[0.02; 48.74]
Indirect estimate				0.67	[0.26; 1.75]
Network estimate				0.68	[0.27; 1.74]

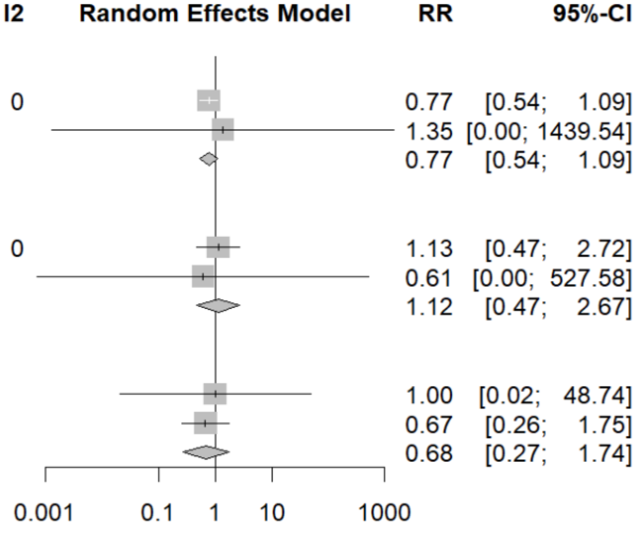

Supplemental Figure S8; Indirect estimates for duration of hospitalization

Comparison	Number of Studies	Direct Evidence	I2	MD	95%−CI
**Colchicine:Control**					
Direct estimate	4	1.00	87%	−0.29	[−1.91; 1.34]
Indirect estimate					
Network estimate				−0.29	[−1.91; 1.34]
**Colchicine:Corticosteroids**					
Direct estimate	0	0			
Indirect estimate				0.46	[−1.33; 2.25]
Network estimate				0.46	[−1.33; 2.25]
**Colchicine:Fish oil**					
Direct estimate	0	0			
Indirect estimate				0.81	[−1.10; 2.72]
Network estimate				0.81	[−1.10; 2.72]
**Colchicine:Fish oil + vitamin C + vitamin E**					
Direct estimate	0	0			
Indirect estimate				0.51	[−2.79; 3.82]
Network estimate				0.51	[−2.79; 3.82]
**Colchicine:NAC**					
Direct estimate	0	0			
Indirect estimate				−0.22	[−2.24; 1.79]
Network estimate				−0.22	[−2.24; 1.79]
**Colchicine:NSAIDs**					
Direct estimate	0	0			
Indirect estimate				−2.15	[−4.61; 0.31]
Network estimate				−2.15	[−4.61; 0.31]
**Colchicine:Statin**					
Direct estimate	0	0			
Indirect estimate				0.17	[−1.67; 2.02]
Network estimate				0.17	[−1.67; 2.02]

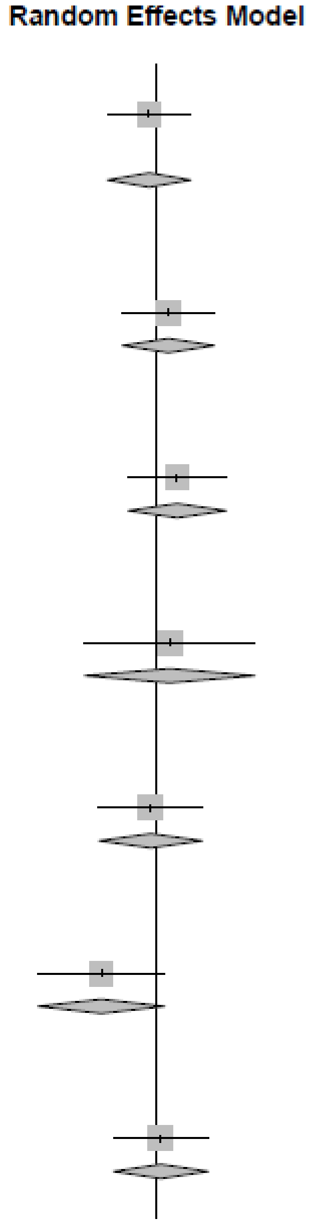

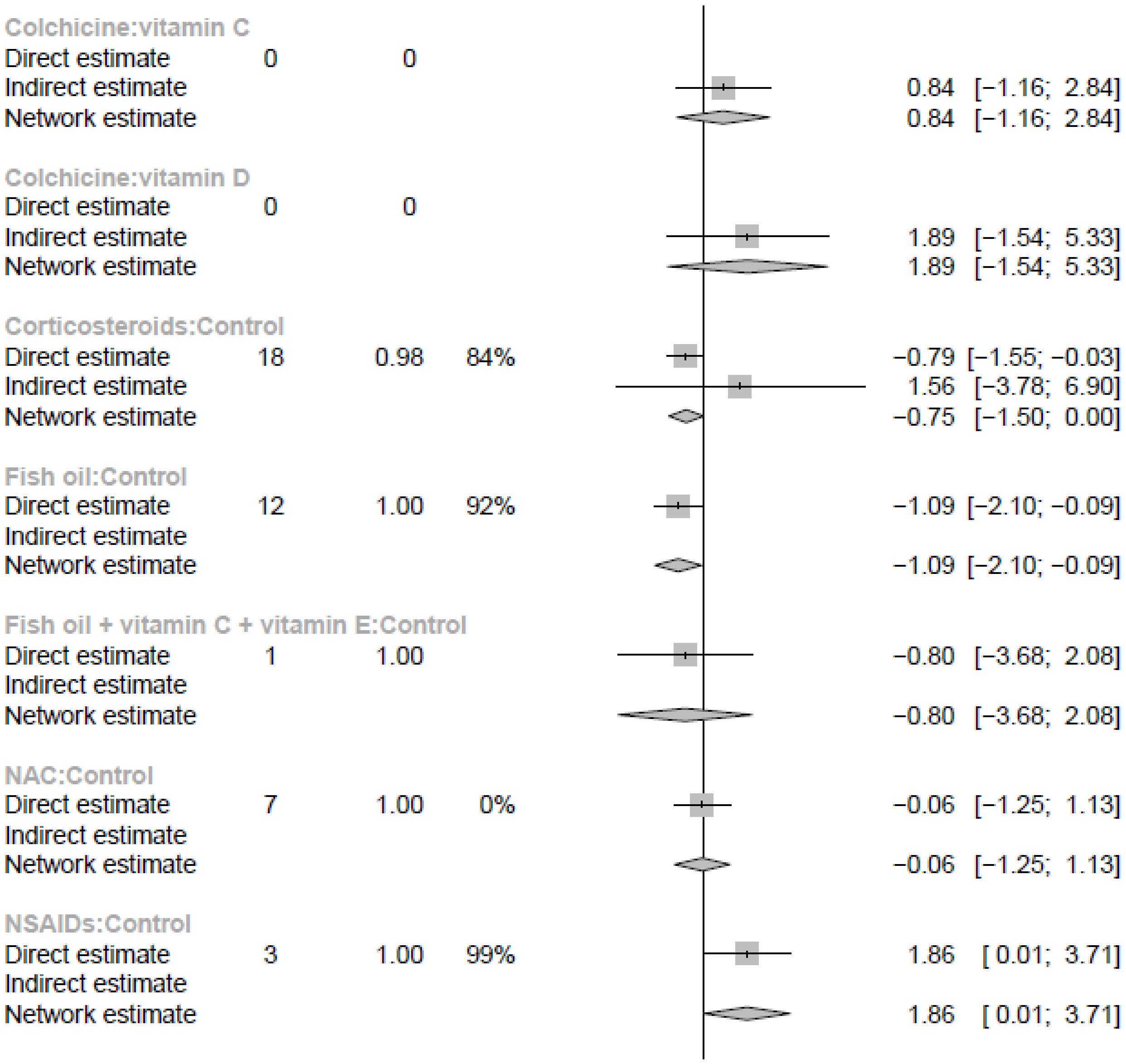
Colchicine:vitamin C
Direct estimate 0 0
Indirect estimate 0.84 [−1.16; 2.84]
Network estimate 0.84 [−1.16; 2.84]
Colchicine:vitamin D
Direct estimate 0 0
Indirect estimate 1.89 [−1.54; 5.33]
Network estimate 1.89 [−1.54; 5.33]
Corticosteroids:Control
Direct estimate 18 0.98 84% −0.79 [−1.55; −0.03]
Indirect estimate 1.56 [−3.78; 6.90]
Network estimate −0.75 [−1.50; 0.00]
Fish oil:Control
Direct estimate 12 1.00 92% −1.09 [−2.10; −0.09]
Indirect estimate
Network estimate −1.09 [−2.10; −0.09]
Fish oil + vitamin C + vitamin E:Control
Direct estimate 1 1.00 −0.80 [−3.68; 2.08]
Indirect estimate
Network estimate −0.80 [−3.68; 2.08]
NAC:Control
Direct estimate 7 1.00 0% −0.06 [−1.25; 1.13]
Indirect estimate
Network estimate −0.06 [−1.25; 1.13]
NSAIDs:Control
Direct estimate 3 1.00 99% 1.86 [0.01; 3.71]
Indirect estimate
Network estimate 1.86 [0.01; 3.71]

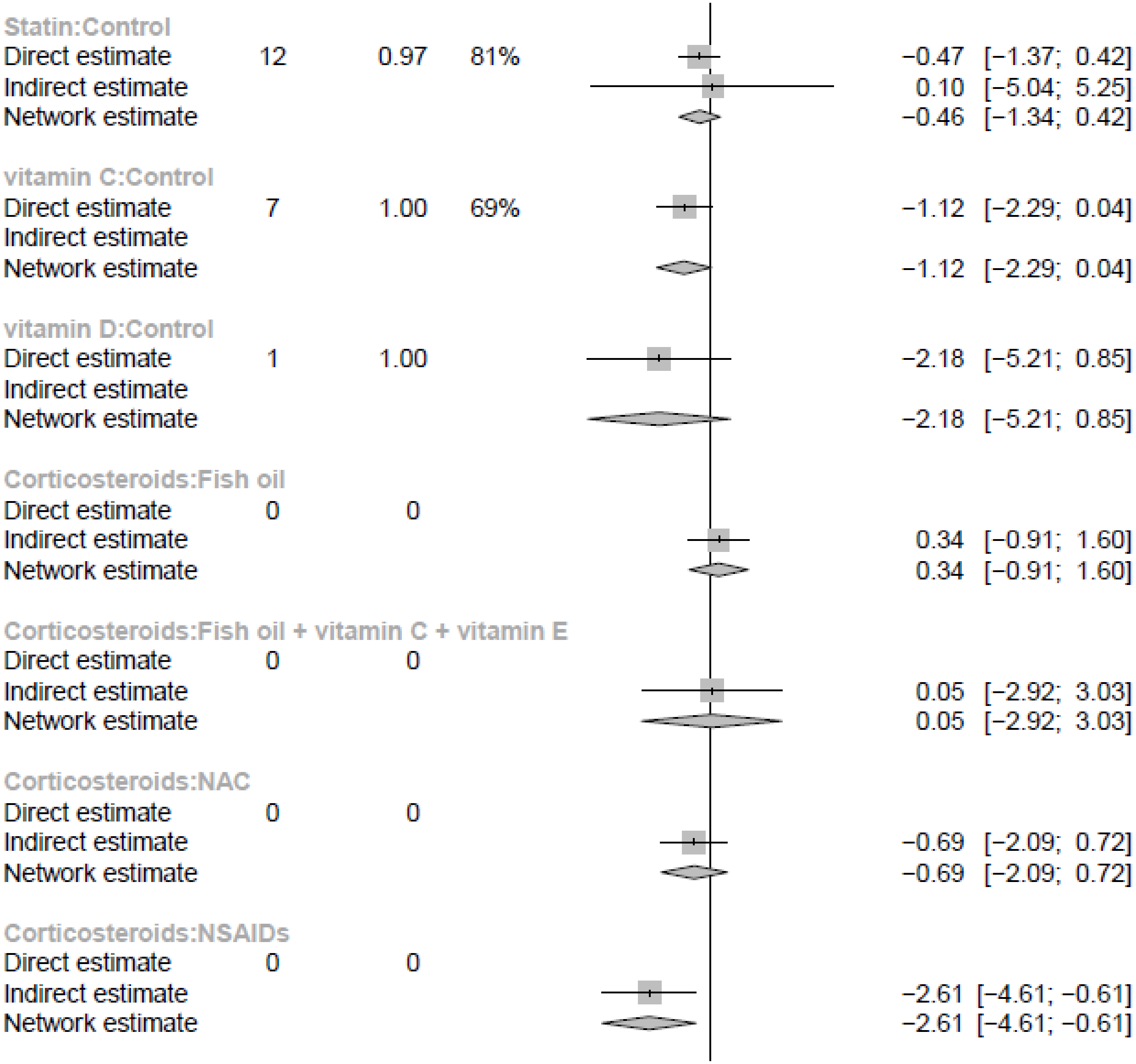
Statin:Control
Direct estimate 12 0.97 81% −0.47 [−1.37; 0.42]
Indirect estimate 0.10 [−5.04; 5.25]
Network estimate −0.46 [−1.34; 0.42]
vitamin C:Control
Direct estimate 7 1.00 69% −1.12 [−2.29; 0.04]
Indirect estimate
Network estimate −1.12 [−2.29; 0.04]
vitamin D:Control
Direct estimate 1 1.00 −2.18 [−5.21; 0.85]
Indirect estimate
Network estimate −2.18 [−5.21; 0.85]
Corticosteroids:Fish oil
Direct estimate 0 0
Indirect estimate 0.34 [−0.91; 1.60]
Network estimate 0.34 [−0.91; 1.60]
Corticosteroids:Fish oil + vitamin C + vitamin E
Direct estimate 0 0
Indirect estimate 0.05 [−2.92; 3.03]
Network estimate 0.05 [−2.92; 3.03]
Corticosteroids:NAC
Direct estimate 0 0
Indirect estimate −0.69 [−2.09; 0.72]
Network estimate −0.69 [−2.09; 0.72]
Corticosteroids:NSAIDs
Direct estimate 0 0
Indirect estimate −2.61 [−4.61; −0.61]
Network estimate −2.61 [−4.61; −0.61]

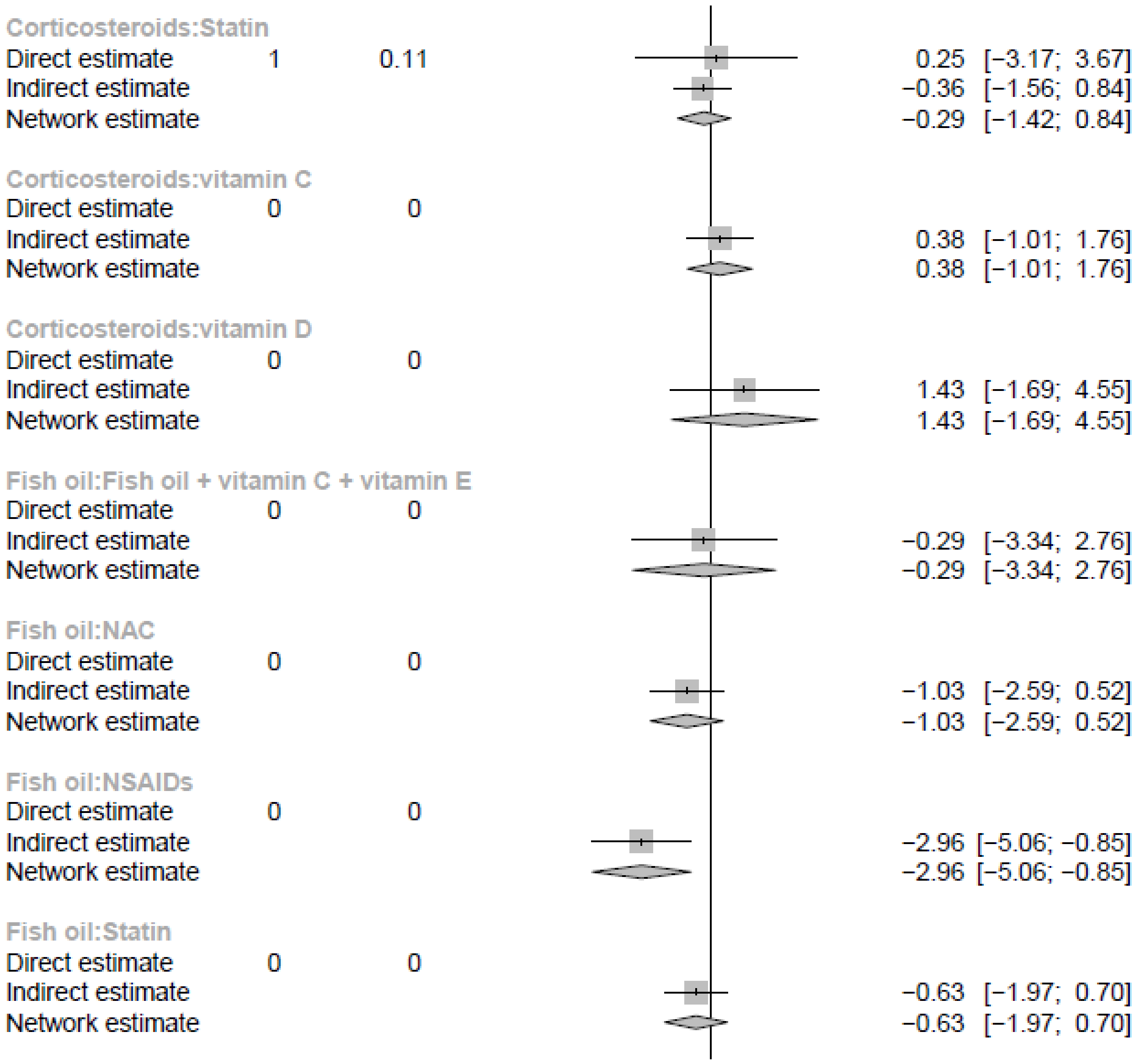
Corticosteroids:Statin
Direct estimate 1 0.11 0.25 [−3.17; 3.67]
Indirect estimate −0.36 [−1.56; 0.84]
Network estimate −0.29 [−1.42; 0.84]
Corticosteroids:vitamin C
Direct estimate 0 0
Indirect estimate 0.38 [−1.01; 1.76]
Network estimate 0.38 [−1.01; 1.76]
Corticosteroids:vitamin D
Direct estimate 0 0
Indirect estimate 1.43 [−1.69; 4.55]
Network estimate 1.43 [−1.69; 4.55]
Fish oil:Fish oil + vitamin C + vitamin E
Direct estimate 0 0
Indirect estimate −0.29 [−3.34; 2.76]
Network estimate −0.29 [−3.34; 2.76]
Fish oil:NAC
Direct estimate 0 0
Indirect estimate −1.03 [−2.59; 0.52]
Network estimate −1.03 [−2.59; 0.52]
Fish oil:NSAIDs
Direct estimate 0 0
Indirect estimate −2.96 [−5.06; −0.85]
Network estimate −2.96 [−5.06; −0.85]
Fish oil:Statin
Direct estimate 0 0
Indirect estimate −0.63 [−1.97; 0.70]
Network estimate −0.63 [−1.97; 0.70]

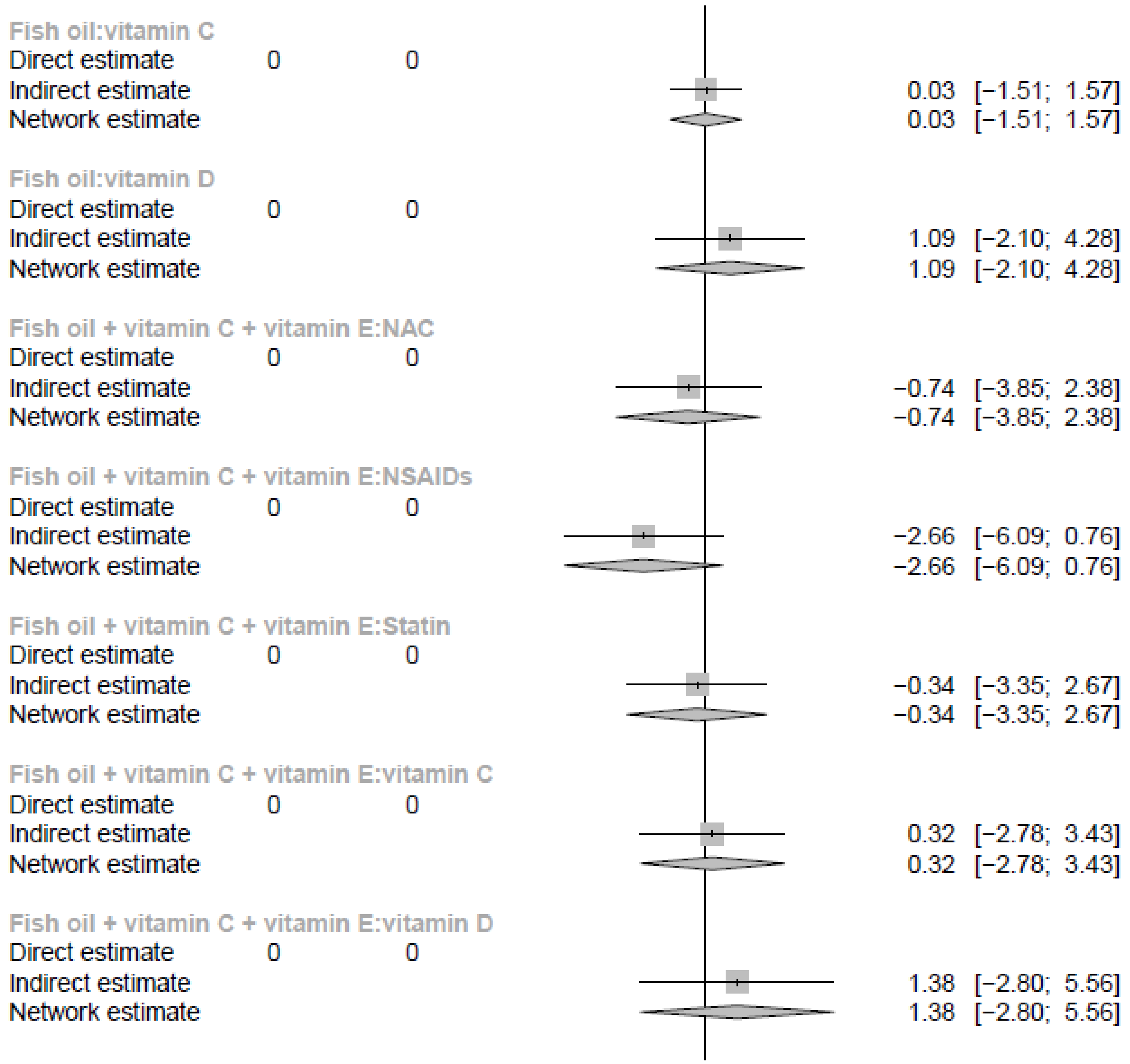
Fish oil:vitamin C
Direct estimate 0 0
Indirect estimate 0.03 [−1.51; 1.57]
Network estimate 0.03 [−1.51; 1.57]
Fish oil:vitamin D
Direct estimate 0 0
Indirect estimate 1.09 [−2.10; 4.28]
Network estimate 1.09 [−2.10; 4.28]
Fish oil + vitamin C + vitamin E:NAC
Direct estimate 0 0
Indirect estimate −0.74 [−3.85; 2.38]
Network estimate −0.74 [−3.85; 2.38]
Fish oil + vitamin C + vitamin E:NSAIDs
Direct estimate 0 0
Indirect estimate −2.66 [−6.09; 0.76]
Network estimate −2.66 [−6.09; 0.76]
Fish oil + vitamin C + vitamin E:Statin
Direct estimate 0 0
Indirect estimate −0.34 [−3.35; 2.67]
Network estimate −0.34 [−3.35; 2.67]
Fish oil + vitamin C + vitamin E:vitamin C
Direct estimate 0 0
Indirect estimate 0.32 [−2.78; 3.43]
Network estimate 0.32 [−2.78; 3.43]
Fish oil + vitamin C + vitamin E:vitamin D
Direct estimate 0 0
Indirect estimate 1.38 [−2.80; 5.56]
Network estimate 1.38 [−2.80; 5.56]

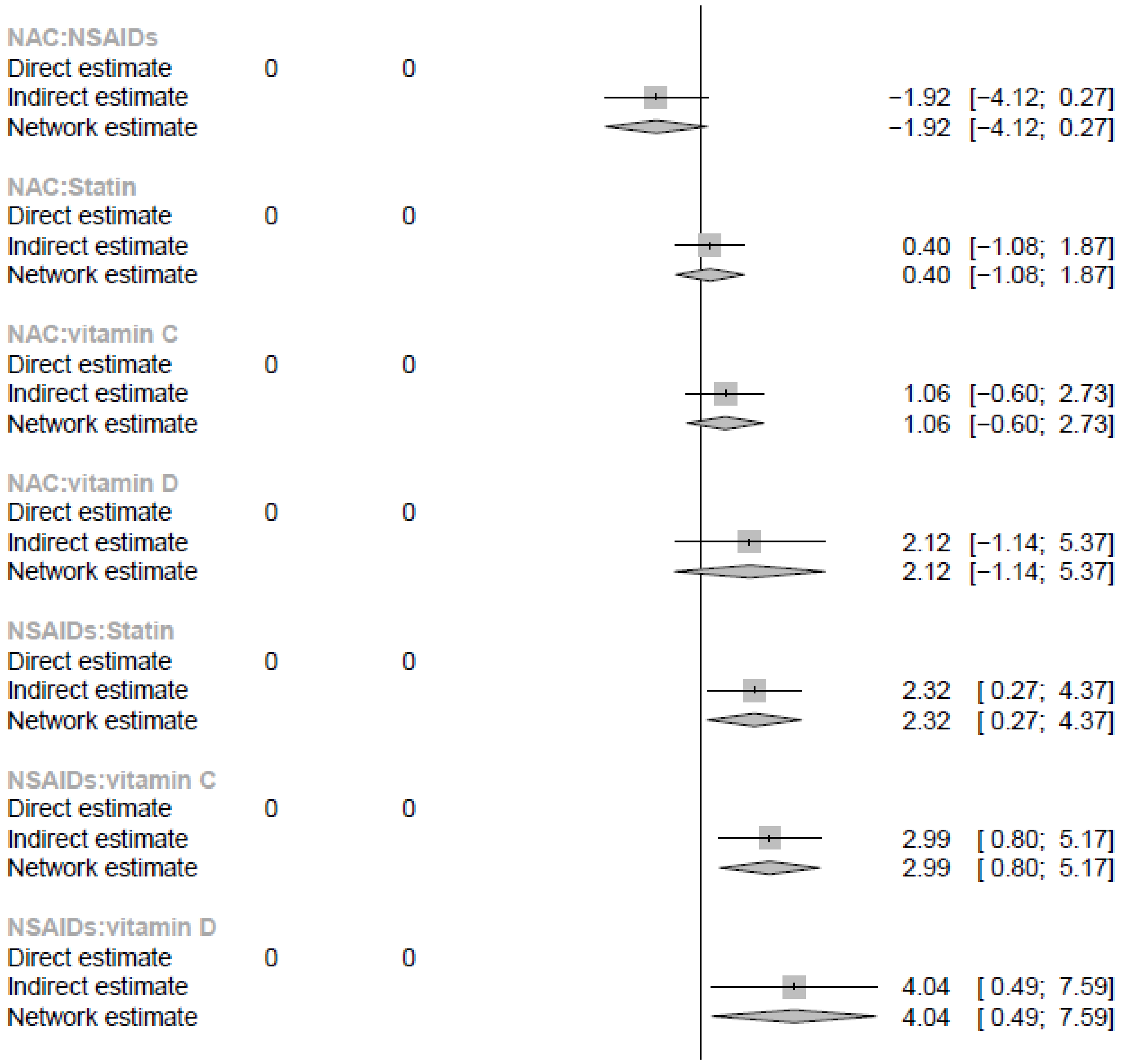
NAC:NSAIDs
Direct estimate 0 0
Indirect estimate −1.92 [−4.12; 0.27]
Network estimate −1.92 [−4.12; 0.27]
NAC:Statin
Direct estimate 0 0
Indirect estimate 0.40 [−1.08; 1.87]
Network estimate 0.40 [−1.08; 1.87]
NAC:vitamin C
Direct estimate 0 0
Indirect estimate 1.06 [−0.60; 2.73]
Network estimate 1.06 [−0.60; 2.73]
NAC:vitamin D
Direct estimate 0 0
Indirect estimate 2.12 [−1.14; 5.37]
Network estimate 2.12 [−1.14; 5.37]
NSAIDs:Statin
Direct estimate 0 0
Indirect estimate 2.32 [0.27; 4.37]
Network estimate 2.32 [0.27; 4.37]
NSAIDs:vitamin C
Direct estimate 0 0
Indirect estimate 2.99 [0.80; 5.17]
Network estimate 2.99 [0.80; 5.17]
NSAIDs:vitamin D
Direct estimate 0 0
Indirect estimate 4.04 [0.49; 7.59]
Network estimate 4.04 [0.49; 7.59]

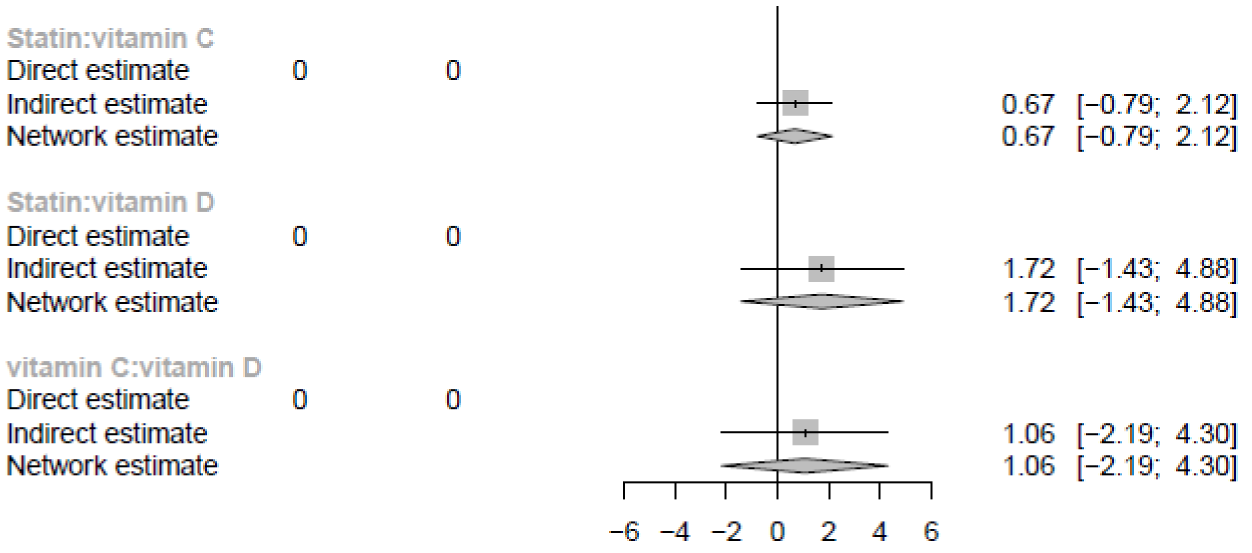
Statin:vitamin C
Direct estimate 0 0
Indirect estimate 0.67 [−0.79; 2.12]
Network estimate 0.67 [−0.79; 2.12]
Statin:vitamin D
Direct estimate 0 0
Indirect estimate 1.72 [−1.43; 4.88]
Network estimate 1.72 [−1.43; 4.88]
vitamin C:vitamin D
Direct estimate 0 0
Indirect estimate 1.06 [−2.19; 4.30]
Network estimate 1.06 [−2.19; 4.30]
−6 −4 −2 0 2 4 6

Supplemental Figure S9; Pairwise incidence of postoperative atrial fibrillation

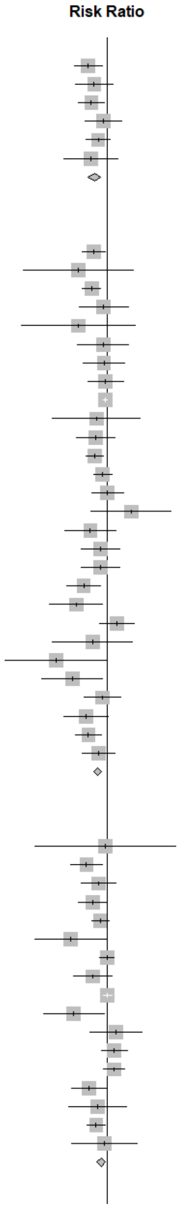

Study	logRR	SE(logRR)	RR	95%-CI
**Colchicine vs Control**				
Sarzaeem2014	-0.7239	0.2726	0.48	[0.28; 0.83]
Shvartz2001	-0.4910	0.3717	0.61	[0.30; 1.27]
Shvartz2022	-0.6023	0.2481	0.55	[0.34; 0.89]
Tabbalat12020	-0.1318	0.3570	0.88	[0.44; 1.76]
Tabbalat2015	-0.3417	0.2332	0.71	[0.45; 1.12]
Zarpelon2016	-0.6164	0.5315	0.54	[0.19; 1.53]
Random effects model			0.61	[0.48; 0.78]
Heterogeneity: $I^2 = 0\%$, $\tau^2 = 0$, $p = 0.79$				
**Corticosteroids vs Control**				
Abbaszadeh2012	-0.5108	0.2333	0.60	[0.38; 0.95]
Abd El-Hakeem2003	-1.0986	1.0646	0.33	[0.04; 2.69]
Al-Shawabkeh2017	-0.5909	0.1772	0.55	[0.39; 0.78]
Amr2009	-0.1335	0.4773	0.88	[0.34; 2.23]
Bingol2005	-1.0986	1.1106	0.33	[0.04; 2.94]
Celik2004	-0.1542	0.4928	0.86	[0.33; 2.25]
Chaney1998	-0.1178	0.4116	0.89	[0.40; 1.99]
Chaney2001	-0.0572	0.3463	0.94	[0.48; 1.86]
Dieleman2012	-0.0614	0.0416	0.94	[0.87; 1.02]
Enc2006	-0.4055	0.8563	0.67	[0.12; 3.57]
Gomez Polo2017	-0.4418	0.3796	0.64	[0.31; 1.35]
Halonen2007	-0.4686	0.1686	0.63	[0.45; 0.87]
Halvorsen2003	-0.1613	0.1808	0.85	[0.60; 1.21]
Kilger2011	0.0220	0.3106	1.02	[0.56; 1.88]
Lomivorotov2012	0.9163	0.7804	2.50	[0.54; 11.54]
Mardani2013	-0.6376	0.4976	0.53	[0.20; 1.40]
Mirhosseini2010	-0.2624	0.3789	0.77	[0.37; 1.62]
Mirhosseini2011	-0.2624	0.3789	0.77	[0.37; 1.62]
Prasongsukarn2005	-0.8938	0.3317	0.41	[0.21; 0.78]
Rubens2005	-1.1787	0.5178	0.31	[0.11; 0.85]
Schurr2001	0.3677	0.3381	1.44	[0.74; 2.80]
Sobieski2008	-0.5500	0.7787	0.58	[0.13; 2.65]
Suezawa2013	-1.9459	1.0048	0.14	[0.02; 1.02]
vukovic2010	-1.3344	0.5950	0.26	[0.08; 0.85]
Wang2016	-0.1671	0.3575	0.85	[0.42; 1.71]
Weis2009	-0.8044	0.4342	0.45	[0.19; 1.05]
Yared12000	-0.7133	0.2609	0.49	[0.29; 0.82]
Yared2007	-0.3257	0.3254	0.72	[0.38; 1.37]
Random effects model			0.70	[0.60; 0.82]
Heterogeneity: $I^2 = 44\%$, $\tau^2 = 0.0518$, $p < 0.01$				
**Fish oil vs Control**				
Berger2013	-0.0645	1.3678	0.94	[0.06; 13.68]
Calo2005	-0.7859	0.3088	0.46	[0.25; 0.83]
Castillo2010	-0.3102	0.3391	0.73	[0.38; 1.43]
Farahani2017	-0.5490	0.2887	0.58	[0.33; 1.02]
Farquharson2011	-0.2666	0.1686	0.77	[0.55; 1.07]
Feguri2019	-1.3863	0.6944	0.25	[0.06; 0.97]
Heidarsdottir2010	0.0018	0.1419	1.00	[0.76; 1.32]
Heidt2009	-0.5500	0.3722	0.58	[0.28; 1.20]
Joss2017	0.0162	0.0872	1.02	[0.86; 1.21]
Kolesnikov2015	-1.2740	0.5958	0.28	[0.09; 0.90]
Lomivorotov2014	0.3365	0.5133	1.40	[0.51; 3.83]
Saravanan12016	0.2803	0.2586	1.32	[0.80; 2.20]
Saravanan2010	0.2568	0.2027	1.29	[0.87; 1.92]
Sorice2011	-0.6905	0.3356	0.50	[0.26; 0.97]
Stanger2014	-0.3542	0.5604	0.70	[0.23; 2.10]
Wilbring2014	-0.4162	0.1826	0.66	[0.46; 0.94]
Yamamoto2014	-0.1054	0.6325	0.90	[0.26; 3.11]
Random effects model			0.79	[0.66; 0.95]
Heterogeneity: $I^2 = 50\%$, $\tau^2 = 0.0564$, $p = 0.01$				

**Fish oil + vitamin C vs Control**				
Stanger2014	0.3167	0.4480	1.37	[0.57; 3.30]
**Fish oil + vitamin C + vitamin E vs Control**				
Rodrigo2013	-1.1927	0.3340	0.30	[0.16; 0.58]
**NAC vs Control**				
El-Hamamsy2007	-0.5390	0.6422	0.58	[0.17; 2.05]
ERDIL2016	1.2040	0.7707	3.33	[0.74; 15.10]
Eren2003	0.6931	1.1402	2.00	[0.21; 18.69]
Kazemi2013	-0.3054	0.3277	0.74	[0.39; 1.40]
Kim2011	-0.6931	0.5401	0.50	[0.17; 1.44]
Orhan2006	-1.0986	1.5736	0.33	[0.02; 7.28]
Ozaydin2008	-1.4037	0.6180	0.25	[0.07; 0.82]
soleimani2018	-1.2212	0.5464	0.29	[0.10; 0.86]
Wijeysundera2007	-0.1598	0.1199	0.85	[0.67; 1.08]
**Random effects model**			0.67	[0.45; 1.01]
Heterogeneity: $I^2 = 37\%$, $\tau^2 = 0.1148$, $p = 0.12$				
**NSAIDs vs Control**				
Cheruku2004	-1.0696	0.4811	0.34	[0.13; 0.88]
Erkut2019	-1.0858	0.2170	0.34	[0.22; 0.52]
Horbach2011	-0.7304	0.4745	0.48	[0.19; 1.22]
**Random effects model**			0.36	[0.25; 0.51]
Heterogeneity: $I^2 = 0\%$, $\tau^2 = 0$, $p = 0.79$				
**Statin vs Control**				
abdallah2019	-0.8801	0.4153	0.41	[0.18; 0.94]
aydin2015	-0.9555	0.4585	0.38	[0.16; 0.94]
baran2011	-1.9459	1.0374	0.14	[0.02; 1.09]
Carrascal2016	-0.2560	0.3511	0.77	[0.39; 1.54]
chello2006	-0.9163	0.7746	0.40	[0.09; 1.83]
Dehghani2015	-0.7732	0.4179	0.46	[0.20; 1.05]
Ji2009	-0.8615	0.3390	0.42	[0.22; 0.82]
mannacio2008	-0.6650	0.2532	0.51	[0.31; 0.84]
Mansour2016	-0.5754	0.3060	0.56	[0.31; 1.02]
Patti2006	-0.4900	0.1626	0.61	[0.45; 0.84]
Rubanenko2015	-0.8907	0.3376	0.41	[0.21; 0.80]
Sun2011	-0.8073	0.3445	0.45	[0.23; 0.88]
vukovic2010	-1.0467	0.5203	0.35	[0.13; 0.97]
zheng2016	0.0321	0.0890	1.03	[0.87; 1.23]
**Random effects model**			0.53	[0.40; 0.69]
Heterogeneity: $I^2 = 65\%$, $\tau^2 = 0.1361$, $p < 0.01$				
**statin + Vitamin C vs Control**				
Samadikhan2014	-0.9163	0.4472	0.40	[0.17; 0.96]
**vitamin C vs Control**				
Dehghani2014	-1.3863	0.5220	0.25	[0.09; 0.70]
Eslami2007	-1.8718	0.7328	0.15	[0.04; 0.65]
Mirmohammadsadeghi2018	-0.0382	0.3923	0.96	[0.45; 2.08]
moludi2016	-0.4371	0.1443	0.65	[0.49; 0.86]
Papoulidis2010	-0.3137	0.1484	0.73	[0.55; 0.98]
Sadeghpour2014	-0.4575	0.1435	0.63	[0.48; 0.84]
Sarzaeem12014	-0.8210	0.3277	0.44	[0.23; 0.84]
Stanger2014	-0.1310	0.5138	0.88	[0.32; 2.40]
Wang12020	0.1144	0.3777	1.12	[0.53; 2.35]
**Random effects model**			0.64	[0.52; 0.80]
Heterogeneity: $I^2 = 37\%$, $\tau^2 = 0.0351$, $p = 0.12$				
**Vitamin D vs Control**				
Talasaz2021	-0.7452	0.3718	0.47	[0.23; 0.98]
**Corticosteroids vs Statin**				
vukovic2010	-0.2877	0.7172	0.75	[0.18; 3.06]
**Fish oil vs Fish oil + vitamin C**				
Stanger2014	-0.6708	0.5305	0.51	[0.18; 1.45]
**Fish oil vs vitamin C**				
Stanger2014	-0.2231	0.5871	0.80	[0.25; 2.53]
**Fish oil + vitamin C vs vitamin C**				
Stanger2014	0.4477	0.4810	1.56	[0.61; 4.02]

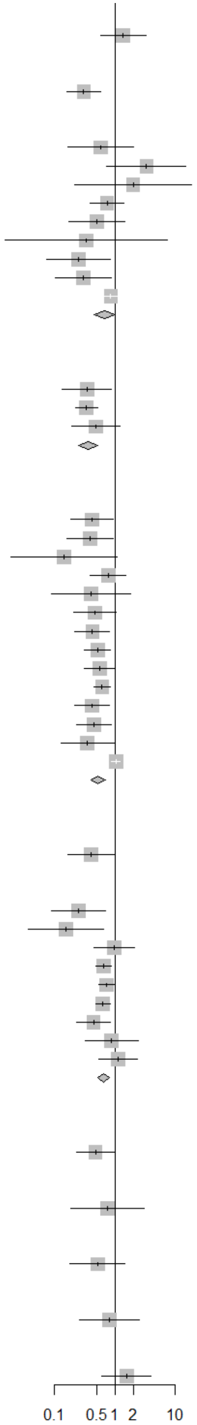

Supplemental Figure S10; Pairwise incidence of postoperative all-cause mortality

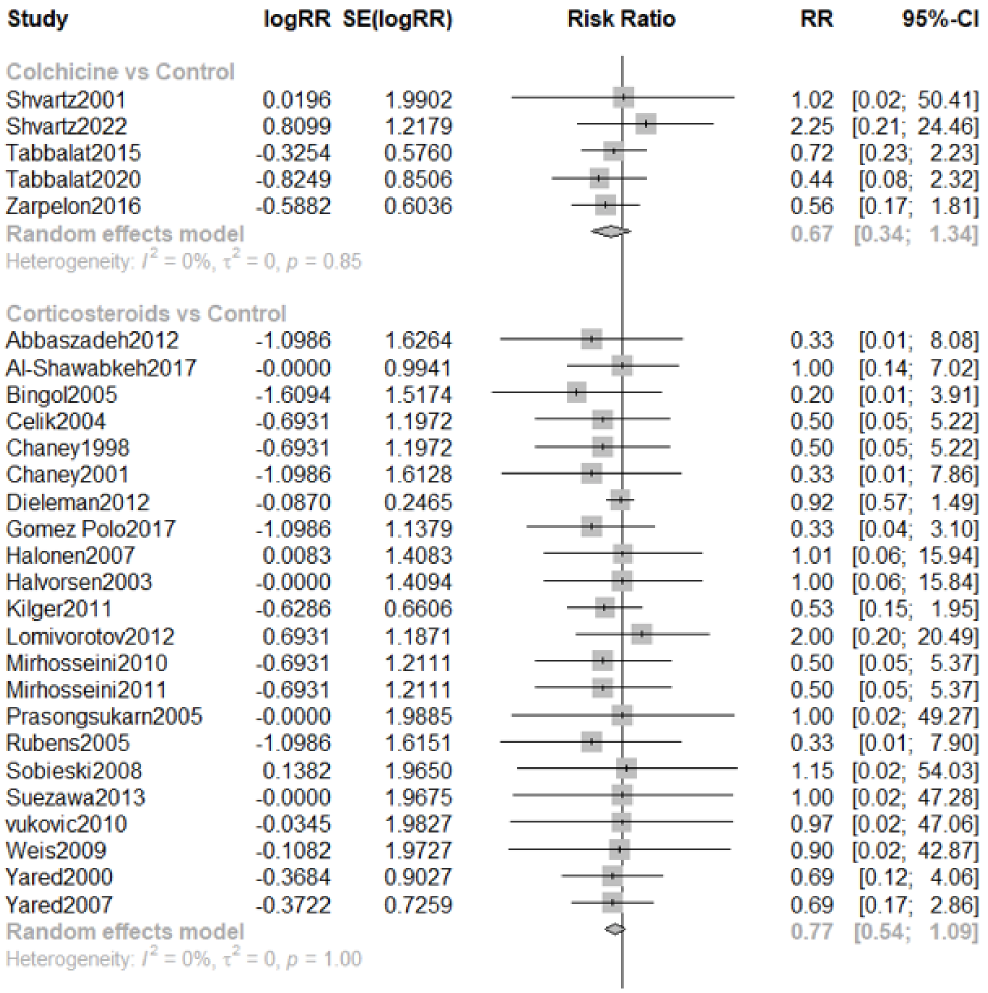

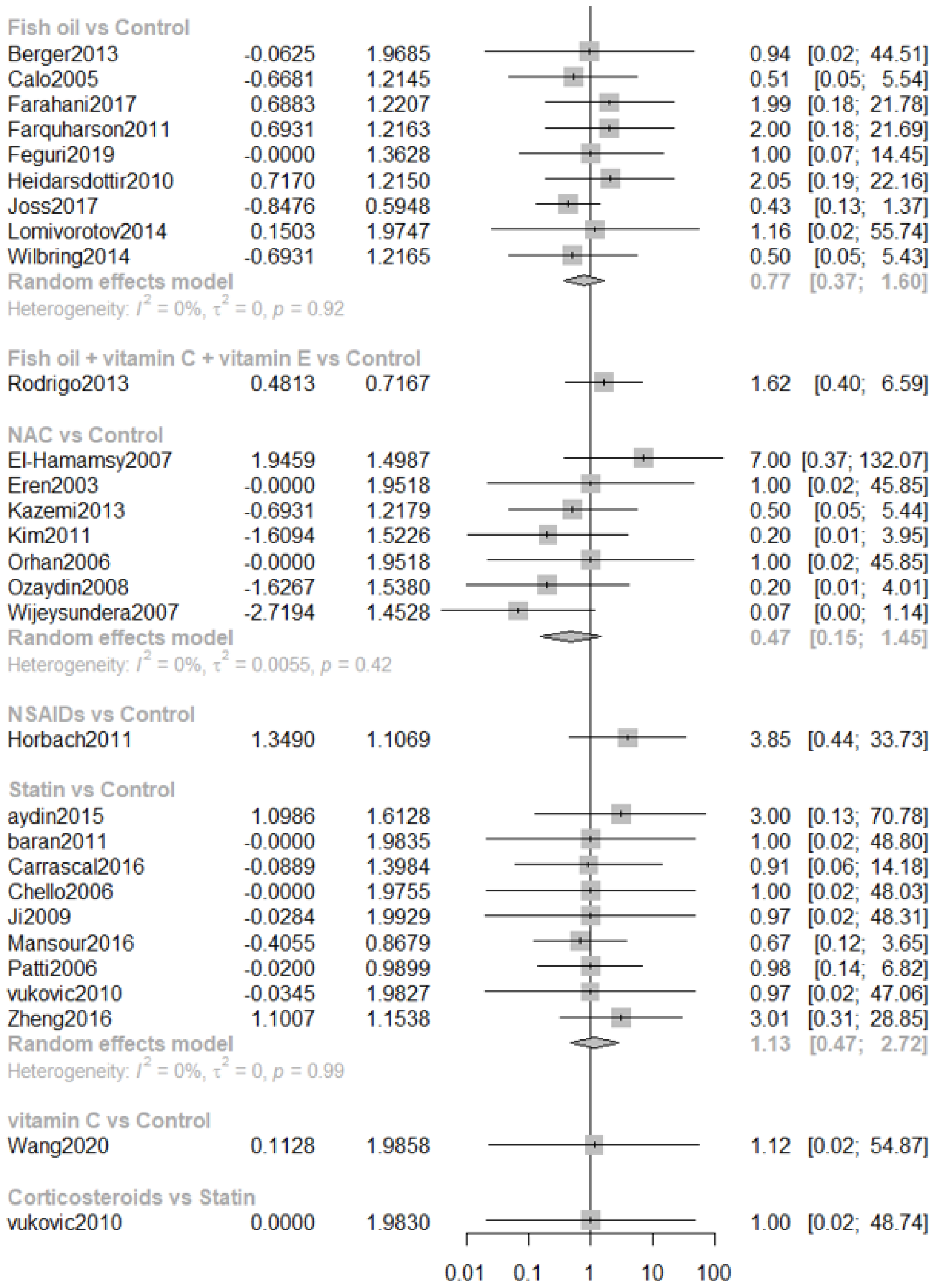
Fish oil vs Control
Berger2013 -0.0625 1.9685 0.94 [0.02; 44.51]
Calo2005 -0.6681 1.2145 0.51 [0.05; 5.54]
Farahani2017 0.6883 1.2207 1.99 [0.18; 21.78]
Farquharson2011 0.6931 1.2163 2.00 [0.18; 21.69]
Feguri2019 -0.0000 1.3628 1.00 [0.07; 14.45]
Heidarsdottir2010 0.7170 1.2150 2.05 [0.19; 22.16]
Joss2017 -0.8476 0.5948 0.43 [0.13; 1.37]
Lomivorotov2014 0.1503 1.9747 1.16 [0.02; 55.74]
Wilbring2014 -0.6931 1.2165 0.50 [0.05; 5.43]
Random effects model 0.77 [0.37; 1.60]
Heterogeneity: I^2 = 0%, τ^2 = 0, p = 0.92
Fish oil + vitamin C + vitamin E vs Control
Rodrigo2013 0.4813 0.7167 1.62 [0.40; 6.59]
NAC vs Control
El-Hamamsy2007 1.9459 1.4987 7.00 [0.37; 132.07]
Eren2003 -0.0000 1.9518 1.00 [0.02; 45.85]
Kazemi2013 -0.6931 1.2179 0.50 [0.05; 5.44]
Kim2011 -1.6094 1.5226 0.20 [0.01; 3.95]
Orhan2006 -0.0000 1.9518 1.00 [0.02; 45.85]
Ozaydin2008 -1.6267 1.5380 0.20 [0.01; 4.01]
Wijeysundera2007 -2.7194 1.4528 0.07 [0.00; 1.14]
Random effects model 0.47 [0.15; 1.45]
Heterogeneity: I^2 = 0%, τ^2 = 0.0055, p = 0.42
NSAIDs vs Control
Horbach2011 1.3490 1.1069 3.85 [0.44; 33.73]
Statin vs Control
aydin2015 1.0986 1.6128 3.00 [0.13; 70.78]
baran2011 -0.0000 1.9835 1.00 [0.02; 48.80]
Carrascal2016 -0.0889 1.3984 0.91 [0.06; 14.18]
Chello2006 -0.0000 1.9755 1.00 [0.02; 48.03]
Ji2009 -0.0284 1.9929 0.97 [0.02; 48.31]
Mansour2016 -0.4055 0.8679 0.67 [0.12; 3.65]
Patti2006 -0.0200 0.9899 0.98 [0.14; 6.82]
vukovic2010 -0.0345 1.9827 0.97 [0.02; 47.06]
Zheng2016 1.1007 1.1538 3.01 [0.31; 28.85]
Random effects model 1.13 [0.47; 2.72]
Heterogeneity: I^2 = 0%, τ^2 = 0, p = 0.99
vitamin C vs Control
Wang2020 0.1128 1.9858 1.12 [0.02; 54.87]
Corticosteroids vs Statin
vukovic2010 0.0000 1.9830 1.00 [0.02; 48.74]
0.01 0.1 1 10 100

Supplemental Figure S11; Pairwise incidence of postoperative serious adverse events

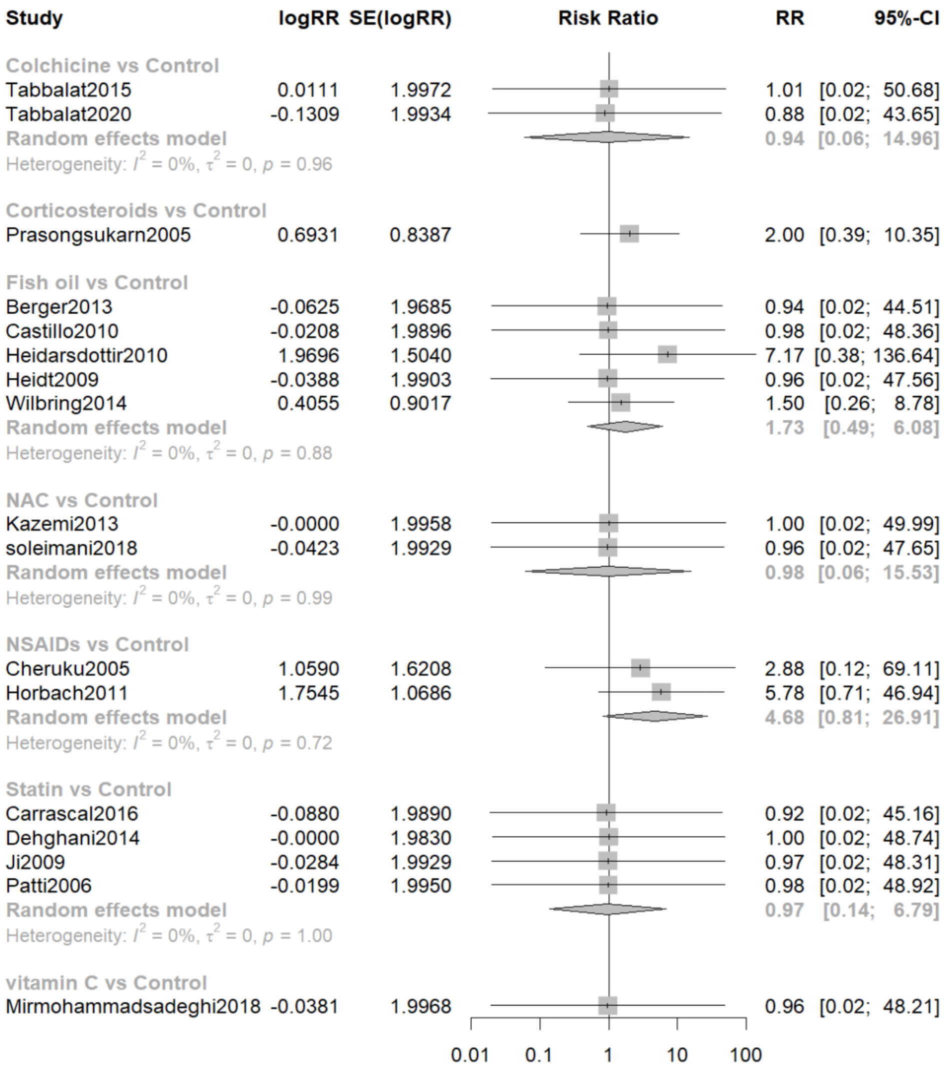

Supplemental Figure S12; Pairwise estimates for duration of hospitalization

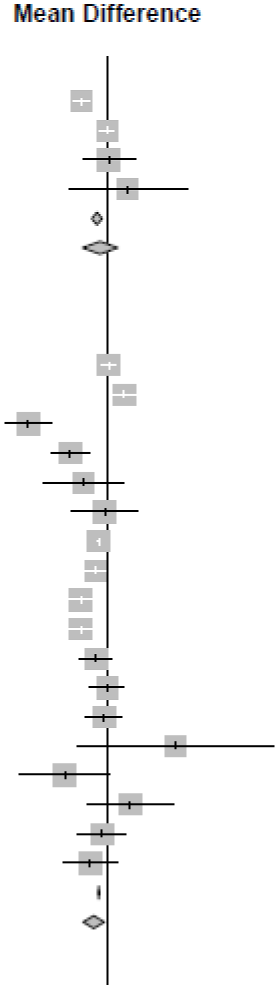

Study	MD	SE(MD)	MD	95%-CI
**Colchicine:Control**				
sarzaeem 2014	−1.5000	0.2406	−1.50	[−1.97; −1.03]
shvartz−le 2022	0.0000	0.2118	0.00	[−0.42; 0.42]
Tabbalat 2015	0.1000	0.7837	0.10	[−1.44; 1.64]
Zarpelon 2016	1.2000	1.7729	1.20	[−2.27; 4.67]
Common effect model			**−0.61**	**[−0.92; −0.31]**
Random effects model			**−0.43**	**[−1.47; 0.62]**
Heterogeneity: $I^2 = 87\%$, $\tau^2 = 0.7369$, $p < 0.01$				
**Corticosteroids:Control**				
Al−Shawabkeh 2017	0.0700	0.2284	0.07	[−0.38; 0.52]
Amr 2009	1.0000	0.3162	1.00	[0.38; 1.62]
Bingol 2005	−4.6500	0.7096	−4.65	[−6.04; −3.26]
Celik 2004	−2.2000	0.5811	−2.20	[−3.34; −1.06]
Chaney 1998	−1.4000	1.1947	−1.40	[−3.74; 0.94]
Chaney 2001	−0.1500	0.9997	−0.15	[−2.11; 1.81]
Dieleman 2012	−0.5000	0.0260	−0.50	[−0.55; −0.45]
Mardani 2013	−0.7100	0.2931	−0.71	[−1.28; −0.14]
Mirhosseini 2010	−1.5800	0.3100	−1.58	[−2.19; −0.97]
Mirhosseini 2011	−1.5800	0.3100	−1.58	[−2.19; −0.97]
Rubens 2005	−0.6500	0.4761	−0.65	[−1.58; 0.28]
Schurr 2001	0.0000	0.5149	0.00	[−1.01; 1.01]
Sobieski 2008	−0.2000	0.5345	−0.20	[−1.25; 0.85]
Suezawa 2013	4.0000	2.9439	4.00	[−1.77; 9.77]
vukovic 2010	−2.5000	1.3447	−2.50	[−5.14; 0.14]
Weis 2009	1.3333	1.2991	1.33	[−1.21; 3.88]
Yared 2000	−0.3000	0.7145	−0.30	[−1.70; 1.10]
Yared 2007	−1.0000	0.8269	−1.00	[−2.62; 0.62]
Common effect model			**−0.51**	**[−0.56; −0.46]**
Random effects model			**−0.80**	**[−1.43; −0.17]**
Heterogeneity: $I^2 = 84\%$, $\tau^2 = 1.3473$, $p < 0.01$				

Supplemental Figure S13; Pairwise estimates for duration of hospitalization

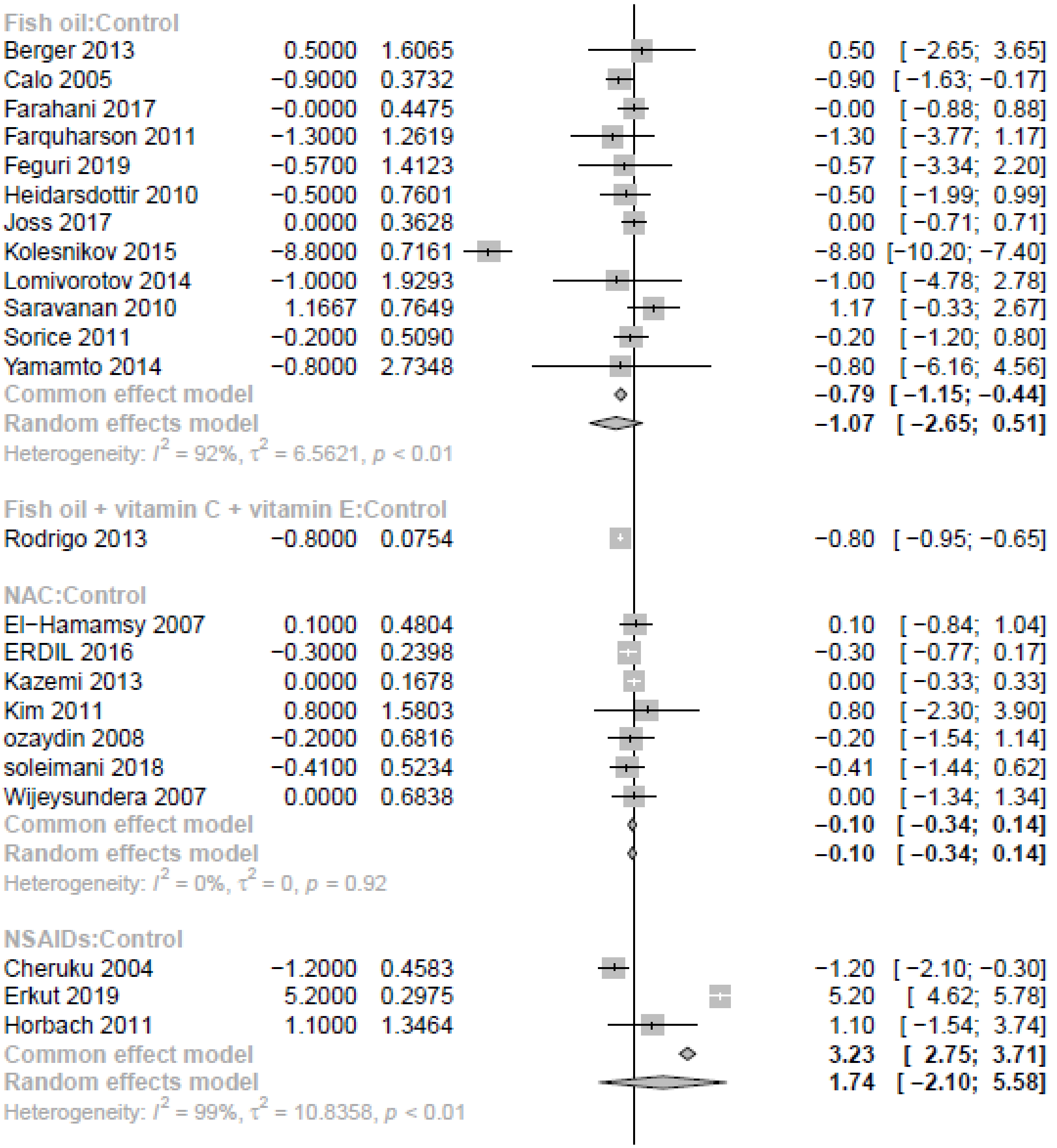

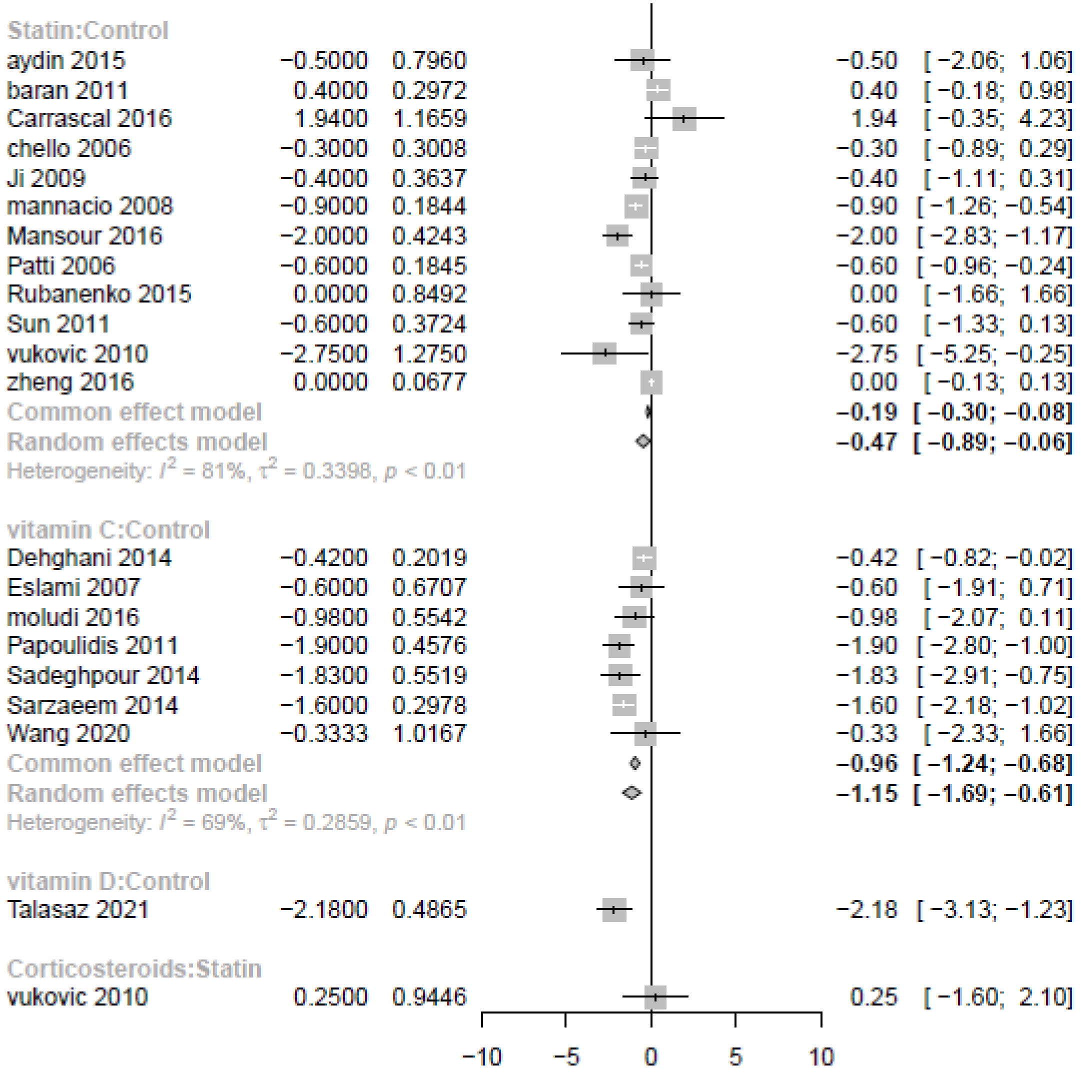
Statin:Control
aydin 2015 −0.5000 0.7960 −0.50 [−2.06; 1.06]
baran 2011 0.4000 0.2972 0.40 [−0.18; 0.98]
Carrascal 2016 1.9400 1.1659 1.94 [−0.35; 4.23]
chello 2006 −0.3000 0.3008 −0.30 [−0.89; 0.29]
Ji 2009 −0.4000 0.3637 −0.40 [−1.11; 0.31]
mannacio 2008 −0.9000 0.1844 −0.90 [−1.26; −0.54]
Mansour 2016 −2.0000 0.4243 −2.00 [−2.83; −1.17]
Patti 2006 −0.6000 0.1845 −0.60 [−0.96; −0.24]
Rubanenko 2015 0.0000 0.8492 0.00 [−1.66; 1.66]
Sun 2011 −0.6000 0.3724 −0.60 [−1.33; 0.13]
vukovic 2010 −2.7500 1.2750 −2.75 [−5.25; −0.25]
zheng 2016 0.0000 0.0677 0.00 [−0.13; 0.13]
Common effect model −0.19 [−0.30; −0.08]
Random effects model −0.47 [−0.89; −0.06]
Heterogeneity: I^2 = 81%, τ^2 = 0.3398, p < 0.01
vitamin C:Control
Dehghani 2014 −0.4200 0.2019 −0.42 [−0.82; −0.02]
Eslami 2007 −0.6000 0.6707 −0.60 [−1.91; 0.71]
moludi 2016 −0.9800 0.5542 −0.98 [−2.07; 0.11]
Papoulidis 2011 −1.9000 0.4576 −1.90 [−2.80; −1.00]
Sadeghpour 2014 −1.8300 0.5519 −1.83 [−2.91; −0.75]
Sarzaeem 2014 −1.6000 0.2978 −1.60 [−2.18; −1.02]
Wang 2020 −0.3333 1.0167 −0.33 [−2.33; 1.66]
Common effect model −0.96 [−1.24; −0.68]
Random effects model −1.15 [−1.69; −0.61]
Heterogeneity: I^2 = 69%, τ^2 = 0.2859, p < 0.01
vitamin D:Control
Talasaz 2021 −2.1800 0.4865 −2.18 [−3.13; −1.23]
Corticosteroids:Statin
vukovic 2010 0.2500 0.9446 0.25 [−1.60; 2.10]
−10
−5
0
5
10

Supplemental Table S5; League table: Network meta-analysis results for incidence of postoperative atrial fibrillation

**Colchicine**										
0.91 (0.61 ,1.35)	**Corticosteroids**									
0.81 (0.54 ,1.21)	0.89 (0.69 ,1.14)	**Fish oil**								
0.5 (0.2 ,1.23)	0.55 (0.24 ,1.27)	0.62 (0.27 ,1.42)	**Fish oil + vitamin C**							
2.1 (0.86 ,5.1)	**2.3 (1 ,5.28)**	**2.59 (1.13 ,5.98)**	**4.2 (1.32 ,13.35)**	**Fish oil + vitamin C + vitamin E**						
0.92 (0.56 ,1.51)	1.01 (0.69 ,1.48)	1.14 (0.77 ,1.68)	1.84 (0.75 ,4.49)	0.44 (0.18 ,1.06)	**NAC**					
1.74 (0.95 ,3.19)	**1.91 (1.14 ,3.19)**	**2.15 (1.28 ,3.63)**	**3.48 (1.34 ,9.06)**	0.83 (0.32 ,2.14)	**1.89 (1.04 ,3.44)**	**NSAIDs**				
1.13 (0.74 ,1.72)	1.24 (0.95 ,1.63)	**1.4 (1.05 ,1.87)**	2.26 (0.97 ,5.31)	0.54 (0.23 ,1.25)	1.23 (0.82 ,1.86)	0.65 (0.38 ,1.11)	**Statin**			
1.59 (0.55 ,4.61)	1.75 (0.63 ,4.81)	1.97 (0.71 ,5.45)	3.18 (0.87 ,11.63)	0.76 (0.21 ,2.75)	1.73 (0.6 ,5)	0.91 (0.3 ,2.78)	1.41 (0.5 ,3.92)	**Statin + vitamin C**		
0.99 (0.64 ,1.54)	1.09 (0.81 ,1.47)	1.23 (0.9 ,1.67)	1.99 (0.86 ,4.58)	0.47 (0.2 ,1.11)	1.08 (0.7 ,1.66)	**0.57 (0.33 ,0.99)**	0.88 (0.63 ,1.22)	0.62 (0.22 ,1.75)	**Vitamin C**	
**0.62 (0.45 ,0.85)**	**0.7 (0.59 ,0.82)**	**0.79 (0.65 ,0.95)**	1.27 (0.56 ,2.9)	**0.3 (0.13 ,0.68)**	**0.69 (0.49 ,0.98)**	**0.37 (0.23 ,0.59)**	**0.56 (0.45 ,0.7)**	0.4 (0.15 ,1.08)	**0.64 (0.5 ,0.82)**	**Control**

Footnote: Results are Risk Ratio (95% CIs) from the network meta-analysis. For each comparison (column vs row) an RR < 1 indicates the intervention in the column provides better prophylaxis than the comparator in the column. Numbers in bold represent statistically significant results.

Supplemental Table S6*;* League table: Network meta-analysis results for postoperative all-cause mortality

**Colchicine**								
0.78 (0.28 ,2.18)	**Fish oil**							
0.79 (0.35 ,1.75)	1.01 (0.45, 2.26)	**Corticosteroid s**						
0.54 (0.17 ,1.66)	0.68 (0.22 ,2.13)	0.68 (0.27 ,1.74)	**Statin**					
1.3 (0.34 ,4.98)	1.66 (0.43 ,6.40)	1.65 (0.50 ,5.41)	2.44 (0.58, 10)	**NAC**				
0.54 (0.01 ,28.25)	0.69 (0.01 ,36.16)	0.69 (0.01 ,34.13)	1 (0.02 ,54.07)	0.42 (0.007 ,23.99)	**Vitamin C**			
0.37 (0.08 ,1.81)	0.48 (0.10 ,2.32)	0.47 (0.11 ,2.02)	0.7 (0.13, 3.7)	0.28 (0.05, 1.75)	0.69 (0.01, 50)	**Fish oil + vitamin C + vitamin E**		
0.35 (0.05 ,2.38)	0.44 (0.06,3.05)	0.44 (0.07 ,2.72)	0.64 (0.08, 4.76)	0.27 (0.032 ,2.22)	0.64 (0.009, 50)	0.93 (0.09 ,9.02)	**NSAIDs**	
0.67 (0.34 ,1.34)	0.77 (0.37 ,1.6)	0.77 (0.54 ,1.09)	1.12 (0.47 ,2.67)	0.47 (0.15 ,1.45)	1.12 (0.02 ,54.87)	1.62 (0.4 ,6.59)	3.85 (0.44 ,33.73)	**Control**

Footnote: Results are Risk Ratio (95% CIs) from the network meta-analysis. For each comparison (column vs row) an RR < 1 indicates the intervention in the column provides better prophylaxis than the comparator in the column. Numbers in bold represent statistically significant results.

Supplemental Table S7*;* League table: Network meta-analysis results for postoperative serious adverse events

**Colchicine**							
0.47 (0.02 ,11.75)	**Corticosteroids**						
0.54 (0.03 ,11.33)	1.15 (0.15 ,9.14)	**Fish oil**					
0.96 (0.02 ,47.99)	2.04 (0.08 ,50.92)	1.77 (0.09 ,36.85)	**NAC**				
0.2 (0.01 ,5.3)	0.43 (0.04 ,4.71)	0.37 (0.04 ,3.19)	0.21 (0.01 ,5.51)	**NSAIDs**			
0.97 (0.03 ,28.73)	2.07 (0.16 ,26.52)	1.79 (0.18 ,18.23)	1.01 (0.03 ,29.83)	4.84 (0.35 ,66.49)	**Statin**		
0.98 (0.01 ,117.94)	2.08 (0.03 ,144.92)	1.8 (0.03 ,109.72)	1.02 (0.01 ,122.52)	4.86 (0.07 ,353.69)	1 (0.01 ,79.58)	**Vitamin C**	
0.94 (0.06 ,14.96)	2 (0.39 ,10)	1.72 (0.49 ,6.25)	0.98 (0.06 ,16.67)	4.76 (0.81 ,25)	0.97 (0.14 ,6.67)	0.96 (0.02 ,50)	**Control**

Footnote: Results are Risk Ratio (95% CIs) from the network meta-analysis. For each comparison (column vs row) an RR < 1 indicates the intervention in the column provides better prophylaxis than the comparator in the column. Numbers in bold represent statistically significant results.

Supplemental Table S8; League table: Network meta-analysis results for duration of hospitalization

**Colchicine**										
0.46 (-1.33 ,2.25)	**Corticosteroid s**									
0.81 (-1.1 ,2.72)	0.34 (-0.91 ,1.6)	**Fish oil**								
0.51 (-2.79 ,3.82)	0.05 (-2.92 ,3.03)	-0.29 (-3.34 ,2.76)	**Fish oil + vitamin C + vitamin E**							
-0.22 (-2.24 ,1.79)	-0.69 (-2.09 ,0.72)	-1.03 (-2.59 ,0.52)	-0.74 (-3.85 ,2.38)	**NAC**						
-2.15 (-4.61 ,0.31)	**-2.61 (-4.61, -0.61)**	**-2.96 (-5.06, -0.85)**	-2.66 (-6.09 ,0.76)	-1.92 (-4.12 ,0.27)	**NSAID**					
0.17 (-1.67 ,2.02)	-0.29 (-1.42 ,0.84)	-0.64 (-1.97 ,0.7)	-0.34 (-3.35 ,2.67)	0.4 (-1.08 ,1.87)	**2.32 (0.27 ,4.37)**	**Statin**				
0.84 (-1.16 ,2.84)	0.38 (-1.01 ,1.76)	0.03 (-1.51 ,1.57)	0.32 (-2.78 ,3.43)	1.06 (-0.6 ,2.73)	**2.99 (0.8 ,5.17)**	0.67 (-0.79 ,2.12)	**Vitamin C**			
1.89 (-1.54 ,5.33)	1.43 (-1.69 ,4.55)	1.09 (-2.1 ,4.28)	1.38 (-2.8 ,5.56)	2.12 (-1.14 ,5.37)	**4.04 (0.49 ,7.59)**	1.72 (-1.43 ,4.88)	1.06 (-2.19 ,4.3)	**Vitamin D**		
-0.29 (-1.91 ,1.34)	-0.75 (-1.5 ,0)	**-1.09 (-2.1, -0.09)**	-0.8 (-3.68 ,2.08)	-0.06 (-1.25 ,1.13)	**1.86 (0.01 ,3.71)**	-0.46 (-1.34 ,0.42)	-1.12 (-2.29 ,0.04)	-2.18 (-5.21 ,0.85)	**Control**	

Footnote: Results are mean difference (95% CIs) from the network meta-analysis. For each comparison (column vs row) an MD < 0 indicates the intervention in the column provides better prophylaxis than the comparator in the column. Numbers in bold represent statistically significant results.

Supplemental Table S9; Adverse events due to interventions

Study	Intervention	Adverse event 1	rate (%)	Adverse event 2	rate (%)	Adverse event 3	rate (%)	Adverse event 4	rate (%)	Adverse event 5	rate (%)	Adverse event 6	rate (%)
**8-Feguri 2017**	Fish oil	Postoperative nausea and vomiting	35.71										
	Control	Postoperative nausea and vomiting	28.57										
**41-Tabbalat 2020**	Colchicine	Diarrhea	1.23	Diaphoresis	1.23								
	Control	Diarrhea	1.41	Diaphoresis	1.41								
**42-Tabbalat 2015**	Colchicine	Diarrhea	24.58	Anorexia	6.15								
	Control	Diarrhea	5.52	Anorexia	2.21								
**60-Yared 2007**	Corticosteroids	Hyperglycemia	64.86										
	Control	Hyperglycemia	0										
**64-Weis 2009**	Corticosteroids	Postoperative sepsis	15.79	Pneumonia	10.53	Sternal wound infection	0	Catheter-related infection	5.26				
	Control	Postoperative sepsis	17.65	Pneumonia	11.76	Sternal wound infection	5.88	Catheter-related infection	0				
**102-Rodrigo 2013**	Fish oil + vitamin C + vitamin E	Dyspepsia	3.88	Diarrhea	2.91								
	Control	Dyspepsia	3	Diarrhea	2.91								
**151-Joss 2017**	Fish oil	Surgical bleeding	10										
	Control	Surgical bleeding	4.91										
**152-Ji 2009**	Statin	GI bleeding	0										
	Control	GI bleeding	0										
**156-Horbach 2011**	NSAIDs	Renal failure	7.32										
	Control	Renal failure	1.27										

**163-Halonen 2007**	Corticosteroids	Superficial wound infections	14.05										
	Control	Superficial wound infections	14.05										
**173-Feguri 2019**	Fish oil	Pneumonia	14.29										
	Control	Pneumonia	7.14										
**198-Calo 2005**	Fish oil	Skin allergic reaction	1.27										
	Control	Skin allergic reaction	0										
**219-Abbaszadeh 2012**	Corticosteroids	Urinary tract infection	0	Pulmonary infection	2.17	Wound infection	3.26						
	Control	Urinary tract infection	1.09	Pulmonary infection	1.09	Wound infection	2.17						
**227-Dieleman 2012**	Corticosteroids	Wound infection	1.52	GI bleeding	0.58	Pneumonia	5.95	Urinary tract infection	2.24	Catheter related infection	0.27	Sepsis	0.81
	Control	Wound infection	1.42	GI bleeding	0.49	Pneumonia	10.59	Urinary tract infection	2.67	Catheter related infection	0.93	Sepsis	1.16
**251-Amr 2009**	Corticosteroids	Surgical bleeding	8	Pulmonary infection	8	Wound infection	0						
	Control	Surgical bleeding	6	Pulmonary infection	6	Wound infection	0						
**252-Bingol 2005**	Corticosteroids	Pleural effusion	5	Wound infection	0	Supraventricular tachycardia	0						
	Control	Pleural effusion	20	Wound infection	10	Supraventricular tachycardia	15						
**257-Enc 2006**	Corticosteroids	Hyperglycemia	5										
	Control	Hyperglycemia	0										
	Corticosteroids	Hyperglycemia	7.69	Mediastinitis	1.92	Wound infection	1.92						

**258-Gomez Polo 2017**	Control	Hyperglycemia	5.77	Mediastinitis	0	Wound infection	1.92
**259-Kilger 2011**	Corticosteroids	Hyperglycemia	16.94	Pituitary-Adrenal Axis suppression	0		
	Control	Hyperglycemia	13.11	Pituitary-Adrenal Axis suppression	0		
**260-Lomivorotov 2012**	Corticosteroids	Infectious complications	9.09				
	Control	Infectious complications	4.55				
**261-Mardani 2013**	Corticosteroids	Infectious complications (pneumonia, sepsis, leg infection)	6.98				
	Control	Infectious complications (pneumonia, sepsis, leg infection)	4				
**262-Schurr 2001**	Corticosteroids	Pulmonary infection	4.17	Urinary tract infection	4.17		
	Control	Pulmonary infection	7.69	Urinary tract infection	0		
**265-vukovic 2010**	Statin	Infectious complications	3.45				
	Corticosteroids	Infectious complications	6.9				
	Control	Infectious complications	7.14				

**270-Castillo 2010**	Fish oil	Surgical bleeding	2.08	Congestive heart failure	2.08	Mediastinitis	0						
	Control	Surgical bleeding	0	Congestive heart failure	0	Mediastinitis	4.26						
**277-Wijeysundera 2007**	NAC	Bronchospasm	4.55	Urticaria	2.27	Nausea and/or vomiting	29.55						
	Control	Bronchospasm	2.3	Urticaria	0	Nausea and/or vomiting	19.54						
**326-Shvartz 2022**	Colchicine	Nausea	12	Vomiting	2	Anorexia	22	Abdominal pain	12	Convulsions	2	Tingling in extremities	10
	Control	Nausea	9.8	Vomiting	3.92	Anorexia	27.45	Abdominal pain	3.92	Convulsions	7.84	Tingling in extremities	13.73
**327-Shvartz 2022**	Colchicine	Anorexia	16.81	Abdominal pain	7.08	Convulsions	1.77	Tingling in hands and feet	7.96	Diarrhea	25.66	Vomiting	1.77
	Control	Anorexia	18.9	Abdominal pain	1.57	Convulsions	5.51	Tingling in hands and feet	7.87	Diarrhea	11.81	Vomiting	4.72
**39-Sorice 2011**	Fish oil	The study reported that no adverse events occurred											
	Control												
**84-soleimani 2018**	NAC	The study reported that no adverse events occurred											
	Control												
**106-Pierri 2016**	Statin	The study reported that no adverse events occurred											
	Statin												
**108-Patti 2006**	Statin	The study reported that no adverse events occurred											
	Control												
**111-Ozaydin 2008**	NAC	The study reported that no adverse events occurred											

	Control	
**128-Mirmohammadsadeghi 2018**	vitamin C	The study reported that no adverse events occurred
	Control	
**146-Kazemi 2013**	NAC	The study reported that no adverse events occurred
	Control	
**158-Heidt 2009**	Fish oil	The study reported that no adverse events occurred
	Control	
**185-Dehghani 2014**	Statin	The study reported that no adverse events occurred
	Control	
**264-Suezawa 2013**	Corticosteroids	The study reported that no adverse events occurred
	Control	
**268-Chello 2006**	Statin	The study reported that no adverse events occurred
	Control	
**269-Berger 2013**	Fish oil	The study reported that no adverse events occurred
	Control	
**271-Kolesnikov 2015**	Fish oil	The study reported that no adverse events occurred
	Control	
**275-Kim 2011**	NAC	The study reported that no adverse events occurred

Supplemental Table S10; ICU readmission

Study	Intervention groups	Number of patients	Number of ICU readmissions	Follow up time (days)
**42-Kilger 2011**	Corticosteroids	183	3	30
	Control	122	0	30
**260-Lomivorotov 2012**	Corticosteroids	22	3	47
	Control	22	0	47

References

1. White CM, Caron MF, Kalus JS, et al. Intravenous plus oral amiodarone, atrial septal pacing, or both strategies to prevent post-cardiothoracic surgery atrial fibrillation: the Atrial Fibrillation Suppression Trial II (AFIST II). *Circulation*. 2003;108:II200-6.
2. Abbaszadeh M, Khan ZH, Mehrani F, Jahanmehr H. Perioperative intravenous corticosteroids reduce incidence of atrial fibrillation following cardiac surgery: a randomized study. *REVISTA BRASILEIRA DE CIRURGIA CARDIOVASCULAR*. 2012;27(1):18-23. doi:10.5935/1678-9741.20120005
3. Abd El-Hakeem EE, Ashry MA, El-Minshawy A, Maghraby EA. Influence of dexamethasone on cytokine balance in patients undergoing valve replacement surgery. *Egypt J Anaesth*. 2003;19:205-214.
4. Abd Allah E, Kamel EZ, Osman HM, et al. Could Short-Term Perioperative High-Dose Atorvastatin Offer Antiarrhythmic and Cardio-Protective Effects in Rheumatic Valve Replacement Surgery? *JOURNAL OF CARDIOTHORACIC AND VASCULAR ANESTHESIA*. 2019;33(12):3340-3347. doi:10.1053/j.jvca.2019.05.013
5. Al-Shawabkeh Z, Al-Nawaesah K, Anzeh RA, Al-Odwan H, Al-Rawashdeh WAB, Altaani H. Use of short-term steroids in the prophylaxis of atrial fibrillation after cardiac surgery. *Journal of the Saudi Heart Association*. 2017;29(1):23-29. doi:10.1016/j.jsha.2016.03.005
6. Amr YM, Elmistekawy E, El-serogy H. Effects of Dexamethasone on Pulmonary and Renal Functions in Patients Undergoing CABG With Cardiopulmonary Bypass. *Seminars in Cardiothoracic and Vascular Anesthesia*. 2009;13(4):231-237. doi:10.1177/1089253209351598
7. Aydin U, Yilmaz M, Duzyo C, et al. Efficiency of postoperative statin treatment for preventing new-onset postoperative atrial fibrillation in patients undergoing isolated coronary artery bypass grafting: A prospective randomized study. *ANATOLIAN JOURNAL OF CARDIOLOGY*. 2015;15(6):491-495. doi:10.5152/akd.2014.5531
8. Baran C, Durdu S, Dalva K, et al. Effects of Preoperative Short Term Use of Atorvastatin on Endothelial Progenitor Cells after Coronary Surgery: A Randomized, Controlled Trial. *STEM CELL REVIEWS AND REPORTS*. 2012;8(3):963-971. doi:10.1007/s12015-011-9321-z
9. Berger MM, Delodder F, Liaudet L, et al. Three short perioperative infusions of n-3 PUFAs reduce systemic inflammation induced by cardiopulmonary bypass surgery: a randomized controlled trial. *The American journal of clinical nutrition*. 2013;97(2):246-254.
10. Bingol H, Cingoz F, Balkan A, et al. The effect of oral prednisolone with chronic obstructive pulmonary disease undergoing coronary artery bypass surgery. *Journal of Cardiac Surgery*. 2005;20(3):252-256.
11. Calo L, Bianconi L, Colivicchi F, et al. N-3 fatty acids for the prevention of atrial fibrillation after coronary artery bypass surgery - A randomized, controlled trial. *JOURNAL OF THE AMERICAN COLLEGE OF CARDIOLOGY*. 2005;45(10):1723-1728. doi:10.1016/j.jacc.2005.02.079
12. Carrascal Y, Arnold RJ, De la Fuente L, et al. Efficacy of atorvastatin in prevention of atrial fibrillation after heart valve surgery in the PROFACE trial (PROphylaxis of postoperative atrial Fibrillation After Cardiac surgEry). *Journal of arrhythmia*. 2016;32(3):191-7. doi:https://dx.doi.org/10.1016/j.joa.2016.01.010

13. Castillo R, Rodrigo R, Perez F, et al. Antioxidant therapy reduces oxidative and inflammatory tissue damage in patients subjected to cardiac surgery with extracorporeal circulation. *Basic & clinical pharmacology & toxicology*. 2011;108(4):256-262.
14. Celik JB, Gormus N, Okesli S, Gormus ZI, Solak H. Methylprednisolone prevents inflammatory reaction occurring during cardiopulmonary bypass: effects on TNF-α, IL-6, IL-8, IL-10. *Perfusion*. 2004;19(3):185-191.
15. Chaney MA, Nikolov MP, Blakeman B, Bakhos M, Slogoff S. Pulmonary effects of methylprednisolone in patients undergoing coronary artery bypass grafting and early tracheal extubation. *Anesthesia & Analgesia*. 1998;87(1):27-33.
16. Chaney MA, Durazo-Arvizu RA, Nikolov MP, Blakeman BP, Bakhos M. Methylprednisolone does not benefit patients undergoing coronary artery bypass grafting and early tracheal extubation. *The Journal of thoracic and cardiovascular surgery*. 2001;121(3):561-569.
17. Chello M, Patti G, Candura D, et al. Effects of atorvastatin on systemic inflammatory response after coronary bypass surgery. *Critical care medicine*. 2006;34(3):660-667.
18. Cheruku KK, Ghani A, Ahmad F, et al. Efficacy of nonsteroidal anti-inflammatory medications for prevention of atrial fibrillation following coronary artery bypass graft surgery. *Preventive cardiology*. 2004;7(1):13-18. doi:10.1111/j.1520-037x.2004.3117.x
19. Dehghani MR, Majidi N, Rahmani A, Asgari B, Rezaei Y. Effect of oral vitamin C on atrial fibrillation development after isolated coronary artery bypass grafting surgery: A prospective randomized clinical trial. *CARDIOLOGY JOURNAL*. 2014;21(5):492-499. doi:10.5603/CJ.a2013.0154
20. Dehghani MR, Kasianzadeh M, Rezaei Y, Sepehrvand N. Atorvastatin Reduces the Incidence of Postoperative Atrial Fibrillation in Statin-Naive Patients Undergoing Isolated Heart Valve Surgery: A Double-Blind, Placebo-Controlled Randomized Trial. *JOURNAL OF CARDIOVASCULAR PHARMACOLOGY AND THERAPEUTICS*. 2015;20(5):465-472. doi:10.1177/1074248414564869
21. Dieleman JM, Nierich AP, Rosseel PM, et al. Intraoperative high-dose dexamethasone for cardiac surgery: a randomized controlled trial. *Jama*. 2012;308(17):1761-1767.
22. El-Hamamsy I, Stevens L-M, Carrier M, et al. Effect of intravenous N-acetylcysteine on outcomes after coronary artery bypass surgery: a randomized, double-blind, placebo-controlled clinical trial. *The Journal of thoracic and cardiovascular surgery*. 2007;133(1):7-12.
23. Elmarsafawi AG, Abbassi MM, Elkaffas S, Elsawy HM, Sabry NA. Efficacy of Different Perioperative Statin Regimens on Protection Against Post-Coronary Artery Bypass Grafting Major Adverse Cardiac and Cerebral Events. *JOURNAL OF CARDIOTHORACIC AND VASCULAR ANESTHESIA*. 2016;30(6):1461-1470. doi:10.1053/j.jvca.2016.05.046
24. Enc Y, Karaca P, Ayoglu U, Camur G, Kurc E, Cicek S. The acute cardioprotective effect of glucocorticoid in myocardial ischemia–reperfusion injury occurring during cardiopulmonary bypass. *Heart and vessels*. 2006;21:152-156.
25. Erdil N, Eroglu T, Akca B, et al. The effects of N-acetylcysteine on pulmonary functions in patients undergoing on-pump coronary artery surgery: a double blind placebo controlled study. *EUROPEAN REVIEW FOR MEDICAL AND PHARMACOLOGICAL SCIENCES*. 2016;20(1):180-187.
26. Eren N, Çakir Ö, Oruc A, Kaya Z, Erdinc L. Effects of N-acetylcysteine on pulmonary function in patients undergoing coronary artery bypass surgery with cardiopulmonary bypass. *Perfusion*. 2003;18(6):345-350.

27. Erkut B, Ates A. The Effect of Aspirin as an Irreversible COX1 Inhibitor in Preventing Non-Valvular Atrial Fibrillation After Coronary Bypass Surgery. *HEART SURGERY FORUM*. 2019;22(2):E149-E154. doi:10.1532/hsf.2111
28. Eslami M, Badkoubeh RS, Mousavi M, et al. Oral ascorbic acid in combination with beta-blockers is more effective than beta-blockers alone in the prevention of atrial fibrillation after coronary artery bypass grafting. *TEXAS HEART INSTITUTE JOURNAL*. 2007;34(3):268-274.
29. Farahani AV, Azar AY, Goodarzynejad HR, et al. Fish oil supplementation for primary prevention of atrial fibrillation after coronary artery bypass graft surgery: A randomized clinical trial. *INTERNATIONAL JOURNAL OF SURGERY*. 2017;42:41-48. doi:10.1016/j.ijsu.2017.04.025
30. Farquharson AL, Metcalf RG, Sanders P, et al. Effect of Dietary Fish Oil on Atrial Fibrillation After Cardiac Surgery. *AMERICAN JOURNAL OF CARDIOLOGY*. 2011;108(6):851-856. doi:10.1016/j.amjcard.2011.04.036
31. Feguri GR, de Lima PRL, de Cerqueira Borges D, et al. Preoperative carbohydrate load and intraoperatively infused omega-3 polyunsaturated fatty acids positively impact nosocomial morbidity after coronary artery bypass grafting: a double-blind controlled randomized trial. *Nutrition journal*. 2017;16(1):24. doi:10.1186/s12937-017-0245-6
32. Feguri GR, de Lima PRL, Franco AC, et al. Benefits of Fasting Abbreviation with Carbohydrates and Omega-3 Infusion During CABG: a Double-Blind Controlled Randomized Trial. *BRAZILIAN JOURNAL OF CARDIOVASCULAR SURGERY*. 2019;34(2):125-135. doi:10.21470/1678-9741-2018-0336
33. Gomez Polo JC, Vilacosta I, Martin-Garcia AC, et al. Use of corticosteroids in the prophylaxis of atrial fibrillation after cardiac surgery (ECOFA study). *European Heart Journal*. 2017;38:580-581. European Society of Cardiology, ESC Congress 2017. Barcelona Spain. doi:https://dx.doi.org/10.1093/eurheartj/ehx502.P2702
34. Halonen J, Halonen P, Jarvinen O, et al. Corticosteroids for the prevention of atrial fibrillation after cardiac surgery - A randomized controlled trial. *JAMA-JOURNAL OF THE AMERICAN MEDICAL ASSOCIATION*. 2007;297(14):1562-1567. doi:10.1001/jama.297.14.1562
35. Halvorsen P, Raeder J, White PF, et al. The effect of dexamethasone on side effects after coronary revascularization procedures. *Anesthesia and analgesia*. 2004;96(6):1578-1583.
36. Heidarsdottir R, Arnar DO, Skuladottir GV, et al. Does treatment with n-3 polyunsaturated fatty acids prevent atrial fibrillation after open heart surgery? *EUROPACE*. 2010;12(3):356-363. doi:10.1093/europace/eup429
37. Heidt MC, Vician M, Stracke SKH, et al. Beneficial Effects of Intravenously Administered N-3 Fatty Acids for the Prevention of Atrial Fibrillation after Coronary Artery Bypass Surgery: A Prospective Randomized Study. *THORACIC AND CARDIOVASCULAR SURGEON*. 2009;57(5):276-280. doi:10.1055/s-0029-1185301
38. Horbach SJ, Lopes RD, Guaragna J, et al. Naproxen as Prophylaxis against Atrial Fibrillation after Cardiac Surgery: The NAFARM Randomized Trial. *AMERICAN JOURNAL OF MEDICINE*. 2011;124(11):1036-1042. doi:10.1016/j.amjmed.2011.07.026
39. Jacob KA, Dieleman JM, Nathoe HM, et al. The effects of intraoperative dexamethasone on left atrial function and postoperative atrial fibrillation in cardiac surgical patients. *NETHERLANDS HEART JOURNAL*. 2015;23(3):168-173. doi:10.1007/s12471-014-0638-5

40. Ji Q, Mei YQ, Wang XS, et al. Effect of Preoperative Atorvastatin Therapy on Atrial Fibrillation Following Off-Pump Coronary Artery Bypass Grafting. *CIRCULATION JOURNAL*. 2009;73(12):2244-2249. doi:10.1253/circj.CJ-09-0352
41. Joss JD, Hernan J, Collier R, Cardenas A. Perioperative supplementation of polyunsaturated omega-3 fatty acid for the prevention of atrial fibrillation after cardiothoracic surgery. *AMERICAN JOURNAL OF HEALTH-SYSTEM PHARMACY*. 2017;74(1):E17-E23. doi:10.2146/ajhp150740
42. Kazemi B, Akbarzadeh F, Safaei N, Yaghoubi A, Shadvar K, Ghasemi K. Prophylactic High-Dose Oral-N-Acetylcysteine Does Not Prevent Atrial Fibrillation after Heart Surgery A Prospective Double Blind Placebo-Controlled Randomized Clinical Trial. *PACE-PACING AND CLINICAL ELECTROPHYSIOLOGY*. 2013;36(10):1211-1219. doi:10.1111/pace.12190
43. Kilger E, Heyn J, Beiras-Fernandez A, Luchting B, Weis F. Stress doses of hydrocortisone reduce systemic inflammatory response in patients undergoing cardiac surgery without cardiopulmonary bypass. *Minerva anestesiologica*. 2011;77(3):268-274.
44. KIM JC, HONG SW, SHIM JK, YOO KJ, CHUN DH, KWAK YL. Effect of N-acetylcystein on pulmonary function in patients undergoing off-pump coronary artery bypass surgery. *Acta anaesthesiologica scandinavica*. 2011;55(4):452-459.
45. Nikolayevich KV, Igorevna BO, Valentinovich YA, Sergeevna IA, Danilova E. Prevention of new-onset atrial fibrillation after direct myocardial revascularization surgery: randomized comparative study. *Медицинский вестник Северного Кавказа*. 2015;10(2 (38)):120-127.
46. Kourliouros A, Valencia O, Hosseini MT, et al. Preoperative high-dose atorvastatin for prevention of atrial fibrillation after cardiac surgery: A randomized controlled trial. *JOURNAL OF THORACIC AND CARDIOVASCULAR SURGERY*. 2011;141(1):244-248. doi:10.1016/j.jtcvs.2010.06.006
47. Lomivorotov VV, Efremov SM, Kalinichenko AP, et al. Methylprednisolone use is associated with endothelial cell activation following cardiac surgery. *Heart, Lung and Circulation*. 2013;22(1):25-30.
48. Lomivorotov V, Efremov S, Pokushalov E, Cherniavskiy A, Romanov A, Shilova A. Randomized trial of fish oil infusion to prevent atrial fibrillation after cardiac surgery: data from implantable continuous cardiac monitor. *Applied Cardiopulmonary Pathophysiology*. 2014;18:38-39. 29th Annual Meeting of the European Association of Cardiothoracic Anaesthesiologists, EACTA 2014 and 14th International Congress on Cardiovascular Anesthesia, ICCVA 2014. (29). Florence Italy. Sponsor: Abbott Vascular, abbvie, CSL Behring, Edwards Lifesciences, GE Healthcare, Philips Healthcare, MEDICA-Gruppe, Quartier ad fontes musica . Rameshkumar B.S., Manners J. (146 pages).
49. Mannacio VA, Iorio D, De Amicis V, Di Lello F, Musumeci F. Effect of rosuvastatin pretreatment on myocardial damage after coronary surgery: a randomized trial. *The Journal of thoracic and cardiovascular surgery*. 2008;136(6):1541-1548.
50. Mansour H, Ghaleb R. Atorvastatin for reduction of postoperative atrial fibrillation in patients undergoing cardiac surgery. *Atherosclerosis supplements*. 2017;25:e2-. doi:10.1016/j.atherosclerosissup.2017.03.004
51. Mardani D, Bigdelian H. Prophylaxis of dexamethasone protects patients from further post-operative delirium after cardiac surgery: a randomized trial. *Journal of research in medical sciences: the official journal of Isfahan University of Medical Sciences*. 2013;18(2):137.

52. Mirhosseini SJ, Forouzannia SK, Sayegh AH, Sanatkar M. Effect of prophylactic low dose of methylprednisolone on postoperative new atrial fibrillation and early complications in patients with severe LV dysfunction undergoing elective off-pump coronary artery bypass surgery. *Acta medica Iranica*. 2011;49(5):288-292.
53. Mirmohammadsadeghi M, Mirmohammadsadeghi A, Mahmoudian M. Preventive Use of Ascorbic Acid For Atrial Fibrillation After Coronary Artery Bypass Graft Surgery. *The heart surgery forum*. 2018;21(5):E415-E417. doi:https://dx.doi.org/10.1532/hsf.1938
54. Moludi J, Keshavarz S, Pakzad R, Sedghi N, Sadeghi T, Alimoradi F. Effect of vitamin C supplementation in the prevention of atrial fibrillation. *Tehran University Medical Journal*. 2016;73(11):791-797.
55. Orhan G, Yapici N, Yuksel M, et al. Effects of N-acetylcysteine on myocardial ischemia–reperfusion injury in bypass surgery. *Heart and vessels*. 2006;21:42-47.
56. Ozaydin M, Peker O, Erdogan D, et al. N-acetylcysteine for the prevention of postoperative atrial fibrillation: a prospective, randomized, placebo-controlled pilot study. *EUROPEAN HEART JOURNAL*. 2008;29(5):625-631. doi:10.1093/eurheartj/ehn011
57. Papoulidis P, Ananiadou O, Chalvatzoulis E, et al. The role of ascorbic acid in the prevention of atrial fibrillation after elective on-pump myocardial revascularization surgery: a single-center experience - a pilot study. *INTERACTIVE CARDIOVASCULAR AND THORACIC SURGERY*. 2011;12(2):121-124. doi:10.1510/icvts.2010.240473
58. Patti G, Chello M, Candura D, et al. Randomized trial of atorvastatin for reduction of postoperative atrial fibrillation in patients undergoing cardiac surgery - Results of the ARMYDA-3 (Atorvastatin for reduction of MYocardial dysrhythmia after cardiac surgery) study. *CIRCULATION*. 2006;114(14):1455-1461. doi:10.1161/CIRCULATIONAHA.106.621763
59. Pierri MD, Crescenzi G, Zingaro C, et al. Prevention of atrial fibrillation and inflammatory response after on-pump coronary artery bypass using different statin dosages: a randomized, controlled trial. *GENERAL THORACIC AND CARDIOVASCULAR SURGERY*. 2016;64(7):395-402. doi:10.1007/s11748-016-0647-y
60. Prasongsukarn K, Abel JG, Jamieson WRE, et al. The effects of steroids on the occurrence of postoperative atrial fibrillation after coronary artery bypass grafting surgery: A prospective randomized trial. *JOURNAL OF THORACIC AND CARDIOVASCULAR SURGERY*. 2005;130(1):93-98. doi:10.1016/j.jtcvs.2004.09.014
61. Rodrigo R, Korantzopoulos P, Cereceda M, et al. A Randomized Controlled Trial to Prevent Post-Operative Atrial Fibrillation by Antioxidant Reinforcement. *JOURNAL OF THE AMERICAN COLLEGE OF CARDIOLOGY*. 2013;62(16):1457-1465. doi:10.1016/j.jacc.2013.07.014
62. Rubanenko OA. Efficacy of atorvastatin therapy in prevention of postoperative atrial fibrillation in patients with ischemic heart disease. *Rational pharmacotherapy in cardiology*. 2015;11(5):464-469.
63. Rubens FD, Nathan H, Labow R, et al. Effects of methylprednisolone and a biocompatible copolymer circuit on blood activation during cardiopulmonary bypass. *ANNALS OF THORACIC SURGERY*. 2005;79(2):655-665. doi:10.1016/j.athoracsur.2004.07.044

64. Sadeghpour A, Alizadehasl A, Kyavar M, et al. Impact of vitamin C supplementation on post-cardiac surgery ICU and hospital length of stay. *Anesthesiology and pain medicine*. 2015;5(1)
65. Samadikhah J, Golzari SEJ, Sabermarouf B, et al. Efficacy of combination therapy of statin and vitamin C in comparison with statin in the prevention of post-CABG atrial fibrillation. *Advanced pharmaceutical bulletin*. 2014;4(1):97-100. doi:10.5681/apb.2014.015
66. Saravanan P, Bridgewater B, West AL, O'Neill SC, Calder PC, Davidson NC. Omega-3 Fatty Acid Supplementation Does Not Reduce Risk of Atrial Fibrillation After Coronary Artery Bypass Surgery A Randomized, Double-Blind, Placebo-Controlled Clinical Trial. *CIRCULATION-ARRHYTHMIA AND ELECTROPHYSIOLOGY*. 2010;3(1):46-53. doi:10.1161/CIRCEP.109.899633
67. Saravanan P, West AL, Bridgewater B, et al. Omega-3 fatty acids do not alter P-wave parameters in electrocardiogram or expression of atrial connexins in patients undergoing coronary artery bypass surgery. *EUROPACE*. 2016;18(10):1521-1527. doi:10.1093/europace/euv398
68. Sarzaeem M, Shayan N. Vitamin C in prevention of atrial fibrillation after coronary artery bypass graft: Double blind randomized clinical trial. *Tehran University Medical Journal*. 2014;71(12):787-793.
69. Sarzaeem M, Shayan N, Bagheri J, Jebelli M, Mandegar M. Low dose Colchicine in prevention of atrial fibrillation after coronary artery bypass graft: a double blind clinical trial. *Tehran university medical journal*. 2014;72(3):147-154.
70. Schurr UP, Zünd G, Hoerstrup SP, et al. Preoperative administration of steroids: influence on adhesion molecules and cytokines after cardiopulmonary bypass. *The Annals of thoracic surgery*. 2001;72(4):1316-1320.
71. Sobieski MA, Graham JD, Pappas PS, Tatooles AJ, Slaughter MS. Reducing the effects of the systemic inflammatory response to cardiopulmonary bypass: can single dose steroids blunt systemic inflammatory response syndrome? *ASAIO Journal*. 2008;54(2):203-206.
72. Soleimani A, Habibi MR, Kiabi FH, et al. The effect of intravenous N-acetylcysteine on prevention of atrial fibrillation after coronary artery bypass graft surgery: a double-blind, randomised, placebo-controlled trial. *KARDIOLOGIA POLSKA*. 2018;76(1):99-106. doi:10.5603/KP.a2017.0183
73. Song YB, On YK, Kim JH, et al. The effects of atorvastatin on the occurrence of postoperative atrial fibrillation after off-pump coronary artery bypass grafting surgery. *AMERICAN HEART JOURNAL*. 2008;156(2)doi:10.1016/j.ahj.200804.020
74. Sorice M, Tritto FP, Sordelli C, Gregorio R, Piazza L. N-3 polyunsaturated fatty acids reduces post-operative atrial fibrillation incidence in patients undergoing "on-pump" coronary artery bypass graft surgery. *Monaldi archives for chest disease - cardiac series 76 (2) (pp 93-98), 2011 Date of publication: 2011*. 2011;doi:10.4081/monaldi.2011.196
75. Stanger O, Aigner I, Schimetta W, Wonisch W. Antioxidant supplementation attenuates oxidative stress in patients undergoing coronary artery bypass graft surgery. *Tohoku journal of experimental medicine*. 2014;232(2):145-154. doi:10.1620/tjem.232.145
76. Suezawa T, Aoki A, Kotani M, et al. Clinical benefits of methylprednisolone in off-pump coronary artery bypass surgery. *General thoracic and cardiovascular surgery*. 2013;61:455-459.

77. Sun YF, Ji QA, Mei YQ, et al. Role of Preoperative Atorvastatin Administration in Protection Against Postoperative Atrial Fibrillation Following Conventional Coronary Artery Bypass Grafting. *INTERNATIONAL HEART JOURNAL*. 2011;52(1):7-11. doi:10.1536/ihj.52.7
78. Tabbalat RA, Hamad NM, Alhaddad IA, Hammoudeh AJ, Akasheh BF, Khader YS. Effect of colchiciNe on the inciDence of atrial fibrillation in open heart surgery patients: end-AF trial. *European heart journal*. 2015;36:915. doi:10.1093/eurheartj/ehv401
79. Tabbalat RA, Alhaddad I, Hammoudeh A, et al. Effect of Low-dose ColchiciNe on the InciDence of Atrial Fibrillation in Open Heart Surgery Patients: END-AF Low Dose Trial. *JOURNAL OF INTERNATIONAL MEDICAL RESEARCH*. 2020;48(7)doi:10.1177/0300060520939832
80. Vukovic PM, Maravic-Stojkovic VR, Peric MS, et al. Steroids and statins: an old and a new anti-inflammatory strategy compared. *PERFUSION-UK*. 2011;26(1):31-37. doi:10.1177/0267659110385607
81. Wang W, Yuan XH. Clinical efficacy of epicardial application with an optimal dosage of amiodarone-releasing hydrogels to prevent postoperative atrial fibrillation. *Journal of the American College of Cardiology*. 2016;68(16):C117-C118.
82. Wang DY, Wang M, Zhang H, Zhu H, Zhang N, Liu JD. Effect of Intravenous Injection of Vitamin C on Postoperative Pulmonary Complications in Patients Undergoing Cardiac Surgery: A Double-Blind, Randomized Trial. *DRUG DESIGN DEVELOPMENT AND THERAPY*. 2020;14:3263-3270. doi:10.2147/DDDT.S254150
83. Weis F, Beiras-Fernandez A, Schelling G, et al. Stress doses of hydrocortisone in high-risk patients undergoing cardiac surgery: Effects on interleukin-6 to interleukin-10 ratio and early outcome. *CRITICAL CARE MEDICINE*. 2009;37(5):1685-1690. doi:10.1097/CCM.0b013e31819fca77
84. Wijeysundera DN, Beattie WS, Rao V, Granton JT, Chan CT. N-acetylcysteine for preventing acute kidney injury in cardiac surgery patients with pre-existing moderate renal insufficiency. *CANADIAN JOURNAL OF ANAESTHESIA-JOURNAL CANADIEN D ANESTHESIE*. 2007;54(11):872-881. doi:10.1007/BF03026790
85. Wilbring M, Plotze K, Bormann S, Waldow T, Matschke K. Omega-3 polyunsaturated fatty acids reduce the incidence of postoperative atrial fibrillation in patients with history of prior myocardial infarction undergoing isolated coronary artery bypass grafting. *Thoracic and cardiovascular surgeon*. 2014;62doi:10.1055/s-0034-1367437
86. Yamamoto T. Protective effect of eicosapentaenoic acid on insulin resistance in hyperlipidemic patients and on the postoperative course of cardiac surgery patients: The possible involvement of adiponectin. 2014;
87. Yared JP, Starr NJ, Torres FK, et al. Effects of single dose, postinduction dexamethasone on recovery after cardiac surgery. *ANNALS OF THORACIC SURGERY*. 2000;69(5):1420-1424. doi:10.1016/S0003-4975(00)01180-2
88. Yared JP, Bakri MH, Erzurum SC, et al. Effect of Dexamethasone on Atrial Fibrillation After Cardiac Surgery: Prospective, Randomized, Double-Blind, Placebo-Controlled Trial. *Journal of Cardiothoracic and Vascular Anesthesia*. 2007;21(1):68-75. doi:https://dx.doi.org/10.1053/j.jvca.2005.10.014
89. Zarpelon CS, Netto MC, Jorge JCM, et al. Colchicine to Reduce Atrial Fibrillation in the Postoperative Period of Myocardial Revascularization. *ARQUIVOS BRASILEIROS DE CARDIOLOGIA*. 2016;107(1):4-8. doi:10.5935/abc.20160082

90. Zheng Z, Jayaram R, Jiang L, et al. Perioperative rosuvastatin in cardiac surgery. *New England Journal of Medicine*. 2016;374(18):1744-1753.

Supplemental Appendix S1; PRISMA NMA Checklist of Items to Include When Reporting A Systematic Review Involving a Network Meta-analysis

Section/Topic	Item #	Checklist Item	Reported on Page #
**TITLE**			
Title	1	Identify the report as a systematic review *incorporating a network meta-analysis (or related form of meta-analysis).*	***Page 1, line 1-4***
**ABSTRACT**			
Structured summary	2	Provide a structured summary including, as applicable:<br>**Background:** main objectives<br>**Methods:** data sources; study eligibility criteria, participants, and interventions; study appraisal; and *synthesis methods, such as network meta-analysis.*<br>**Results:** number of studies and participants identified; summary estimates with corresponding confidence/credible intervals; *treatment rankings may also be discussed. Authors may choose to summarize pairwise comparisons against a chosen treatment included in their analyses for brevity.*<br>**Discussion/Conclusions:** limitations; conclusions and implications of findings.<br>**Other:** primary source of funding; systematic review registration number with registry name.	***Page 4, line 47-64***
**INTRODUCTION**			
Rationale	3	Describe the rationale for the review in the context of what is already known, *including mention of why a network meta-analysis has been conducted.*	***Page 5, line 65-83***
Objectives	4	Provide an explicit statement of questions being addressed, with reference to participants, interventions, comparisons, outcomes, and study design (PICOS).	***Page 5, line 83-85***
**METHODS**			
Protocol and registration	5	Indicate whether a review protocol exists and if and where it can be accessed (e.g., Web address); and, if available, provide registration information, including registration number.	***Page 6, line 88-89***
Eligibility criteria	6	Specify study characteristics (e.g., PICOS, length of follow-up) and report characteristics (e.g., years considered, language, publication status) used as criteria for eligibility, giving rationale. *Clearly describe eligible treatments*	***Page 6,7 line 104-110***

		included in the treatment network, and note whether any have been clustered or merged into the same node (with justification).	
Information sources	7	Describe all information sources (e.g., databases with dates of coverage, contact with study authors to identify additional studies) in the search and date last searched.	***Page 6, line 95-99***
Search	8	Present full electronic search strategy for at least one database, including any limits used, such that it could be repeated.	***Page 7, line 111, 112, and Table S2***
Study selection	9	State the process for selecting studies (i.e., screening, eligibility, included in systematic review, and, if applicable, included in the meta-analysis).	***Page 7, line 111-114***
Data collection process	10	Describe method of data extraction from reports (e.g., piloted forms, independently, in duplicate) and any processes for obtaining and confirming data from investigators.	***Page 7, line 115-123***
Data items	11	List and define all variables for which data were sought (e.g., PICOS, funding sources) and any assumptions and simplifications made.	***Page 7, line 118-121***
**Geometry of the network**	**S1**	Describe methods used to explore the geometry of the treatment network under study and potential biases related to it. This should include how the evidence base has been graphically summarized for presentation, and what characteristics were compiled and used to describe the evidence base to readers.	***Page 8, line 14-141, 149-150***
Risk of bias within individual studies	12	Describe methods used for assessing risk of bias of individual studies (including specification of whether this was done at the study or outcome level), and how this information is to be used in any data synthesis.	***Page 7, 8, line 124-131***
Summary measures	13	State the principal summary measures (e.g., risk ratio, difference in means). *Also describe the use of additional summary measures assessed, such as treatment rankings and surface under the cumulative ranking curve (SUCRA) values, as well as modified approaches used to present summary findings from meta-analyses.*	***Page 8, 9, line 151-154***
Planned methods of analysis	14	Describe the methods of handling data and combining results of studies for each network meta-analysis. This should include, but not be limited to:<br>• *Handling of multi-arm trials;*<br>• *Selection of variance structure;*<br>• *Selection of prior distributions in Bayesian analyses; and*<br>• *Assessment of model fit.*	***Page 8, line 133-140***
**Assessment of Inconsistency**	**S2**	Describe the statistical methods used to evaluate the agreement of direct and indirect evidence in the treatment network(s) studied. Describe efforts taken to address its presence when found.	***Page 8, line 143***
Risk of bias across studies	15	Specify any assessment of risk of bias that may affect the cumulative evidence (e.g., publication bias, selective reporting within studies).	***Page 8, line 144-148***
Additional analyses	16	Describe methods of additional analyses if done, indicating which were pre-specified. This may include, but not be limited to, the following:	***Page 9, line 155-157***

- Sensitivity or subgroup analyses;
- Meta-regression analyses;
- *Alternative formulations of the treatment network; and*
- *Use of alternative prior distributions for Bayesian analyses (if applicable).*

RESULTS

Study selection	17	Give numbers of studies screened, assessed for eligibility, and included in the review, with reasons for exclusions at each stage, ideally with a flow diagram.	***Page 10, line 176-182***
**Presentation of network structure**	**S3**	Provide a network graph of the included studies to enable visualization of the geometry of the treatment network.	***Figure 2 and Figures S3, S4, S5***
**Summary of network geometry**	**S4**	Provide a brief overview of characteristics of the treatment network. This may include commentary on the abundance of trials and randomized patients for the different interventions and pairwise comparisons in the network, gaps of evidence in the treatment network, and potential biases reflected by the network structure.	***Page 11, line 206-209***
Study characteristics	18	For each study, present characteristics for which data were extracted (e.g., study size, PICOS, follow-up period) and provide the citations.	***Page 10, line 184-190***
Risk of bias within studies	19	Present data on risk of bias of each study and, if available, any outcome level assessment.	***Page 11, line 199-204 Figure S1***
Results of individual studies	20	For all outcomes considered (benefits or harms), present, for each study: 1) simple summary data for each intervention group, and 2) effect estimates and confidence intervals. *Modified approaches may be needed to deal with information from larger networks.*	***Table S4***
Synthesis of results	21	Present results of each meta-analysis done, including confidence/credible intervals. *In larger networks, authors may focus on comparisons versus a particular comparator (e.g. placebo or standard care), with full findings presented in an appendix. League tables and forest plots may be considered to summarize pairwise comparisons.* If additional summary measures were explored (such as treatment rankings), these should also be presented.	***Page 11-14 line 217-249***
**Exploration for inconsistency**	**S5**	Describe results from investigations of inconsistency. This may include such information as measures of model fit to compare consistency and inconsistency models, *P* values from statistical tests, or summary of inconsistency estimates from different parts of the treatment network.	***Page 12, line 210, 211, (Figures S6, S7, and S8)***
Risk of bias across studies	22	Present results of any assessment of risk of bias across studies for the evidence base being studied.	***Page 11, line 211-216***
Results of additional analyses	23	Give results of additional analyses, if done (e.g., sensitivity or subgroup analyses, meta-regression analyses, *alternative network geometries studied, alternative choice of prior distributions for Bayesian analyses*, and so forth).	***Page 14 line 250-255***

**DISCUSSION**			
Summary of evidence	24	Summarize the main findings, including the strength of evidence for each main outcome; consider their relevance to key groups (e.g., healthcare providers, users, and policy-makers).	***Page 15, line 257-267***
Limitations	25	Discuss limitations at study and outcome level (e.g., risk of bias), and at review level (e.g., incomplete retrieval of identified research, reporting bias). *Comment on the validity of the assumptions, such as transitivity and consistency. Comment on any concerns regarding network geometry (e.g., avoidance of certain comparisons).*	***Page 17, line 306-314***
Conclusions	26	Provide a general interpretation of the results in the context of other evidence, and implications for future research.	***Page 18, line 333-338***
**FUNDING**			
Funding	27	Describe sources of funding for the systematic review and other support (e.g., supply of data); role of funders for the systematic review. This should also include information regarding whether funding has been received from manufacturers of treatments in the network and/or whether some of the authors are content experts with professional conflicts of interest that could affect use of treatments in the network.	***Page 19, line 348***

PICOS = population, intervention, comparators, outcomes, study design.
* Text in italics indicates wording specific to reporting of network meta-analyses that has been added to guidance from the PRISMA statement.
